# A Density Functional Theory‐Based Investigation of a pH‐ and Redox‐Driven Tristable [2]Rotaxane in CH_2_Cl_2_ Dilute Solution

**DOI:** 10.1002/open.70207

**Published:** 2026-04-23

**Authors:** Costantino Zazza, Nico Sanna, Stefano Borocci, Felice Grandinetti

**Affiliations:** ^1^ Department for Innovation in Biological Agro‐food and Forest systems Università della Tuscia (DIBAF) Viterbo Italy; ^2^ Istituto per la Scienza e Tecnologia dei Plasmi del CNR (ISTP) Bari Italy; ^3^ Istituto per i Sistemi Biologici del CNR (ISB) Sede di Roma ‐ Meccanismi di Reazione c/o Dipartimento di Chimica Sapienza Università di Roma Rome Italy

**Keywords:** density functional theory, molecular rotaxanes, nanoscale devices, quantum theory of atoms in molecules

## Abstract

A recently synthesized pH‐ and redox‐driven tristable [2]rotaxane in dichloromethane solution (*Angew. Chem.* **2025**, 64, e202414609) has been investigated within the framework of the Density Functional Theory DFT) in a polarizable and dielectric media via self‐consistent reaction field method. Optimized molecular species are subsequently analyzed by combining converged wavefunctions with Quantum Theory of Atoms in Molecules (QTAIM) and the Independent Gradient Model based on Hirshfeld partition (IGMH) algorithms to characterize the nature of the chemical interactions modulating the preferential position of a 24‐crown‐8 (**DB24C8**) macrocycle over a responsive molecular thread containing three potential recognition moieties: an ammonium (AmH^+^), a bipyridinium (Bpy^2+^), and a triazolium (Trz^+^) moiety. Interestingly, the herein proposed computational investigation, while supporting the spectroscopically observed (^1^H NMR, 500 MHz, 298K) stable species under different equilibrium conditions, also sheds some light on the nature of the chemical interactions finely modulating the selective complexation pathways and the emerging shaping in dichloromethane.

## Graphical abstract

A recently proposed pH‐ and redox‐driven tristable [2]rotaxane in CH_2_Cl_2_ dilute solution is investigated, combining converged Density Functional Theory (DFT)‐based electronic eigenstates. The theoretical picture that has emerged allows to identify the local supramolecular interaction patterns capable of modulating the mutual position of a DB24C8 macrocycle over a molecular thread featuring three discretionary active stations.

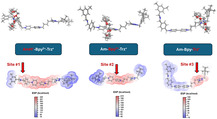

## 1 Introduction

Molecular rotaxanes, consisting of a linear axle threaded through a macrocyclic ring and capped with bulky stoppers to prevent dethreading, represent one of the most emblematic classes of mechanically interlocked molecules (MIMs) [1, 2, 3]. Since their initial development, rotaxanes have emerged as versatile platforms for exploring fundamental aspects of supramolecular chemistry, molecular recognition, and controlled motion at the nanoscale [4, 5, 6]. Their unique topology, in which mechanical bonds rather than covalent linkages dictate architecture, endows them with properties not attainable in conventional molecular systems. A central feature of rotaxanes is their ability to undergo well‐defined translational or conformational motion in response to external stimuli, including pH, redox state, light, or metal coordination [7, 8, 9, 10]. This responsiveness has been widely exploited in the design of molecular switches, shuttles, and machines, with potential applications in molecular electronics, smart materials, and responsive catalysis. While much attention has been directed toward rotaxanes in the solid state and on surfaces, their behavior in solution is equally critical for both fundamental understanding and functional deployment [11, 12, 13, 14, 15, 16, 17]. Solution‐phase studies provide insight into dynamic equilibria, energetics of ring–axle interactions, and the influence of solvent and ionic environments on mechanical motion [18, 19, 20, 21, 22, 23, 24]. Despite significant advances, a number of challenges remain in elucidating and harnessing the solution‐phase properties of rotaxanes. These include the quantitative characterization of co‐conformational dynamics, the balance between enthalpic and entropic contributions to mechanical stability, and the development of robust design principles for predictable switching behavior [25, 26, 27, 28, 29, 30]. Addressing these challenges is essential for translating rotaxane‐based architectures from conceptual molecular machines into practical technologies.

**SCHEME 1:**
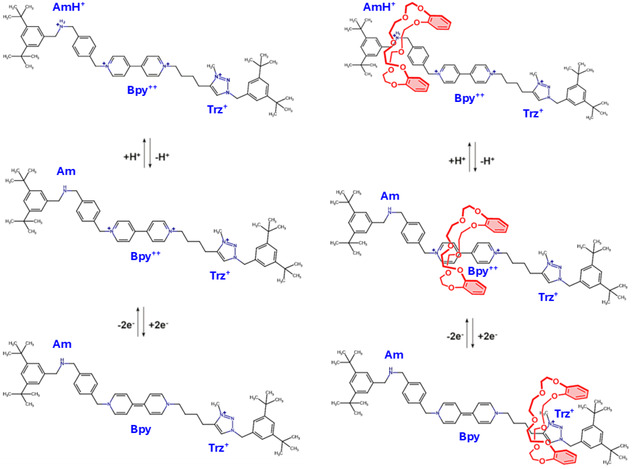
The investigated pH‐ and redox‐driven tristable [2]rotaxane as proposed by A. Credi and coworkers in Ref. 31. The ammonium (Am^+^) and bipyridinium (Bpy^2+^) stations being responsive to orthogonal stimuli—pH variation and selective electrochemical reduction—allow a direct control in CH_2_Cl_2_ over the macrocycle (**DB24C8**) movement along the functionalized “three stations” axle.

In this emerging and fascination research field, our attention has been drawn to a recently synthesized pH‐ and redox‐driven tristable [2]rotaxane [31] composed by a dibenzo‐24‐crown‐8 (**DB24C8**) macrocyclic component mechanically interlocked over a functionalized molecular thread mainly containing three different recognition sites: ammonium (AmH^+^), bipyridinium (Bpy^2+^), and triazolium (Trz^+^) moieties. A. Credi et al. in this research contribution unequivocally reported that AmH^+^ and Bpy^2+^ chemical units actually allow a direct control of the directional shuttling of the **DB24C8** crown ether via fully orthogonal pH and electrochemical stimuli enabling two sequential translation steps along the same direction (from AmH^+^ to Trz^+^ and vice versa, see Scheme 1) [31]. In this respect, selective host or guest mutual noncovalent interactions as well as the reversible character of the punctual switching processes are seen to play a key role in the proposed synthetic assembly working as an orthogonal stimuli‐responsive tristable rotaxane in a non‐polar solvent (e.g., dichloromethane). With the target of fully characterizing the driving forces modulating the observed discretional **DB24C8**/station affinity, herein we propose a computational investigation of such a fascinating synthetic supramolecular aggregate which combines converged electronic Density Functional Theory (DFT)‐based wavefunctions [32] with Quantum Theory of Atoms in Molecules (QTAIM) [33, 34, 35] analytical descriptors. To this end, the spectroscopically observed stable supramolecular species [AmH^+^(**DB24C8**); Bpy^2+^(**DB24C8**); Trz^+^(**DB24C8**)] are fully analyzed by means of electron density *ρ*(***r***), the associated Laplacian [*∇* ^2^ *ρ*(***r***)], and local electronic energy density *H*(***r***), according to a Bader's topology analysis [33, 34]. Optimized DFT molecular structures in CH_2_Cl_2_ dilute solution, the derived QTAIM analytical descriptors, and the graphical representation of the supramolecular interactions via noncovalent interaction (NCI) analysis, while supporting the asymmetrical eigenstates observed contextually shed some light on the nature of the chemical interactions finely modulating the shuttling process of the **DB24C8** macrocycle over the responsive AmH^+^‐Bpy^2+^‐Trz^+^ axle. Finally, in a more general context, the present contribution once again highlights that the intrinsic complexity of designing soft‐matter nanoscale machines and motors with improved efficiencies and emerging functionalities can benefit from a direct interplay of theoretical modeling and experiments.

## 2 Computational Details

At first, we started optimizing the molecular structure of the functionalized AmH^+^‐Bpy^2+^‐Trz^+^ molecular thread in a polarizable and continuum media modeling an encircling CH_2_Cl_2_ solvent environment. More specifically, the theoretical level employed in this study was the DFT [32] adopting the hybrid Becke 3‐parameters Lee–Yang–Parr (B3LYP) functional [36, 37] and its Coulomb‐Attenuating Method formulation (CAM‐B3LYP) [38] in conjunction with the 6‐311G** Gaussian‐based atomic basis set [39]. Also, we used Grimme's dispersion correction as an add‐on to standard Kohn–Sham (KS) equations with the original D3 damping function (i.e., DFT‐D3) as proposed in Ref. [40] to account—in a computationally efficient way—for the van der Waals (vdW) interactions correction in large molecular systems. In this respect, inspired by experimental conditions [31], we considered the investigated synthetic thread in three different states: *i*) AmH^+^‐Bpy^2+^‐Trz^+^ *ii*) Am‐Bpy^2+^‐Trz^+^ *iii*) Am‐Bpy‐Trz^+^. These molecules were relaxed by applying a redundant‐based internal coordinates algorithm in a dielectric and polarizable CH_2_Cl_2_ solution modeled via the popular conductor‐like polarizable continuum model (C‐PCM) model [41]. Furthermore, with the target of estimating—for the first time—the lowest valence UV‐Vis absorption bands of these species, we used a Time‐Dependent (TD‐DFT) formalism [42, 43]. The absorption bands are then estimated by combining the emerging vertical excitation energies with oscillator strengths and using Gaussian broadening functions having a full width at half maximum (FWHM) of 3000 cm^−1^ (i.e., the Δ*v’*_*1/2*_) [44]. The formula used to convolute the computed TD‐DFT(C‐PCM/B3LYP//CAM‐B3LYP) UV‐Vis absorption spectra is given as follows:

(1) $$\varepsilon \left(v\prime \right)=\frac{2.175\cdot 10^{8}}{\Delta v_{\frac{1}{2}}^{\prime }}\cdot f\cdot exp\left[-2.772\cdot \frac{{\left(v\prime -v_{i\to f}^{\prime }\right)}^{2}}{\Delta v_{\frac{1}{2}}^{\prime }}\right]$$

where *f* (the oscillator strength under the transition dipole length approximation) and *v’*_*i→f*_ (the vertical excitation energy in wavenumbers, cm^−1^) are both directly extracted from TD‐DFT calculations. The oscillator strength is related to the transition dipole moment, *M*_*i→f*_, by the standard equation:

(2) $$f_{i\to f}=\frac{8\pi m_{e}}{3e^{2}h^{2}}\cdot \Delta E_{i\to f}\cdot {\left|M_{i\to f}\right|}^{2}=\frac{8\pi m_{e}c}{3e^{2}h^{2}}\cdot v_{i\to f}^{'}\cdot {\left|M_{i\to f}\right|}^{2}$$

where *m*_*e*_ and *e* are the mass and the charge of the electron, respectively, *h* is Planck's constant, and *c* is the speed of light. The energy difference (Δ*E*_*i→f*_) between these states is related to the frequency *ν*, the wavelength *λ*, and the wavenumber *ν’* of absorbed light by the equation:

(3) $$\Delta E_{i\to f}=h\cdot v_{i\to f}=h\cdot \frac{c}{\lambda_{i\to f}}=h\cdot c\cdot v_{i\to f}^{'}$$

The results of TD‐DFT calculations are then convoluted using Gaussian‐type curves to create the associated UV‐Vis spectra; more specifically, each electronic transition is associated with a Gaussian curve according to the expression of the *ε*(*v’*) value, which provides an estimation of the molar absorption coefficient, in M^−1^ cm^−1^, as reported in Equation (1) [44, 45].

As a next step, we addressed the nature of the supramolecular interactions, finely modulating the supramolecular assembly in a CH_2_Cl_2_ solution [31] with a topological description within the QTAIM framework [33, 34, 35]. As already successfully applied over [2]rotaxane molecular shuttles [46, 47], the derived DFT‐based indicators, at C‐PCM/B3LYP(D3)/6‐311G** level of computation, mainly include the Kohn–Sham (K‐S) electron density *ρ*(***r***) [40], the electronic energy density *H*(***r***) and its kinetic and potential components *G*(***r***) and *V*(***r***), respectively, as well as the Reduced Density Gradient (RDG) *s*(***r***). The *ρ*(***r***) is defined as usual by the equation:



(4)

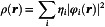

where $\eta_{i}$ is the occupation number of the natural orbital $\varphi_{i}$, in turn expanded as a linear combination of the atomic basis functions [32, 33, 34, 35]. The *H*(***r***) corresponds with the sum of the kinetic energy density *G*(***r***) and the potential energy density *V*(***r***):

(5) H(r)=G(r)+V(r)

where the presently employed definition of the *G*(***r***) is given by the following equation:



(6)

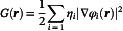




with the sum running over all the occupied natural orbitals $\varphi_{i}$ of occupation numbers $\eta_{i}$. The potential energy density *V*(***r***) is evaluated [33, 34, 35] from the local form of the virial theorem:

(7) $$V\left(r\right)=\frac{1}{4}\nabla^{2}\rho \left(r\right)-2G\left(r\right)$$

At last, the topological analysis within the QTAIM framework is also supported by a visual characterization of NCIs via the independent gradient model (IGM) [46], on converged C‐PCM/DFT electronic wavefunctions, based on Hirshfeld partition of molecular density (IGMH) [47, 48] as implemented in the Multiwfn toolbox [49, 50, 51, 52]. Thus, interactions patterns are conveniently visualized considering the optimized structure, at C‐PCM/B3LYP(D3)/6‐311G** level of computation, of the investigated supramolecular aggregates [i.e., AmH^+^(**DB24C8**)‐Bpy^2+^‐Trz^+^, Am‐Bpy^2+^(**DB24C8**)‐Trz^+^, Am‐Bpy‐Trz^+^(**DB24C8**)] in a 3D space by plotting a chosen low‐value isosurface of the $\delta g_{\text{Hirsh}}^{\text{inter}}$, colored by the *sign*(*λ*_2_)×*ρ*(*r*) (colors range from blue to red for attractive to repulsive interactions, respectively). TD‐DFT and DFT computations were performed using the Gaussian 16 code (Rev. C01) [53], while QTAIM and IGMH analyses were performed with the support of the Visual Molecular Dynamics (VMD) program [54]. At last, we would like to mention that we also carried out, for the synthetic AmH^+^‐Bpy^2+^‐Trz^+^, Am‐Bpy^2+^‐Trz^+^, and Am‐Bpy‐Trz^+^ molecular threads, electrostatic surface potential (ESP) calculations [55] with the target of estimating the partial distribution of charge along the molecular wire following the imposed pH variation and electrochemical reduction (see Scheme 1) [31].

## 3 Results and Discussion

### 3.1 UV‐Vis Adsorption and Electrostatic Properties

**FIGURE 1:**
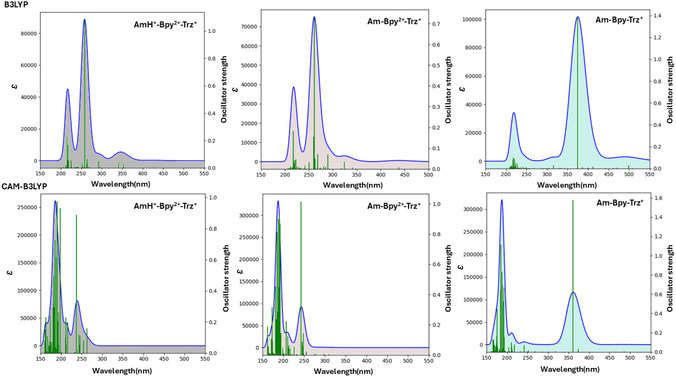
UV/Vis absorption spectra of the AmH^+^‐Bpy^2+^‐Trz^+^(left panel), Am‐Bpy^2+^‐Trz^+^ (middle panel), and Am‐Bpy‐Trz^+^ (right panel) forms of the investigated molecular thread at C‐PCM(CH_2_Cl_2_)/B3LYP(D3)//CAM‐B3LYP(D3)/6‐311G** level of computations. The molar absorption coefficient (ε) is reported in M^−1^cm^−1^; oscillator strengths are also reported as green histograms.

**FIGURE 2:**
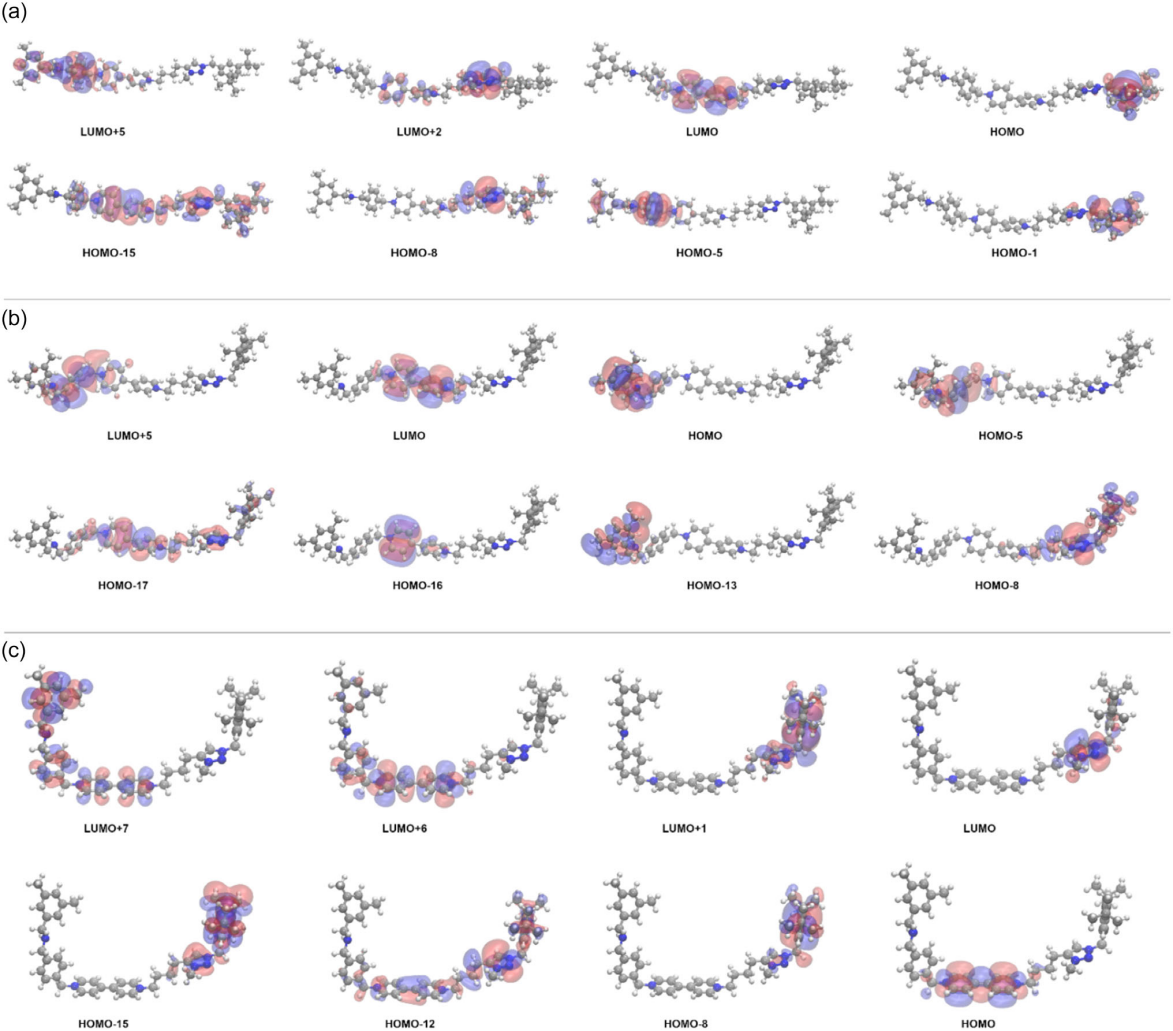
(a) K‐S molecular orbitals ‐ at C‐PCM(CH_2_Cl_2_)/B3LYP(D3)/6‐311G** ‐ from the optimized structure of the AmH^+^‐Bpy^2+^‐Trz^+^ thread responsible for singlet transitions showing oscillator strength in the range between 0.034 and 1.084 (see Table S1). The blue color reflects the positive (+) part of the corresponding eigenvector and an isodensity value of 0.015 *e*·*a*_0_^−3^ is used. HOMO stands for the highest occupied molecular orbital and LUMO for the lowest unoccupied molecular orbital. (b) The same as a) but for the Am‐Bpy^2+^‐Trz^+^ form. The associated transitions feature oscillator strength between 0.073 and 0.732 (see Table S2). (c) The same as a) but for the Am‐Bpy‐Trz^+^ reduced form, oscillator strength between 0.076 and 1.386 (see Table S3).

The UV‐Vis adsorption spectra, within the C‐PCM(CH_2_Cl_2_)/TD‐DFT framework, following the relaxation procedure of the AmH^+^‐Bpy^2+^‐Trz^+^, Am‐Bpy^2+^‐Trz^+^, and Am‐Bpy‐Trz^+^ suggested forms of the investigated molecular thread are explicitly reported in Figure 1. Looking at this figure, it is evident that—at least within the explored spectral window—the selective electrochemical reduction of the Bpy unit (from Bpy^2+^ to Bpy) strongly influences the absorption pathways estimated. As a matter of fact, the AmH^+^‐Bpy^2+^‐Trz^+^ and Am‐Bpy^2+^‐Trz^+^ species feature very similar UV‐Vis absorption spectra with a main band centered at around 250 nm and a second adsorption at ~216 nm. To be more specific, when the AmH^+^‐Bpy^2+^‐Trz^+^ form is considered at TD‐B3LYP level, we estimate that the strongest singlet electronic transition falls at 258 nm with an oscillator strength (*f*) of 1.084; it mainly arises from a vertical HOMO‐15→LUMO (63%) excitation, clearly showing a ^1^π→π* character as reported in Figure 2a. The occupied Kohn–Sham (K‐S) molecular orbital actually results in widespread over both Bpy^2+^ and Trz^+^ moieties, while the LUMO orbital is seen to be localized over the Bpy^2+^ subunit. In analogy, the Am‐Bpy^2+^‐Trz^+^ form presents an electronic excitation

lying at 261 nm (*f* = 0.732), which still characterized as ^1^π→π* being associated with a HOMO‐17→LUMO (59%), see Figure 2b. The second adsorption band estimated at ~216 nm results, in the case of the AmH^+^‐Bpy^2+^‐Trz^+^, from the overlap of two ^1^π→π* electronic excitations computed at 218 (*f* = 0.168) and 215 (*f* = 0.237) nm, the excitation at 218 nm being dominated by a HOMO‐8→LUMO (46%) contribution is accompanied by a charge transfer (CT) process from the Trz^+^ aromatic ring to the antibonding aromatic counterpart of the Bpy^2+^. On the other hand, the transition detected at 215 nm results confined within the left side aromatic moiety of the investigated thread, mainly featuring a HOMO‐5→LUMO+5 (64%) excitation (see Figure 2a). Furthermore, we can observe that, within the same interval, the Am‐Bpy^2+^‐Trz^+^ species presents essentially the same trend with a ^1^π→π* valence excitation always arising from a HOMO‐5→LUMO+5 (54%) and falling at 217 nm (*f* = 0.183). At last, for the sake of completeness, we have to mention that a relatively small absorption band at ~346 nm is also observed. Such a band arises from the overlap of two ^1^π→π* electronic transitions close in energy at 353 (*f* = 0.034) and 341 (*f* = 0.043) nm, featuring a HOMO‐5→LUMO (98%) and HOMO‐1→LUMO (93%) character, respectively (see Figure 2a). The CAM‐B3LYP computations do not reveal such a small band, and the intensity of the two main adsorptions appears to be inverted (see also Tables S1 and S2). Looking at these data, it seems that, for the same character in the singlet electronic transitions observed, the B3LYP tends to underestimate the oscillator strength with respect to its CAM formulation. As already mentioned, the selectively reduced form of the investigated thread (i.e., Am‐Bpy‐Trz^+^) displays an adsorption band not observed in the other forms [B3LYP at ~375 nm (*f=*1.386); CAM‐B3LYP at ~361 nm (*f=*1.577), see data collected in Table S3.a and S3.b]. This is due to the fact that, following the selective electrochemical reduction, the two subunits constituting the Bpy group assume a planar conformation enabling it to act as a bridging ligand in coordination chemistry and a precursor for herbicides like paraquat [56, 57, 58, 59]. On the other hand, the two six‐membered rings are found to reside over two different planes when the oxidized form is present (Bpy^2+^). Bipyridinium structures are generally not planar, and their deviation from planarity can be described by factors like “bow” and “twist” deformations, leading to a nonplanar arrangement [60, 61]. The degree of planarity depends on the specific molecular structure, including substituents and the surrounding environment and can range from slightly twisted to significantly nonplanar in crystal lattices or metal complexes. In this specific case, we observe that the two six‐membered rings of the Bpy^2+^ assume a twisted conformation with a dihedral angle of 47° between them. As a result, such an orthogonal reduction is expected to finely tune the conformational geometry within the target Bpy unit as well as to modulate the adsorption of light as detected within the imposed TD‐DFT computational scenario. Indeed, the adsorption band that appears in Figure 1 at the longest wavelengths (~375 nm at TD‐B3LYP and ~361 nm at TD‐CAM‐B3LYP) in the presence of the Am‐Bpy‐Trz^+^ species mainly involves the HOMO orbital confined within a delocalized π electronic density over the two aromatic rings of the Bpy unit itself. More specifically, such a band presents a HOMO→LUMO+7 (57%) and HOMO→LUMO + 6 (30%) composition; these molecular orbitals may be appreciated in Figure 2c at B3LYP/6‐311G** level. It therefore appears that such a UV‐Vis adsorption has a ^1^π→π* character also involving an unoccupied molecular orbital widespread over the left half of the Am‐Bpy‐Trz^+^ system. Other ^1^π→π* electronic transitions—albeit to a lesser extent—are seen to be responsible for the adsorption of light at around 220 nm. The complete list of the molecular orbitals characterizing these adsorptions is reported in Table S3.a (B3LYP) and Table S3.b (CAM‐B3LYP), and the orbitals associated with the most intense transitions are displayed in Figure 2c.

**FIGURE 3:**
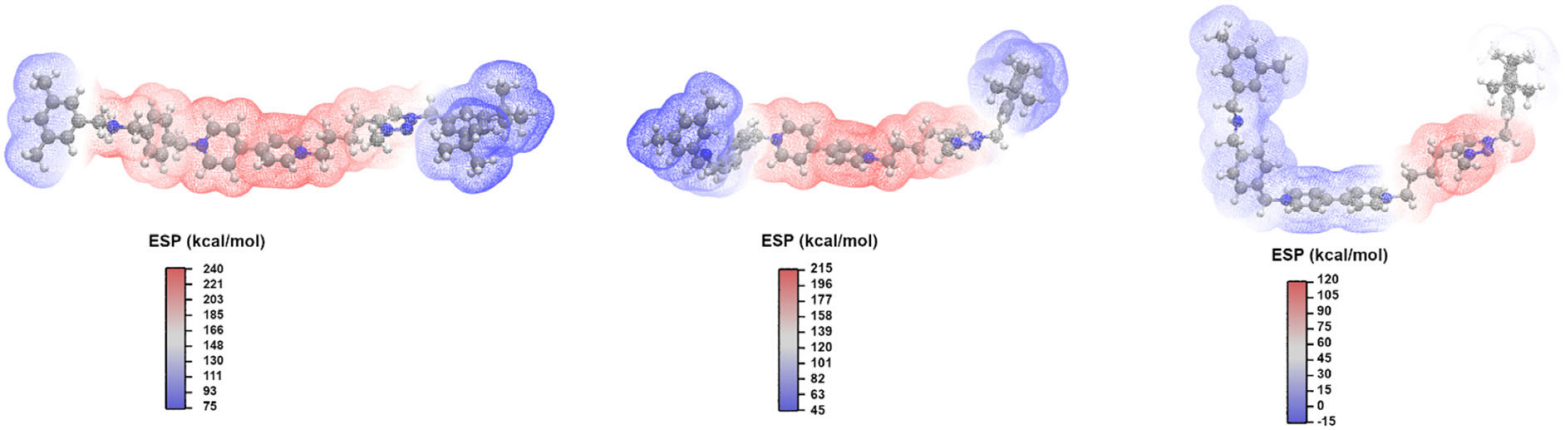
ESP (in kcal/mol) colored electron density isosurface on the vdW surface (*ρ* = 0.001 *a*_0_·*e* ^−3^) of the AmH^+^‐Bpy^2+^‐Trz^+^(left panel), Am‐Bpy^2+^‐Trz^+^ (middle panel), and Am‐Bpy‐Trz^+^ (right panel) forms of the investigated molecular thread at C‐PCM(CH_2_Cl_2_)/B3LYP(D3)/6‐311G** level of computations.

**FIGURE 4:**
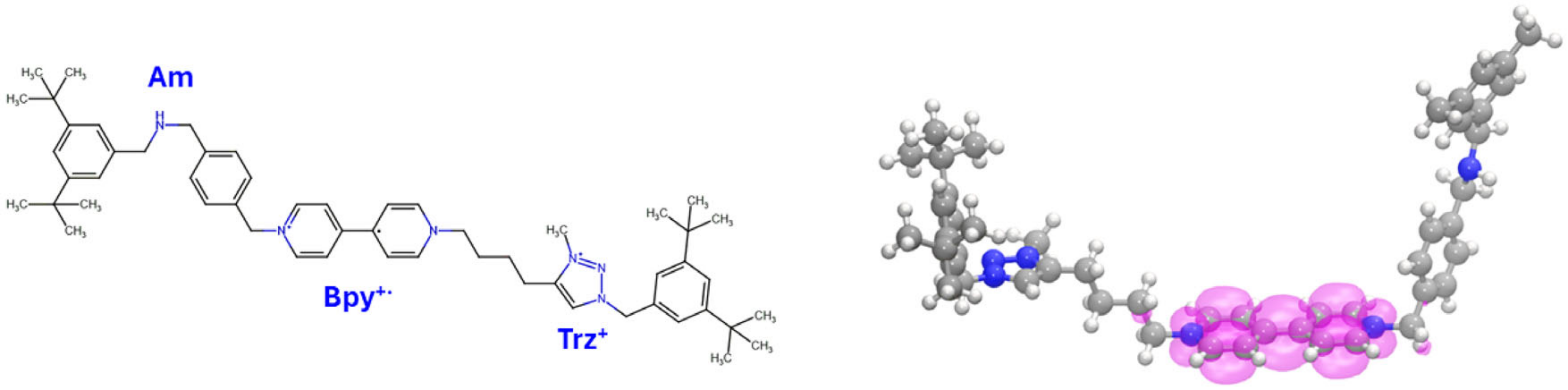
Left panel: molecular structure of the Am‐Bpy^·+^‐Trz^+^ radical form [31]; right panel: C‐PCM(CH_2_Cl_2_)/B3LYP(D3)/6‐311G** optimized molecular geometry and the estimated *α*(e)‐*β*(e) spatial electronic spin density isosurface (magenta color, isovalue equal to 0.015 *e*·*a*_0_^−3^).

As a next step, we focused our attention on a quantitative analysis of the ESP surfaces of AmH^+^‐Bpy^2+^‐Trz^+^, Am‐Bpy^2+^‐Trz^+^, and Am‐Bpy‐Trz^+^ species in CH_2_Cl_2_ dilute solution. These ESP surfaces reflect the true electrostatic nature of the molecular wires studied; this physical property can therefore be correlated with the different affinities in the macrocycle recognition pathways recently observed [31]. In this respect, the ESP colored van der Waals (vdW) surface maps of the three investigated systems are reported in Figure 3. Looking at this figure, we can realize that the AmH^+^‐Bpy^2+^‐Trz^+^ form shows—as it would have been expected—the most prominent positive values of the molecular electrostatic potential, reaching value up to 240 kcal/mol, in proximity of the AmH^+^ moiety, which actually makes this position prone to establish an intramolecular H‐bond contact with electron‐rich oxygen ether atoms within the **DB24C8** macrocycle. Also, we can observe a positive potential map that encompasses all stations with a positive charge. After that, it is known [31] that the deprotonation of AmH^+^ causes the ring translation toward the central station Bpy^2+^; this experimentally observed trend is supported by our C‐PCM/DFT‐based results. As can be clearly observed in Figure 3 (middle panel), the deprotonation of the primary recognition site is accompanied by a local attenuation of the electrostatic potential, while the Bpy^2+^‐Trz^+^ segment remains largely positive and is capable of attracting the ether‐crown macrocycle. In this respect, the repertoire of the highest positive ESP values resides over the VdW surface of the Bpy^2+^ moiety. At last, we can appreciate that when the Am‐Bpy‐Trz^+^ species is concerned, the only positive values of the ESP are confined within the Trz^+^ chemical group to justify its attitude to drive the shuttling mechanism of the ether ring from neutral Bpy in CH_2_Cl_2_ solution environment [31]. As a final step, we also investigated the monoreduced radical form (e.g., Am‐Bpy^·+^‐Trz^+^) proposed in literature [31], showing in Figure 4 the relaxed structure at C‐PCM/DFT level and the spatial electronic spin density showing the position of the unpaired *α*‐electron widespread over the Bpy moiety only.

### 3.2 Host‐guest Supramolecular Interaction Patterns

**FIGURE 5:**
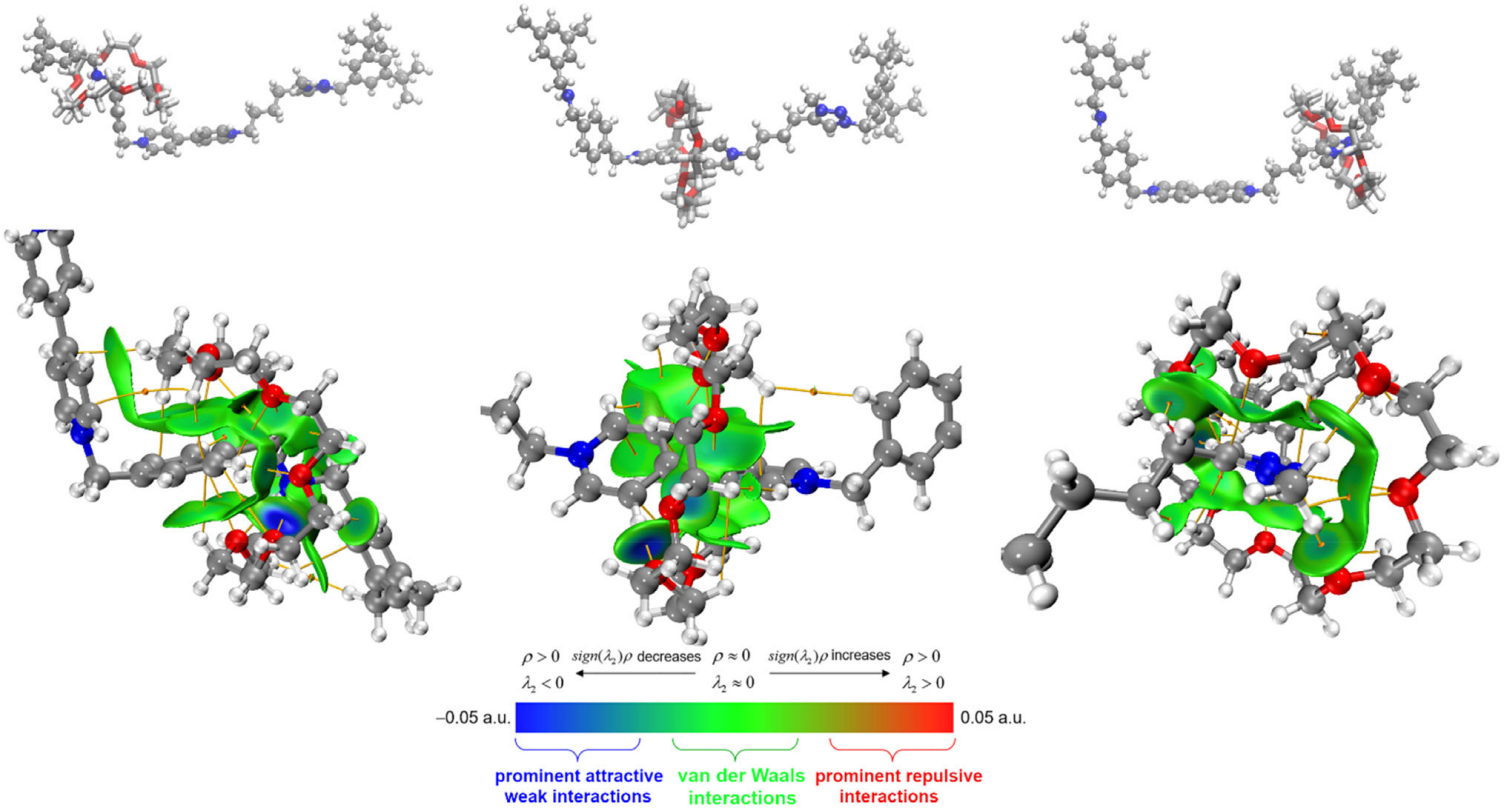
Upper panel: optimized geometry—at C‐PCM(CH_2_Cl_2_)/B3LYP(D3)/6‐311G** level—of the AmH^+^[**DB24C8**]‐Bpy^2+^‐Trz^+^(left panel), Am‐Bpy^2+^[**DB24C8**]‐Trz^+^ (middle panel), and Am‐Bpy‐Trz^+^[**DB24C8**] (right panel). Lower panel: local views of *sign*(*λ*_2_)*ρ* colored isosurfaces (δg^inter^ = 0.005 a.u., grid spacing 0.10 *a*_0_) corresponding to IGMH analysis. The QTAIM BPs and the associated (3,–1) BCPs are also reported.

The optimized molecular structure—at C‐PCM(CH_2_Cl_2_)/B3LYP(D3)/6‐311G** level of theory—of the investigated [2]rotaxane in CH_2_Cl_2_ are displayed in Figure 5 (upper panel): AmH^+^[**DB24C8**]‐Bpy^2+^‐Trz^+^(left panel), Am‐Bpy^2+^[**DB24C8**]‐Trz^+^ (middle panel) and Am‐Bpy‐Trz^+^[**DB24C8**] (right panel). In addition, a combined QTAIM and IGMH local analysis (see Subsection 2.0) in proximity of the host(**DB24C8**)/guest(AmH^+^; Bpy^2+^; Trz^+^) interacting moieties is also shown in the lower panel of the same figure. Looking at this picture, the supramolecular interaction patterns modulating the discretionary affinity recently observed [31] may be appreciated. In particular, and in line with studies over similar systems [23, 24, 62, 63], the AmH^+^[**DB24C8**] interacting species are held together mainly by H‐bonding contacts of electrostatic nature as clearly revealed by IGMH isosurface (Figure 5, left lower panel) and the associated QTAIM descriptors in Table 1. The region between the molecular thread and the ether ring includes numerous *ρ*(***r***) bond critical points (BCPs) of the type (+3,−1) [33, 34, 35, 64]. The strongest supramolecular interactions are represented by a couple of N‐H ‐‐ O_et_ contacts connecting the AmH^+^ unit with the oxygen ether atoms of the surrounding **DB24C8** macrocycle. More in details, we estimated, at the associated BCP, a *ρ*(***r***)*≈* 0.047 and 0.042 *e*·*a*_0_^−3^ with interatomic distances lying at 1.68 and 1.76 Å, respectively, the $\nabla^{2}$ *ρ*(***r***) values result positive, while the *H*(***r***) terms are found to be small and negative (−0.0045 and −0.0021 hartree·*a*_0_^−3^) and the *–G*(***r***)*/V*(***r***) ratio slightly lower than one (see Table 1). The existence of intermolecular or intramolecular H‐bonds may be analyzed efficiently within the QTAIM framework [33, 34, 35, 62, 63, 65, 66, 67]; in particular, when $\nabla^{2}$ *ρ*(***r***_***cp***_) and *H*(***r***_***cp***_) terms are both positive, the H‐bond is classified as weak and purely electrostatic. On the other hand, when these descriptors are both negative, the H‐bond results are particularly strong with a covalent character. In comparison, a positive $\nabla^{2}$ *ρ*(***r***_***cp***_) coupled with a negative *H*(***r***_***cp***_) characterizes a medium‐strong H‐bond with a partially covalent nature. The blue IGMH surfaces also clearly indicate this subset of prominent interactions that can be appreciated between the AmH^+^ group and the ether oxygen atoms in the ring (see Figure 5, lower panel). Always remaining in this context, the underlying binding energies (BEs) are estimated from QTAIM analysis by using—for charged interacting groups—a linear equation depending on *ρ*(***r***_***cp***_) as proposed in literature by T. Lu and coworkers: B_E_(kcal/mol) = −332.34 × *ρ*(***r***_***cp***_) −1.0661 [68]. In doing so, for the derived N‐H ‐‐ O_et_ interactions listed in Table 1, we can estimate B_E_ values of −16.9_5_, −14.9_6,_ and −5.4_2_ kcal/mol, respectively. We are then referring to two medium‐strong H‐bond contacts with the addition of a further weak N‐H ‐‐ O_et_ component at 2.28 Å [*ρ*(***r***)*=* 0.0131 *e*·*a*_0_^−3^, $\nabla^{2}$ *ρ*(***r***)* = 0.0477 e*·*a*_0_^−5^, and *H*(***r***) = 0.0015 hartree·*a*_0_^−3^]. In addition, our analysis reveals that further noncovalent forces of viable nature contribute to stabilizing the mutual position of the **DB24C8** ring in proximity to the AmH^+^ station; we estimate *ρ*(***r***_***cp***_) values within 0.0196 *e*·*a*_0_^−3^, the $\nabla^{2}$ *ρ*(***r***_***cp***_) and the *H*(***r***_***cp***_) are systematically positive, and the *‐G*(***r***)*/V*(***r***) ratios result greater than one witnessing that these interactions are actually controlled by a local excess in the kinetic energy. We are relying on weak vdW interactions of different nature: 6 BPs connecting C(sp^2^) ‐‐ H‐C(sp^3^) interatomic species, 2 BPs with a C(sp^2^) ‐‐ O_et_ composition, a single C(sp^2^)‐H ‐‐ H‐C(sp^3^) contact, 5 BPs connecting C(sp^2^)‐H ‐‐ O_et_ moieties, and 4 BPs between C(sp^3^)‐H ‐‐ O_et_.

**TABLE 1:** Electron density *ρ*(***r***) (*e*·*a*_0_^−3^), Laplacian of electron density ∇^2^ *ρ*(***r***) (*e*·*a*_0_^−5^), electron kinetic energy density *G*(***r***) (hartree·*a*_0_^−3^), electron potential energy density *V*(***r***) (hartree·*a*_0_^−3^), and electron energy density *H*(**r**) (hartree·*a*_0_^−3^) for bond critical points on selected bonds of the AmH^+^[**DB24C8**]‐Bpy^2+^‐Trz^+^ molecular shuttle calculated at C‐PCM(CH_2_Cl_2_)/B3LYP(D3)/6‐311G** level of theory.

BCP	Distance in Å	*ρ*(***r***)	∇^2^ *ρ*(***r***)	*G*(***r***)	*V*(***r***)	*‐ G*(***r***)/ *V*(***r***)	*H*(**r**)
C(sp^2^) ‐‐ H‐C(sp^3^)	2.48	0.0124	0.0393	0.0081	−0.0064	1.2646	0.0017
C(sp^2^) ‐‐ H‐C(sp^3^)	2.52	0.0105	0.0301	0.0064	−0.0052	1.2235	0.0012
C(sp^2^) ‐‐ H‐C(sp^3^)	2.61	0.0093	0.0265	0.0055	−0.0043	1.2688	0.0012
C(sp^2^) ‐‐ H‐C(sp^3^)	2.65	0.0086	0.0247	0.0052	−0.0042	1.2423	0.0010
C(sp^2^) ‐‐ H‐C(sp^3^)	2.67	0.0087	0.0270	0.0056	−0.0044	1.2718	0.0012
C(sp^2^) ‐‐ H‐C(sp^3^)	3.4	0.0023	0.0068	0.0013	−0.0009	1.3933	0.0004
C(sp^2^) ‐‐ O_et_	2.94	0.0112	0.0407	0.0088	−0.0074	1.1882	0.0014
C(sp^2^) ‐‐ O_et_	3.12	0.0081	0.0277	0.0059	−0.0049	1.2004	0.0010
C(sp^2^)‐H ‐‐ H‐C(sp^3^)	1.74	0.0179	0.0542	0.0115	−0.0094	1.2250	0.0021
C(sp^2^)‐H ‐‐ O_et_	2.15	0.0196	0.0683	0.0151	−0.0132	1.1465	0.0019
C(sp^2^)‐H ‐‐ O_et_	2.25	0.0143	0.0487	0.0105	−0.0088	1.1885	0.0017
C(sp^2^)‐H ‐‐ O_et_	2.49	0.0102	0.0356	0.0077	−0.0065	1.1877	0.0012
C(sp^2^)‐H ‐‐ O_et_	2.53	0.0082	0.0269	0.0058	−0.0049	1.1810	0.0009
C(sp^2^)‐H ‐‐ O_et_	3.09	0.0082	0.0277	0.0059	−0.0049	1.2040	0.0010
C(sp^3^)‐H ‐‐ H‐C(sp^3^)	2.03	0.0094	0.0273	0.0057	−0.0045	1.2504	0.0011
C(sp^3^)‐H ‐‐ H‐C(sp^3^)	2.29	0.0058	0.0156	0.0034	−0.0028	1.1836	0.0005
C(sp^3^)‐H ‐‐ H‐C(sp^3^)	2.44	0.0050	0.0165	0.0034	−0.0027	1.2756	0.0007
C(sp^3^)‐H ‐‐ H‐C(sp^3^)	2.68	0.0028	0.0087	0.0018	−0.0014	1.3060	0.0004
C(sp^3^)‐H ‐‐ O_et_	2.18	0.0177	0.0586	0.0130	−0.0114	1.1435	0.0016
C(sp^3^)‐H ‐‐ O_et_	2.36	0.0118	0.0381	0.0083	−0.0071	1.1671	0.0012
C(sp^3^)‐H ‐‐ O_et_	2.63	0.0073	0.0276	0.0059	−0.0048	1.2179	0.0010
C(sp^3^)‐H ‐‐ O_et_	2.78	0.0064	0.0261	0.0054	−0.0044	1.2461	0.0011
N‐H ‐‐ O_et_	1.68	0.0478	0.1488	0.0417	−0.0462	0.9028	−0.0045
N‐H ‐‐ O_et_	1.76	0.0418	0.1347	0.0357	−0.0378	0.9455	−0.0021
N‐H ‐‐ O_et_	2.28	0.0131	0.0477	0.0104	−0.0089	1.1676	0.0015

In turn, *sign*(*λ*_2_)*ρ* colored δ*g* ^inter^ IGMH isosurface map—computed considering the functionalized thread and the mechanically interlocked macrocycle as two separated fragments—displayed in Figure 5, left panel, allows a better graphical effect of the aforementioned intermolecular interactions between the Am‐Bpy‐Trz^+^ thread and the **DB24C8** macrocycle (treated as separated fragments). The interactions are revealed by the isosurfaces of δ*g* ^inter^ = 0.005 a.u., which are colored by *sign*(*λ*_2_) according to the color bar.

QTAIM topological analysis with the support of the IGMH visualization is also used for investigating the Am‐Bpy^2+^[**DB24C8**]‐Trz^+^ adduct in CH_2_Cl_2_ solution; the results that have emerged are collected in Table S4 and can be contextually appreciated in Figure 5, middle panel. In this respect, as recently appreciated over a similar [2]rotaxane shuttle [69], the supramolecular patterns stabilizing the observed host [**DB24C8**]/guest[Bpy^2+^] complexation [31] are mainly composed by a sort of electrostatic glue in which C(sp^2^)‐H groups of the Bpy^2+^ interact noncovalently with the oxygen atoms in the crown‐ether ring, forming weak vdW interactions referred to as C–H···O H‐bonds [70]. The related BPs feature *ρ*(***r***_***cp***_) spanning a region between 0.0336 and 0.0061 *e*·*a*_0_^−3^ with the largest C‐PCM/B3LYP(D3) density at the mutual distance of 1.84 Å; the associated B_E_ was estimated in the range between −12.2_3_ and −3.0_9_ kcal/mol [68]. The same contacts were also observed for the AmH^+^[**DB24C8**]‐Bpy^2+^‐Trz^+^ aggregate with the involvement of the aromatic ring between the AmH^+^ and the Bpy^2+^ moieties (see Table 1). We would also like to point out that: *i*) 4 BPs of the C(sp^2^) ‐‐ H‐C(sp^3^) type are estimated within the level of theory applied with *ρ*(***r***_***cp***_) within 0.0061 *e*·*a*_0_^−3^, the related $\nabla^{2}$ *ρ*(***r***_***cp***_) and *H*(***r***_***cp***_) values are systematically positive, and the *‐G*(***r***)*/V*(***r***) ratio greater than one; *ii*) 6 BPs with a C(sp^2^) ‐‐ O_et_ composition are emerged characterizing weak noncovalent contacts with a maximum *ρ*(***r***_***cp***_) of *ca*. 0.014 *e*·*a*_0_^−3^; *iii*) two very weak C(sp^2^)‐H ‐‐ H‐C(sp^3^) intermolecular interactions are also appreciated with distances at 2.62 and 2.74 Å and *ρ*(***r***_***cp***_) < 0.003 *e*·*a*_0_^−3^. At last, the same analysis was proposed over the Am‐Bpy‐Trz^+^[**DB24C8**] system. As can be seen in Table S5 and Figure 5 (right panel), we can assume that the ring studied—in the obtained C‐PCM(CH_2_Cl_2_)/B3LYP(D3) relaxed structure depicted in Figure 5 (upper panel)—presents a complex reservoir of noncovalent interactions involving different chemical fragments. The strongest interaction computed features a C(sp^2^)‐H ‐‐ O_et_ contact with a BCP showing a *ρ*(***r***_***cp***_) of 0.0275 *e*·*a*_0_^−3^ and a $\nabla^{2}$ *ρ*(***r***_***cp***_) of *ca*. 0.1 *e*·*a*_0_^−5^ at a distance of 1.94 Å (B_E_= ‐ 10.2_1_ kcal/mol) [68]; such an electrostatic contact involves the H atom connected with the aromatic Trz^+^ 5‐membered ring. Subsequently, a subset of 6 C(sp^3^)‐H ‐‐ O_et_ BPs are estimated, showing that ether oxygen's lone pair can effectively interact with aliphatic hydrocarbons, contributing to stabilize the Am‐Bpy‐Trz^+^[**DB24C8**] supramolecular assembly in CH_2_Cl_2_ solution. Interatomic C(sp^3^)‐H ‐‐ O_et_ distances are found in the range between 2.08–2.73 Å with *ρ*(***r***_***cp***_) showing the maximum value, 0.0209 *e*·*a*_0_^−3^, at the shortest distance. Interestingly, we also observe 3 C(sp^2^) ‐‐ O_et_ contacts directly, making contact with the carbon atoms within the 5‐membered ring of the Trz^+^ station. A deeper analysis reveals that these noncovalent interactions mainly arise from stabilizing electrostatic contacts between positive—the two C atoms with a charge of *ca*. 0.45*e—*and negative—O_et_ atoms with a charge of *ca.* −0.97*e—*domains employing the Atomic Dipole Corrected Hirshfeld atomic charge (ADCH) method [71] in Multiwfn program [49, 50]. We can also appreciate a subset of N(sp^2^) ‐‐ O_et_ supramolecular contacts involving N atoms of the 5‐membered ring with *ρ*(***r***_***cp***_) within *≈* 0.0134 *e*·*a*_0_^−3^. Other contacts, albeit to a lesser extent, are found between C(sp^2^) ‐‐ H‐C(sp^3^), C(sp^2^)‐H ‐‐ H‐C(sp^3^), and N_c_(sp^2^) ‐‐ H‐C(sp^3^) interacting couples. In any case, given the associated value of the $\nabla^{2}$ *ρ*(***r***_***cp***_), *‐G*(***r***)*/V*(***r***) and *H*(***r***_***cp***_), it is evident that the weak nature of the underlying mutual vdW interactions. The same picture may be obtained looking at the associated *sign*(*λ*_2_)*ρ* colored δ*g* ^inter^ IGMH isosurface map displayed in Figure 5 (lower right panel) when overimposed with respect to the QTAIM analysis collected in Table S5. As a conclusive remark, given the complexity of the investigated systems, we have reported, as Electronic Supporting Information, three different videos showing the results of the combined QTAIM + IGMH analysis over the supramolecular assembly species investigated in Figure 5.

In summary, the derived picture at C‐PCM(CH_2_Cl_2_)/B3LYP(D3)/6‐311G** level of theory shed some light on the intriguing and subtle reservoir of supramolecular interaction patterns playing a key role in finely tuning the discretional ring(**DB24C8**)/station(AmH^+^; Bpy^2+^; Trz^+^) affinity order observed via ^1^H NMR measurements in CH_2_Cl_2_ at 298K [31]. The fact of combining C‐PCM/DFT converged eigenvectors with QTAIM descriptors and a state‐of‐the‐art self‐consistent visualization method of supramolecular interactions in complex molecular systems actually paves the route toward novel design strategies for more efficient and sustainable molecular machines and devices operating in different contexts. The theoretical picture that has emerged, with the due approximations (medium solvation effects and the absence of thermal effects), results in nonconclusive and will be further revisited with a joint DFT/[QTAIM + IGMH] and Classical Molecular Dynamics (MD) technique to extract the thermodynamics driving forces modulating conformational shaping in solution at finite room‐temperature condition.

## 4 Conclusion

In this research contribution, we propose, for the first time, a self‐consistent theoretical investigation based on C‐PCM/DFT converged electronic wavefunctions on a very recently proposed tristable [3]rotaxane working in dichloromethane dilute solution as a result of selective (orthogonal) deprotonation and electrochemical stimuli. The derived scenario presents to the readers a first QM viewpoint of such an intriguing synthetic system in three different eigenstates [e.g., AmH^+^(**DB24C8**)‐Bpy^2+^‐Trz^+^, Am‐Bpy^2+^(**DB24C8)**‐Trz^+^, and Am‐Bpy‐Trz^+^(**DB24C8)**] mimicking the observations derived in the laboratory on the basis of state‐of‐the‐art spectroscopical measurements. Supramolecular interaction patterns at play connecting the mechanically interlocked macrocycle over the imposed sites are characterized by analytically combining QTAIM analysis and IGMH plot; despite the complexity of the systems investigated and the approximations made—single structures relaxed at C‐PCM/DFT level—the derived noncovalent contacts under equilibrium conditions in CH_2_Cl_2_ solution result in line with those revealed via ^1^H NMR measurement at 500 MHz and 298K. The proposed computational scenario, also supported by ESP calculations on the molecular thread alone, highlights the potential that simulation studies can offer in the rational design of soft molecular machines and devices of increasing complexity and functionalities. That said, it would be really interesting to consider involving theory when planning laboratory synthesis activities on different elements constituting a molecular machine to be proposed in literature. This is also with a view to moving toward more eco‐sustainable syntheses capable of reducing the environmental impact.

## Supporting Information

Additional supporting information can be found online in the Supporting Information section. Relaxed coordinates (XYZ format) of AmH^+^(**DB24C8**)‐Bpy^2+^‐Trz^+^, Am‐Bpy^2+^(**DB24C8)**‐Trz^+^ and Am‐Bpy‐Trz^+^(**DB24C8)** systems at C‐PCM(CH_2_Cl_2_)/B3LYP(D3)/6‐311G** level of theory; three videos showing the results of the proposed QTAIM+IGMH analysis; vertical excitation energies and related composition at C‐PCM(CH_2_Cl_2_)/TD‐B3LYP(D3)/6‐311G** and C‐PCM(CH_2_Cl_2_)/TD‐CAM‐B3LYP(D3)/6‐311G** level. QTAIM Tables for both Am‐Bpy^2+^(**DB24C8)**‐Trz^+^ and Am‐Bpy‐Trz^+^(**DB24C8)** systems.

## Conflicts of Interest

The authors declare no conflicts of interest.

## Supporting information

Supplementary Material

## Data Availability

The data that supports the findings of this study are available in the supplementary material of this article.
