# Supplementary material for: A Density Functional Theory‐Based Investigation of a pH‐ and Redox‐Driven Tristable [2]Rotaxane in CH2Cl2 Dilute Solution
Source: ChemistryOpen. 2026 Apr 23;15(5):e70207. doi: 10.1002/open.70207 (PMC13105323; doi:10.1002/open.70207)
Supplement: Supplementary file 1 — Supplementary Material [file OPEN-15-e70207-s001.pdf]

A DFT based Investigation of a pH- and Redox-Driven Tristable [2]Rotaxane in CH_2Cl_2 Dilute Solution

Costantino Zazza,*[a] Nico Sanna,[a] [b] Stefano Borocci,[a] [c] and Felice Grandinetti [a] [c]

[a] Dr. Costantino Zazza, Prof. Nico Sanna, Prof. Stefano Borocci, and Prof. Felice Grandinetti,
Department for Innovation in Biological, Agro-food and Forest systems, Università della Tuscia (DIBAF), L.go dell'Università, s.n.c., 01100 Viterbo, Italy.
E-mail: costantino.zazza@unitus.it

[b] Prof. Nico Sanna,
Istituto per la Scienza e Tecnologia dei Plasmi del CNR (ISTP), Via G. Amendola 122/D, 70126 Bari, Italy.

[c] Prof. Stefano Borocci, Prof. Felice Grandinetti,
Istituto per i Sistemi Biologici del CNR (ISB), Sede di Roma - Meccanismi di Reazione c/o Dipartimento di Chimica, Sapienza Università di Roma, P.le A. Moro 5, 00185, Rome, Italy.

Electronic Supporting Information

IGMH basic equations and modelling:

IGMH method utilizes a Hirshfeld partitioning scheme to separate the molecular density into individual atomic contributions. The starting point for the IGMH method is the reduced electron density (Reduced Density Gradient, RDG), defined as:[51,52]

$$s(\boldsymbol{r}) = \frac{|\nabla\rho(\boldsymbol{r})|}{2(3\pi^2)^{\frac{1}{3}} \times \rho(\boldsymbol{r})^{\frac{4}{3}}} \qquad (8)$$

Low-value Reduced Density Gradient (RDG) isosurfaces, often used in analyzing Non-Covalent Interactions (NCIs), are characterized by relatively low values of *s(r)*, typically between 0.3 and 0.6.[53-57] More specifically the Hirshfeld partitioning scheme applied defines a weighted atomic density:

$$\rho_i^{Hirsh}(\boldsymbol{r}) = \rho(\boldsymbol{r}) \cdot w_i(\boldsymbol{r}) \qquad (9)$$

where $\rho(\boldsymbol{r})$ is the actual electron density calculated by quantum chemistry method while the Hirshfeld weighting function of the atom *i* is defined as:

$$w_i(\boldsymbol{r}) = \frac{\rho_i^0\ (\boldsymbol{r})}{\rho^0\ \ (\boldsymbol{r})} = \frac{\rho_i^0\ (\boldsymbol{r})}{\sum_j^{Natoms}\ \ \rho_j^0\ (\boldsymbol{r})} \qquad (10)$$

where index *j* loops over all atoms, and the $\rho^0(\boldsymbol{r})$ in the denominator is a promolecular density (also referred as reference pro-atom densities) and ρ_i^0 stands for spherically averaged density around atom *i* in its unperturbed (free) state. In the IGM model the $\rho^0(\boldsymbol{r})$ is actually defined purely on densities of atoms in their free states under the so-called promolecular approximation.[46] On the contrary, in the IGMH method,[47,48] the free-state atomic density (*e.g.,* ρ_i^0) in the IGM description is replaced with ρ_i^{Hirsh}. The key descriptor of IGM-based models is the δg^{inter} function, which isolates intermolecular (or interfragment) interactions:

$$\delta g^{inter} = |\ \nabla\rho^{IGM}(\boldsymbol{r})\ | - |\ \nabla\rho(\boldsymbol{r})\ | \qquad (11)$$

where $\rho^{IGM}(\boldsymbol{r})$ is the electron density constructed as the sum of the densities of isolated promolecular fragments:

$$\rho^{IGM}(\boldsymbol{r}) = \sum_{fragments} \rho(\boldsymbol{r})_{fragments} \qquad (12)$$

WILEY-VCH

In the Hirshfeld-based version, the function is modified to account - starting from converged electronic wavefunction - for atomic partitioning as follows:

$$\delta g_{Hirsh}^{inter} = \sum_{i\epsilon frag1} \sum_{j\epsilon frag2} |\nabla\rho_i(\boldsymbol{r})| + |\nabla\rho_j(\boldsymbol{r})| - |\nabla\left(\rho_i(\boldsymbol{r}) + \rho_j(\boldsymbol{r})\right)| \quad (13)$$

The principle is brilliant in its simplicity; if there were no interaction between *i* (fragment 1) and *j* (fragment 2) couple of atoms, the additive property of gradients would apply:

$$g_{i,j}(\boldsymbol{r}) = |\nabla\left(\rho_i(\boldsymbol{r}) + \rho_i(\boldsymbol{r})\right)| = |\nabla\rho_i(\boldsymbol{r})| + |\nabla\rho_j(\boldsymbol{r})| = g_{i,j}^{IGM}(\boldsymbol{r}) \quad (14)$$

and therefore $\delta g_{Hirsh}^{inter} = 0$; when interaction occurs, the electron density is redistributed and:

$$|\nabla\left(\rho_i(\boldsymbol{r}) + \rho_j(\boldsymbol{r})\right)| \neq |\nabla\rho_i(\boldsymbol{r})| + |\nabla\rho_j(\boldsymbol{r})| \quad (15)$$

the difference measures how much the combined system deviates from the behavior of the two isolated, non-interacting, atoms. Specifically, in IGMH analysis, the ρ_i terms over isolated fragments in above expressions corresponds to ρ_i^{Hirsh}. In this context, the δg_{Hirsh}^{inter} related isosurfaces are then used to visually appreciate the interaction regions, while the function *sign(λ₂)ρ(r)* is overimposed on the estimated isosurfaces to visually characterize the nature of the chemical interactions.[47,48] The latter indicates the product of the electron density, *ρ*, and the sign of the second largest eigenvalue of the Hessian matrix of the electronic density; *ρ* in the interaction region is generally positively correlated with the interaction force, while *sign(λ_2)* has instead the ability to distinguish whether the interaction patterns are merely attractive or repulsive.[47,48]

references: the number of the references is that of the associated manuscript

Table S1.a: TD-DFT[C-PCM(CH_2Cl_2)/B3LYP(D3)/6-311G**] singlet electronic excitations for the investigated AmH^+-Bpy^{2+}-Trz^+ molecular thread. The derived UV/Vis absorption spectrum is shown in the associated manuscript in Fig 1 (upper left panel).

Wavelength (nm)	Osc, Strength	Major contribs	Minor contribs
515.9	0.000	HOMO->LUMO (100%)	
482.1	0.000	H-1->LUMO (100%)	
461.9	0.000	H-2->LUMO (100%)	
426.9	0.004	H-3->LUMO (99%)	
358.7	0.000	H-4->LUMO (99%)	
355.0	0.004	HOMO->L+2 (93%)	HOMO->L+3 (5%)
353.0	0.034	H-5->LUMO (98%)	
340.8	0.044	H-1->L+2 (93%)	H-1->L+3 (5%)
336.3	0.000	HOMO->L+1 (98%)	
321.7	0.000	H-1->L+1 (98%)	
315.1	0.000	HOMO->L+3 (86%), HOMO->L+4 (10%)	HOMO->L+2 (5%)
312.8	0.000	H-2->L+1 (100%)	
309.6	0.000	HOMO->L+4 (90%)	HOMO->L+3 (9%)
302.2	0.000	H-1->L+3 (85%), H-1->L+4 (10%)	H-1->L+2 (5%)
297.1	0.000	H-1->L+3 (10%), H-1->L+4 (90%)	
296.2	0.007	H-3->L+1 (99%)	
296.2	0.000	H-2->L+3 (90%)	H-2->L+2 (6%), H-2->L+4 (4%)
294.5	0.000	H-6->LUMO (99%)	
292.9	0.050	H-8->LUMO (97%)	
289.4	0.000	H-2->L+2 (94%)	H-2->L+3 (5%)

287.6	0.000	H-7->LUMO (99%)	
287.4	0.000	H-2->L+4 (95%)	H-2->L+3 (5%)
281.7	0.000	H-3->L+3 (88%)	H-3->L+2 (6%), H-3->L+4 (5%)
274.7	0.000	H-3->L+2 (94%)	H-3->L+3 (5%)
273.2	0.001	H-3->L+4 (93%)	H-3->L+3 (6%)
271.4	0.004	H-9->LUMO (99%)	
267.9	0.000	H-10->LUMO (100%)	
267.2	0.008	H-2->L+5 (94%)	H-3->L+9 (3%), H-2->L+7 (2%)
267.1	0.000	HOMO->L+5 (100%)	
265.1	0.067	H-13->LUMO (93%)	H-15->L+3 (2%)
263.1	0.028	H-14->LUMO (90%)	H-5->L+3 (2%)
260.7	0.001	H-4->L+1 (95%)	
258.5	0.007	H-11->LUMO (99%)	
258.2	1.084	H-15->LUMO (79%)	H-16->LUMO (9%), H-3->L+5 (7%)
257.7	0.000	H-1->L+5 (100%)	
256.9	0.005	H-5->L+1 (94%)	
253.0	0.017	H-4->L+3 (12%), H-3->L+5 (79%)	H-15->LUMO (5%)
252.6	0.039	H-4->L+3 (69%), H-4->L+4 (11%), H-3->L+5 (12%)	H-5->L+1 (2%), H-4->L+2 (4%)
251.2	0.002	H-5->L+3 (71%), H-5->L+4 (12%)	H-14->LUMO (3%), H-5->L+2 (4%), H-4->L+5 (4%)
247.0	0.000	H-12->LUMO (100%)	
242.1	0.011	H-1->L+8 (20%), H-1->L+10 (17%), H-1->L+11 (11%), HOMO->L+8 (16%), HOMO->L+10 (13%), HOMO->L+11 (23%)	
241.0	0.009	H-3->L+9 (27%), H-2->L+7 (67%)	H-2->L+5 (5%)
240.2	0.004	H-16->LUMO (88%), H-15->LUMO (10%)	
240.1	0.000	HOMO->L+6 (100%)	
239.2	0.004	H-4->L+4 (55%), H-4->L+5 (18%)	H-5->L+3 (3%), H-5->L+6 (9%), H-4->L+3 (7%), H-3->L+6 (5%)
237.9	0.000	H-2->L+6 (99%)	
237.6	0.004	H-5->L+6 (14%), H-4->L+4 (31%), H-4->L+5 (34%)	H-5->L+3 (4%), H-4->L+3 (4%), H-3->L+6 (9%)
236.4	0.000	H-17->LUMO (100%)	
235.2	0.003	H-5->L+3 (10%), H-5->L+4 (85%)	
235.0	0.000	H-4->L+2 (93%)	H-4->L+3 (6%)
233.9	0.008	H-1->L+8 (11%), HOMO->L+8 (79%)	HOMO->L+10 (4%), HOMO->L+11 (3%)
232.5	0.000	H-1->L+6 (100%)	
231.6	0.000	H-5->L+2 (93%)	H-5->L+3 (6%)
231.4	0.000	H-18->LUMO (100%)	
230.4	0.000	H-7->L+2 (13%), H-6->L+2 (77%)	H-6->L+1 (3%), H-6->L+3 (4%)
227.4	0.000	H-7->L+2 (76%), H-6->L+2 (12%)	H-7->L+1 (2%), H-7->L+3 (4%), H-6->L+1 (2%)
226.7	0.005	H-3->L+6 (85%)	H-5->L+6 (6%), H-4->L+5 (9%)
225.7	0.001	H-19->LUMO (95%)	H-6->L+1 (3%)
225.7	0.001	H-6->L+1 (90%)	H-19->LUMO (4%), H-6->L+2 (4%)

225.4	0.056	H-1->L+8 (61%), H-1->L+10 (15%)	H-8->L+1 (4%), H-1->L+11 (3%), HOMO->L+8 (2%), HOMO->L+10 (9%), HOMO->L+11 (3%)
224.9	0.008	H-8->L+1 (90%)	H-1->L+8 (3%)
224.1	0.003	H-20->LUMO (81%)	H-28->LUMO (2%), H-25->LUMO (3%), H-21->LUMO (7%)
223.0	0.000	H-21->LUMO (86%)	H-25->LUMO (3%), H-20->LUMO (8%)
222.0	0.000	HOMO->L+7 (100%)	
221.6	0.000	H-7->L+1 (96%)	H-7->L+2 (2%)
219.3	0.001	H-22->LUMO (93%)	H-20->LUMO (3%)
218.8	0.052	H-28->LUMO (38%), H-25->LUMO (16%), H-8->L+2 (13%)	H-23->LUMO (2%), H-15->L+1 (6%), H-5->L+5 (5%)
218.6	0.018	H-25->LUMO (63%)	H-28->LUMO (2%), H-26->LUMO (4%), H-23->LUMO (4%), H-21->LUMO (4%), H-20->LUMO (3%), H-8->L+2 (9%)
218.3	0.168	H-28->LUMO (10%), H-8->L+2 (46%)	H-23->LUMO (9%), H-15->L+1 (2%), H-9->L+2 (7%), H-5->L+5 (3%), HOMO->L+10 (4%), HOMO->L+11 (2%)
217.8	0.020	H-23->LUMO (79%)	H-28->LUMO (4%), H-25->LUMO (2%), H-5->L+5 (3%), H-3->L+7 (4%)
217.4	0.015	H-3->L+7 (51%), H-2->L+9 (30%)	H-28->LUMO (6%), H-23->LUMO (3%), H-15->L+1 (2%), H-5->L+5 (4%)
216.4	0.001	H-13->L+1 (16%), H-8->L+3 (35%), H-8->L+4 (35%)	H-15->L+4 (2%), H-14->L+1 (3%)
216.2	0.059	H-9->L+2 (20%), H-1->L+10 (18%), HOMO->L+10 (38%), HOMO->L+11 (10%)	H-8->L+2 (2%), H-1->L+11 (8%)
215.9	0.000	H-6->L+3 (87%)	H-6->L+2 (4%), H-6->L+4 (7%)
215.5	0.000	H-1->L+7 (100%)	
215.3	0.237	H-5->L+5 (64%), H-4->L+6 (12%)	H-28->LUMO (3%), H-15->L+1 (5%), H-3->L+7 (3%), H-2->L+9 (8%)
214.1	0.020	H-9->L+2 (56%), H-8->L+2 (17%)	H-9->L+1 (5%), H-9->L+3 (3%), H-1->L+10 (3%), HOMO->L+10 (7%), HOMO->L+11 (3%)
213.3	0.000	H-6->L+4 (93%)	H-6->L+3 (7%)
213.1	0.003	H-14->L+1 (10%), H-13->L+1 (37%), H-8->L+3 (32%)	H-15->L+4 (9%), H-8->L+4 (4%)
212.6	0.000	H-29->LUMO (89%)	H-30->LUMO (4%), H-27->LUMO (3%)
212.2	0.000	H-7->L+3 (84%)	H-14->L+1 (2%), H-7->L+2 (4%), H-7->L+4 (8%)
212.0	0.015	H-15->L+3 (12%), H-14->L+1 (49%), H-13->L+1 (11%), H-8->L+4 (13%)	H-9->L+1 (2%), H-8->L+3 (4%), H-7->L+3 (2%)
211.9	0.002	H-9->L+1 (91%)	H-9->L+2 (5%)
211.8	0.000	H-10->L+2 (84%)	H-10->L+1 (9%), H-10->L+3 (4%)
211.0	0.002	H-14->L+1 (12%), H-13->L+1 (14%), H-8->L+3 (22%), H-8->L+4 (44%)	H-15->L+4 (2%)
210.3	0.000	H-24->LUMO (99%)	
209.7	0.000	H-10->L+1 (15%), H-7->L+4 (75%)	H-7->L+3 (6%)

209.6	0.000	H-10->L+1 (76%), H-7->L+4 (15%)	H-10->L+2 (7%)
209.0	0.000	HOMO->L+9 (100%)	
208.1	0.000	H-2->L+8 (100%)	
206.5	0.006	H-28->LUMO (13%), H-15->L+1 (49%), H-14->L+3 (10%)	H-34->LUMO (2%), H-16->L+1 (6%), H-13->L+4 (8%)
206.2	0.001	H-32->LUMO (80%)	H-30->LUMO (9%), H-26->LUMO (4%)
205.5	0.001	H-11->L+1 (13%), H-11->L+2 (81%)	H-11->L+3 (4%)
204.7	0.000	H-27->LUMO (93%)	H-29->LUMO (3%), H-28->LUMO (4%)
204.5	0.000	H-26->LUMO (88%)	H-32->LUMO (5%), H-25->LUMO (6%)
203.8	0.000	H-11->L+1 (87%), H-11->L+2 (12%)	
203.3	0.000	H-9->L+3 (87%)	H-9->L+2 (4%), H-9->L+4 (8%)
203.2	0.000	H-1->L+9 (100%)	
201.5	0.006	H-30->LUMO (73%)	H-34->LUMO (4%), H-32->LUMO (9%), H-29->LUMO (4%), H-28->LUMO (3%)
201.3	0.000	H-10->L+3 (87%)	H-10->L+2 (5%), H-10->L+4 (6%)

Table S1.b: TD-DFT[C-PCM(CH_2Cl_2)/CAM-B3LYP(D3)/6-311G**] singlet electronic excitations for the investigated AmH^+-Bpy^{2+}-Trz^+ molecular thread. The derived UV/Vis absorption spectrum is shown in the associated manuscript in Fig 1 (lower left panel).

Wavelength (nm)	Osc, Strength	Major contribs	Minor contribs
271.2	0	HOMO->LUMO (99%)	
263.0	0.1602	H-5->LUMO (63%), H-3->LUMO (25%)	H-14->LUMO (2%), H-5->L+1 (7%)
261.4	0.0011	H-1->LUMO (98%)	
258.9	0.0001	H-2->LUMO (99%)	
255.2	0.0017	H-4->LUMO (78%)	H-13->LUMO (5%), H-12->LUMO (2%), H-4->L+1 (8%), H-4->L+5 (3%)
254.5	0.0017	HOMO->L+2 (15%), HOMO->L+3 (80%)	H-1->L+3 (3%)
254.4	0.0809	H-1->L+2 (14%), H-1->L+3 (77%)	HOMO->L+3 (3%)
246.4	0.1166	H-12->LUMO (78%)	H-14->L+2 (3%), H-14->L+4 (3%), H-13->L+1 (4%), H-12->L+1 (5%)
244.3	0.0527	H-13->LUMO (73%)	H-14->L+2 (2%), H-14->L+4 (3%), H-13->L+1 (4%), H-12->L+1 (4%), H-4->LUMO (6%)
243.5	0.1202	H-5->LUMO (18%), H-3->LUMO (70%)	H-14->LUMO (7%)
240.1	0.0211	H-3->L+9 (28%), H-2->L+5 (15%), H-2->L+7 (54%)	
237.4	0.8886	H-14->LUMO (79%)	H-7->LUMO (3%), H-5->LUMO (7%), H-3->LUMO (3%)
234.4	0.0068	H-1->L+8 (17%), H-1->L+10 (17%), H-1->L+11 (14%), HOMO->L+10 (17%), HOMO->L+11 (26%)	HOMO->L+8 (8%)
232.3	0.0055	H-5->L+6 (29%), H-4->L+5 (51%)	H-5->L+2 (3%), H-4->LUMO (8%), H-4->L+7 (3%)
216.1	0.1987	H-5->L+5 (11%), H-3->L+5 (28%), H-3->L+7 (33%), H-2->L+9 (17%)	H-4->L+6 (2%)
212.7	0.0573	H-1->L+10 (13%), H-1->L+11 (10%), HOMO->L+8 (28%), HOMO->L+10 (22%), HOMO->L+11 (15%)	H-1->L+8 (8%)
211.3	0.2303	H-7->L+2 (14%), H-7->L+3 (74%)	
210.0	0.1017	H-5->L+5 (41%), H-4->L+6 (14%), H-3->L+7 (12%), H-2->L+9 (15%)	H-5->L+1 (4%), H-4->L+2 (4%)
205.9	0.0003	HOMO->L+1 (99%)	

201.7	0.0056	H-26->LUMO (12%), H-7->LUMO (65%)	H-14->LUMO (2%), H-14->L+1 (2%), H-13->L+2 (3%), H-12->L+4 (2%), H-7->L+1 (2%), H-1->L+1 (3%)
200.2	0.0062	H-5->L+2 (29%), H-1->L+1 (39%)	H-5->L+3 (6%), H-5->L+4 (7%), H-4->L+5 (4%), H-3->L+2 (8%)
200.2	0.005	H-5->L+2 (19%), H-1->L+1 (57%)	H-5->L+3 (4%), H-5->L+4 (5%), H-4->L+5 (3%), H-3->L+2 (5%)
200.0	0.0113	H-2->L+1 (86%)	H-3->L+9 (3%), H-2->L+5 (9%)
198.8	0	HOMO->L+2 (44%), HOMO->L+4 (49%)	HOMO->L+3 (7%)
197.9	0.9329	H-4->L+2 (34%), H-2->L+9 (20%)	H-4->L+3 (7%), H-4->L+4 (9%), H-4->L+6 (7%), H-3->L+1 (7%), H-3->L+5 (9%)
196.3	0.0096	H-26->LUMO (21%), H-14->L+1 (15%), H-13->L+2 (11%), H-12->L+4 (14%), H-7->LUMO (20%)	H-38->LUMO (4%), H-13->L+3 (2%)
194.7	0	HOMO->L+2 (40%), HOMO->L+4 (51%)	HOMO->L+3 (9%)
194.6	0.0017	H-2->L+2 (72%), H-2->L+3 (14%), H-2->L+4 (11%)	
193.9	0.0521	H-5->L+5 (11%), H-4->L+2 (10%), H-3->L+1 (55%)	H-26->LUMO (3%), H-5->L+1 (7%), H-4->L+3 (2%), H-4->L+4 (2%)
193.5	0.0001	H-1->L+2 (44%), H-1->L+4 (50%)	H-1->L+3 (7%)
192.7	0.0038	H-21->L+3 (16%), H-19->L+3 (12%), H-8->L+3 (13%)	H-41->L+3 (5%), H-39->L+3 (6%), H-36->L+3 (9%), H-30->L+3 (4%), H-28->L+3 (2%), H-21->L+2 (3%), H-20->L+3 (6%), H-19->L+2 (2%), H-8->L+2 (2%), H-6->L+3 (5%)
192.6	0.263	H-5->L+1 (14%), H-4->L+2 (12%), H-3->L+5 (15%), H-2->L+9 (33%)	H-5->L+5 (3%), H-4->L+3 (2%), H-4->L+4 (3%), H-3->L+1 (5%), H-3->L+7 (7%)
192.4	0.2074	H-3->L+9 (31%), H-2->L+1 (12%), H-2->L+5 (53%)	
190.0	0.9766	H-1->L+8 (24%), H-1->L+10 (13%), HOMO->L+11 (51%)	H-16->L+3 (4%)
189.5	0.0034	H-1->L+2 (41%), H-1->L+4 (50%)	H-1->L+3 (9%)
188.9	0.0258	H-4->L+1 (70%)	H-13->LUMO (3%), H-13->L+1 (7%), H-12->L+1 (3%), H-5->L+6 (5%), H-4->LUMO (5%)
187.9	0.0564	H-26->LUMO (12%), H-14->L+1 (17%), H-5->L+1 (22%), H-3->L+1 (19%)	H-38->LUMO (3%), H-6->LUMO (5%), H-4->L+6 (2%), H-3->L+7 (3%), H-2->L+9 (2%)
187.8	0.0029	H-6->LUMO (93%)	
187.5	0.6161	H-1->L+11 (63%), HOMO->L+8 (14%)	H-16->L+3 (6%), H-1->L+10 (3%), HOMO->L+10 (6%)
186.6	0.0092	H-14->L+4 (20%), H-12->LUMO (11%), H-12->L+1 (40%)	H-14->L+2 (2%), H-13->LUMO (5%), H-13->L+1 (5%), H-3->L+2 (5%)
186.5	0.036	H-2->L+2 (10%), H-2->L+4 (83%)	H-3->L+9 (3%), H-2->L+7 (2%)
186.2	0.0048	H-3->L+2 (53%), H-3->L+3 (10%)	H-14->L+2 (3%), H-13->L+1 (5%), H-12->LUMO (3%), H-5->L+2 (9%), H-3->L+4 (7%)
185.1	0.0001	H-8->LUMO (98%)	
184.8	0.1211	H-26->LUMO (11%), H-14->L+1 (19%), H-5->L+1 (25%)	H-38->LUMO (2%), H-13->L+2 (4%), H-5->LUMO (3%), H-4->L+6 (9%), H-3->L+1 (4%)
184.6	0.3456	H-3->L+9 (30%), H-2->L+5 (20%), H-2->L+7 (36%)	H-2->L+4 (4%), H-2->L+12 (3%)
184.3	0.7287	H-13->L+4 (17%), H-12->L+2 (22%), H-12->L+4 (12%), H-4->L+6 (11%)	H-13->L+2 (9%), H-13->L+3 (2%), H-12->L+3 (3%), H-5->L+1 (3%), H-4->L+2 (4%)
183.9	0.031	H-14->L+2 (14%), H-13->L+1 (33%), H-4->L+1 (13%)	H-14->L+3 (3%), H-14->L+4 (2%), H-13->LUMO (8%), H-12->LUMO (3%), H-12->L+1 (4%), H-5->L+2 (3%), H-3->L+2 (3%)
183.2	0.295	H-16->L+3 (40%)	H-21->L+3 (4%), H-19->L+3 (2%), H-16->L+2 (7%), H-9->L+3 (6%), H-7->L+8 (8%), H-7->L+10 (3%), H-1->L+8 (5%), H-1->L+11 (7%), HOMO->L+8 (3%), HOMO->L+11 (3%)
183.1	0.0002	H-2->L+2 (14%), H-2->L+3 (85%)	
181.6	0.4987	H-5->L+6 (45%), H-4->L+5 (30%), H-3->L+6 (11%)	H-5->L+2 (2%), H-4->L+1 (2%), H-3->L+4 (3%)
181.2	0.3193	H-4->L+6 (18%), H-3->L+4 (33%), H-3->L+7 (12%)	H-13->L+4 (3%), H-12->L+2 (3%), H-12->L+4 (3%), H-5->L+5 (2%), H-3->L+2 (4%), H-3->L+5 (6%), H-2->L+9 (4%)
180.6	0.2925	H-4->L+6 (20%), H-3->L+4 (44%)	H-13->L+4 (2%), H-12->L+2 (3%), H-12->L+4 (3%), H-5->L+5 (7%), H-3->L+2 (5%), H-3->L+7 (3%)
179.1	0.0731	H-5->L+5 (13%), H-3->L+5 (36%), H-3->L+7 (23%)	H-5->L+1 (3%), H-4->L+6 (6%), H-3->L+1 (3%), H-3->L+4 (4%), H-2->L+9 (2%)
178.3	0.0179	H-46->LUMO (17%), H-14->L+4 (10%), H-9->LUMO (22%)	H-51->LUMO (7%), H-49->LUMO (2%), H-39->LUMO (2%), H-38->LUMO (5%), H-12->L+1 (6%)

WILEY-VCH

177.6	0.0054	H-9->LUMO (69%)	H-46->LUMO (5%), H-14->L+4 (6%), H-12->L+1 (4%)
177.0	0.0001	H-3->L+2 (14%), H-3->L+3 (85%)	
176.3	0.0016	HOMO->L+5 (98%)	
175.8	0.0063	H-2->L+6 (92%)	
175.8	0.0394	H-25->LUMO (30%)	H-49->LUMO (2%), H-46->LUMO (2%), H-38->LUMO (7%), H-25->L+1 (4%), H-14->L+2 (5%), H-14->L+4 (8%), H-13->L+1 (4%), H-12->L+1 (8%), H-9->LUMO (3%), H-2->L+6 (5%)
175.5	0.0721	HOMO->L+8 (34%), HOMO->L+10 (42%)	H-1->L+8 (6%), H-1->L+10 (7%), H-1->L+11 (2%), HOMO->L+5 (2%)
175.5	0.0029	H-10->LUMO (97%)	
174.1	0.187	H-14->L+2 (24%), H-14->L+4 (14%), H-13->L+1 (20%)	H-46->LUMO (4%), H-25->LUMO (2%), H-14->L+3 (5%), H-12->L+1 (9%)
173.9	0.2434	H-25->LUMO (24%), H-14->L+2 (16%), H-14->L+4 (10%), H-13->L+1 (11%)	H-36->LUMO (3%), H-25->L+1 (4%), H-14->L+3 (2%), H-12->L+1 (6%), H-5->L+4 (6%)
172.8	0.003	H-8->L+3 (20%), H-6->L+3 (45%)	H-15->L+3 (3%), H-10->L+3 (5%), H-8->L+2 (4%), H-6->L+2 (8%), H-1->L+10 (3%)
172.7	0.0476	H-1->L+8 (31%), H-1->L+10 (40%)	H-6->L+3 (3%), H-1->L+5 (3%), HOMO->L+8 (7%), HOMO->L+10 (6%), HOMO->L+11 (3%)
172.0	0.0013	H-1->L+5 (97%)	
171.7	0.001	H-4->L+2 (15%), H-4->L+4 (80%)	
171.4	0.0745	H-14->L+1 (21%), H-13->L+2 (18%), H-12->L+4 (19%)	H-46->LUMO (3%), H-31->LUMO (3%), H-26->LUMO (5%), H-16->LUMO (4%), H-13->L+3 (3%), H-7->L+1 (4%)
171.3	0.0005	H-11->LUMO (98%)	
170.4	0.012	H-2->L+12 (72%)	H-3->L+6 (6%), H-2->L+7 (3%), H-2->L+14 (7%), H-2->L+23 (3%)
170.1	0.0024	H-49->LUMO (22%), H-46->LUMO (11%)	H-56->L+1 (4%), H-54->LUMO (3%), H-52->LUMO (4%), H-48->LUMO (9%), H-47->LUMO (7%), H-45->LUMO (4%), H-26->LUMO (8%), H-25->LUMO (4%), H-16->LUMO (3%), H-14->L+1 (4%)
169.8	0.007	H-5->L+4 (22%), H-3->L+6 (47%)	H-5->L+2 (7%), H-5->L+6 (7%), H-2->L+12 (5%)
169.6	0.0014	H-8->L+3 (37%), H-6->L+3 (15%)	H-41->L+3 (3%), H-36->L+3 (5%), H-8->L+2 (7%), H-6->L+2 (3%), H-6->L+8 (3%), H-6->L+10 (5%)
169.5	0.0192	H-29->LUMO (12%), H-5->L+4 (25%), H-3->L+6 (19%)	H-29->L+1 (2%), H-29->L+5 (5%), H-27->LUMO (6%), H-27->L+5 (2%), H-16->LUMO (2%), H-5->L+2 (3%), H-5->L+6 (3%)
169.4	0.0269	H-29->LUMO (23%), H-29->L+5 (10%), H-27->LUMO (12%), H-5->L+4 (18%)	H-29->L+1 (5%), H-27->L+1 (2%), H-27->L+5 (5%), H-5->L+2 (3%), H-5->L+6 (2%), H-3->L+6 (8%)
168.9	0.0477	H-33->LUMO (15%), H-16->LUMO (26%)	H-56->LUMO (3%), H-29->LUMO (2%), H-26->LUMO (5%), H-26->L+1 (8%), H-13->L+2 (2%), H-5->L+4 (3%)
167.4	0.0015	H-6->L+8 (31%), H-6->L+10 (36%)	H-10->L+8 (2%), H-10->L+10 (3%), H-8->L+3 (2%), H-6->L+3 (7%), H-6->L+11 (2%)
166.9	0.1084	H-26->L+1 (10%), H-16->LUMO (43%)	H-56->LUMO (9%), H-33->LUMO (9%), H-15->LUMO (3%), H-7->L+1 (2%)
166.2	0.0014	H-15->LUMO (95%)	
165.3	0.0104	H-3->L+12 (80%)	H-3->L+14 (7%)
165.2	0.0107	H-31->LUMO (44%)	H-65->LUMO (3%), H-56->LUMO (7%), H-53->LUMO (4%), H-51->LUMO (3%), H-31->L+1 (7%), H-30->LUMO (3%), H-7->L+1 (2%)
164.9	0.0049	H-13->L+2 (13%), H-12->L+2 (48%), H-12->L+3 (10%), H-12->L+4 (19%)	H-13->L+3 (3%), H-13->L+4 (5%)
164.2	0.0011	HOMO->L+17 (13%), HOMO->L+18 (46%)	H-8->L+8 (2%), H-8->L+10 (3%), H-1->L+18 (2%), HOMO->L+13 (2%), HOMO->L+15 (9%), HOMO->L+16 (3%)
163.8	0.0134	H-56->LUMO (26%), H-31->LUMO (12%)	H-61->LUMO (3%), H-57->LUMO (5%), H-49->L+1 (4%), H-33->LUMO (9%), H-18->LUMO (5%), H-16->LUMO (2%), H-7->L+1 (2%)
163.7	0.0111	H-8->L+8 (28%), H-8->L+10 (33%)	H-15->L+8 (3%), H-15->L+10 (4%), H-6->L+8 (2%), H-6->L+11 (7%), HOMO->L+18 (4%)
163.4	0	HOMO->L+6 (100%)	
163.4	0.0003	H-17->LUMO (72%), H-17->L+7 (20%)	H-17->L+5 (5%)
163.3	0.0094	H-13->L+2 (12%), H-13->L+4 (62%), H-12->L+4 (12%)	H-12->L+2 (2%)

163.2	0.2295	H-9->L+3 (29%), H-7->L+8 (28%)	H-33->LUMO (3%), H-13->L+4 (3%), H-12->L+4 (2%), H-11->L+3 (3%), H-9->L+2 (5%), H-7->L+10 (9%)
162.4	0	H-17->LUMO (27%), H-17->L+5 (12%), H-17->L+7 (55%)	
162.2	0.0048	H-6->L+11 (73%)	H-10->L+11 (5%), H-8->L+8 (2%), H-8->L+11 (4%), H-6->L+10 (4%)
161.6	0.0107	H-18->LUMO (29%), H-7->L+1 (22%)	H-29->L+5 (4%), H-18->L+7 (3%), H-14->L+1 (3%), H-9->L+3 (7%)
161.3	0	H-4->L+2 (15%), H-4->L+3 (85%)	
161.2	0.0037	H-29->LUMO (17%), H-29->L+5 (36%), H-27->L+5 (19%)	H-27->LUMO (9%), H-18->LUMO (3%), H-7->L+1 (3%)
160.7	0.1521	H-18->LUMO (20%), H-9->L+3 (18%), H-7->L+8 (18%)	H-33->LUMO (2%), H-18->L+7 (3%), H-16->L+3 (6%), H-11->L+3 (4%), H-9->L+2 (3%), H-7->L+10 (5%)
160.1	0.0835	H-18->LUMO (22%), H-7->L+1 (32%), H-7->L+8 (10%)	H-33->LUMO (3%), H-33->L+5 (3%), H-26->L+5 (3%), H-14->L+5 (2%), H-9->L+3 (3%), H-7->L+10 (3%), H-5->L+3 (2%)
160.1	0.0014	H-5->L+2 (15%), H-5->L+3 (82%)	
160.0	0.0038	H-18->L+5 (16%), H-18->L+7 (55%)	H-18->LUMO (9%), H-7->L+1 (3%), H-5->L+7 (4%)
159.7	0	H-1->L+6 (100%)	
159.7	0.0095	H-7->L+2 (22%), H-7->L+4 (57%)	H-14->L+4 (5%), H-7->L+3 (3%)

Table S2.a: TD-DFT[C-PCM(CH_2Cl_2)/B3LYP(D3)/6-311G**] singlet electronic excitations for the investigated Am-Bpy^{2+}-Trz^{+} molecular thread. The derived UV/Vis absorption spectrum is shown in the associated manuscript in Fig 1 (middle upper panel).

Wavelength (nm)	Osc, Strength	Major contribs	Minor contribs
628.5	0.000	HOMO->LUMO (99%)	
568.0	0.000	H-1->LUMO (100%)	
540.3	0.000	H-2->LUMO (98%)	
469.3	0.000	H-3->LUMO (100%)	
438.2	0.009	H-5->LUMO (98%)	
435.7	0.000	H-4->LUMO (100%)	
414.1	0.000	H-6->LUMO (99%)	
367.1	0.000	HOMO->L+1 (69%), HOMO->L+2 (30%)	
351.0	0.000	HOMO->L+1 (30%), HOMO->L+2 (69%)	
347.9	0.001	HOMO->L+3 (16%), HOMO->L+4 (82%)	
345.6	0.000	HOMO->L+3 (83%), HOMO->L+4 (16%)	
345.5	0.000	H-1->L+1 (74%), H-1->L+2 (26%)	
341.4	0.006	H-3->L+1 (42%), H-3->L+2 (57%)	
335.5	0.000	H-1->L+1 (26%), H-1->L+2 (74%)	
335.0	0.000	H-2->L+1 (70%), H-2->L+2 (28%)	
328.6	0.000	H-1->L+3 (97%)	H-1->L+4 (3%)
326.1	0.000	H-1->L+4 (97%)	H-1->L+3 (3%)
323.9	0.035	H-4->L+1 (42%), H-4->L+2 (57%)	
323.6	0.000	H-2->L+1 (29%), H-2->L+2 (71%)	
318.5	0.002	H-2->L+3 (63%), H-2->L+4 (35%)	
317.4	0.001	H-2->L+3 (36%), H-2->L+4 (61%)	
305.5	0.000	H-3->L+1 (58%), H-3->L+2 (42%)	
296.1	0.000	H-3->L+3 (100%)	
292.9	0.001	H-5->L+1 (63%), H-5->L+2 (33%)	H-5->L+4 (2%)

290.9	0.000	H-4->L+1 (58%), H-4->L+2 (42%)	
289.8	0.071	H-8->LUMO (86%)	H-9->LUMO (5%), H-7->LUMO (8%)
287.1	0.000	H-3->L+4 (100%)	
284.9	0.010	H-5->L+4 (93%)	
282.6	0.000	H-7->LUMO (92%)	H-8->LUMO (8%)
282.3	0.000	H-4->L+3 (100%)	
281.9	0.000	H-6->L+1 (63%), H-6->L+2 (33%)	H-6->L+4 (3%)
281.4	0.012	HOMO->L+5 (89%)	H-2->L+5 (9%)
277.9	0.001	H-9->LUMO (94%)	H-8->LUMO (5%)
274.1	0.000	H-4->L+4 (100%)	
273.9	0.007	H-6->L+4 (93%)	H-6->L+1 (2%)
273.2	0.003	H-5->L+3 (91%)	H-5->L+1 (2%), H-5->L+2 (4%)
272.1	0.000	H-5->L+1 (32%), H-5->L+2 (62%)	H-5->L+3 (6%)
268.5	0.073	H-16->LUMO (89%)	H-17->L+4 (3%), H-10->LUMO (3%)
267.4	0.010	H-10->LUMO (96%)	H-16->LUMO (2%)
264.2	0.001	H-6->L+3 (92%)	H-6->L+1 (2%), H-6->L+2 (3%)
263.1	0.002	H-6->L+1 (31%), H-6->L+2 (60%)	H-19->LUMO (4%), H-6->L+3 (4%)
263.0	0.049	H-19->LUMO (86%)	H-17->L+3 (4%), H-6->L+2 (2%)
261.8	0.001	H-2->L+8 (10%), HOMO->L+8 (87%)	
261.1	0.732	H-17->LUMO (69%), H-13->LUMO (19%)	H-18->LUMO (5%)
260.1	0.017	H-13->LUMO (27%), H-11->LUMO (71%)	
260.0	0.155	H-17->LUMO (16%), H-13->LUMO (54%), H-11->LUMO (28%)	
258.7	0.000	H-12->LUMO (100%)	
255.0	0.001	H-1->L+5 (97%)	
254.8	0.001	H-14->LUMO (99%)	
250.6	0.033	H-2->L+5 (89%)	HOMO->L+5 (9%)
250.2	0.000	H-15->LUMO (100%)	
244.0	0.001	H-18->LUMO (90%)	H-17->LUMO (7%)
241.6	0.015	H-4->L+7 (17%), H-4->L+9 (18%), H-3->L+7 (45%), H-3->L+9 (14%)	H-4->L+6 (5%)
241.0	0.003	H-2->L+11 (11%), H-1->L+10 (25%), H-1->L+11 (16%), HOMO->L+10 (20%), HOMO->L+11 (13%)	H-2->L+10 (6%), H-1->L+5 (2%), H-1->L+8 (7%)
238.9	0.002	H-6->L+5 (36%), H-5->L+8 (24%), H-2->L+8 (31%)	HOMO->L+8 (7%)
237.6	0.001	H-1->L+8 (91%)	
234.5	0.000	HOMO->L+6 (97%)	
233.8	0.004	H-22->LUMO (22%), H-21->LUMO (74%)	
232.8	0.000	HOMO->L+7 (98%)	HOMO->L+6 (2%)
231.9	0.006	H-6->L+5 (19%), H-5->L+8 (18%), H-2->L+8 (55%)	HOMO->L+8 (4%)
230.5	0.005	H-22->LUMO (72%), H-21->LUMO (16%)	H-24->LUMO (4%), H-20->LUMO (6%)
230.4	0.007	H-2->L+10 (13%), HOMO->L+10 (62%), HOMO->L+11 (18%)	H-1->L+10 (5%)
227.9	0.001	H-20->LUMO (89%)	H-22->LUMO (3%), H-21->LUMO (5%)
227.4	0.000	H-1->L+6 (97%)	
227.3	0.005	H-3->L+6 (95%)	
226.5	0.012	H-7->L+1 (41%), H-7->L+2 (54%)	H-8->L+2 (2%)
226.0	0.000	H-1->L+7 (98%)	

223.4	0.010	H-2->L+11 (22%), HOMO->L+11 (57%)	H-2->L+10 (9%), H-1->L+10 (3%), H-1->L+11 (2%), HOMO->L+10 (6%)
223.4	0.000	H-3->L+5 (100%)	
222.8	0.046	H-9->L+1 (32%), H-9->L+2 (42%)	H-8->L+1 (7%), H-8->L+2 (9%), H-4->L+7 (3%)
221.9	0.000	H-2->L+6 (97%)	
220.7	0.044	H-8->L+1 (11%), H-4->L+6 (44%), H-4->L+7 (14%)	H-9->L+1 (7%), H-9->L+2 (7%), H-8->L+2 (8%), H-3->L+7 (2%), H-3->L+9 (4%)
220.5	0.000	H-2->L+7 (98%)	H-2->L+6 (2%)
218.8	0.026	H-23->LUMO (40%), H-4->L+6 (15%), H-3->L+7 (10%)	H-31->LUMO (6%), H-24->LUMO (2%), H-8->L+1 (4%), H-4->L+7 (5%), H-4->L+9 (2%), H-3->L+9 (5%)
218.8	0.002	H-23->LUMO (31%), H-4->L+6 (21%), H-3->L+7 (14%)	H-31->LUMO (4%), H-4->L+7 (8%), H-4->L+9 (3%), H-3->L+9 (8%)
218.2	0.025	H-27->LUMO (44%), H-24->LUMO (21%), H-23->LUMO (10%)	H-31->LUMO (3%), H-8->L+1 (5%), H-5->L+5 (6%)
218.1	0.040	H-27->LUMO (15%), H-8->L+1 (43%)	H-28->LUMO (2%), H-23->LUMO (4%), H-9->L+1 (3%), H-8->L+2 (6%), H-7->L+1 (3%), H-5->L+5 (5%), H-4->L+7 (3%)
217.9	0.000	HOMO->L+9 (100%)	
217.8	0.183	H-27->LUMO (17%), H-6->L+8 (10%), H-5->L+5 (59%)	H-28->LUMO (2%), H-23->LUMO (4%)
217.4	0.082	H-29->LUMO (19%), H-28->LUMO (26%), H-8->L+1 (12%)	H-24->LUMO (3%), H-8->L+2 (8%), H-5->L+5 (7%), H-4->L+6 (4%), H-4->L+7 (5%)
217.0	0.050	H-29->LUMO (17%), H-28->LUMO (21%), H-8->L+2 (25%)	H-31->LUMO (5%), H-27->LUMO (2%), H-24->LUMO (4%), H-23->LUMO (4%), H-4->L+6 (3%), H-4->L+7 (4%)
216.3	0.013	H-27->LUMO (15%), H-24->LUMO (41%), H-8->L+2 (24%)	H-31->LUMO (2%)
215.4	0.000	H-4->L+5 (100%)	
214.5	0.005	H-31->LUMO (43%), H-24->LUMO (19%)	H-27->LUMO (2%), H-23->LUMO (3%), H-17->L+1 (8%), H-17->L+2 (4%), H-8->L+2 (6%)
214.3	0.001	H-8->L+3 (75%)	H-19->L+1 (5%), H-19->L+2 (3%), H-17->L+3 (2%), H-9->L+3 (6%), H-7->L+3 (6%)
214.1	0.006	H-2->L+10 (30%), H-2->L+11 (15%), H-1->L+10 (24%), H-1->L+11 (27%)	
213.7	0.000	H-25->LUMO (21%), H-7->L+1 (41%), H-7->L+2 (31%)	H-8->L+1 (4%), H-8->L+2 (3%)
213.6	0.000	H-25->LUMO (79%), H-7->L+1 (10%)	H-7->L+2 (8%)
212.3	0.012	H-17->L+4 (17%), H-16->L+1 (40%), H-16->L+2 (23%)	H-26->LUMO (9%), H-8->L+4 (3%)
212.3	0.001	H-26->LUMO (89%)	H-16->L+1 (4%), H-16->L+2 (2%)
212.0	0.000	H-1->L+9 (100%)	
210.9	0.002	H-11->L+2 (11%), H-9->L+1 (46%), H-9->L+2 (30%)	H-11->L+1 (8%), H-8->L+1 (2%)
210.8	0.006	H-11->L+1 (32%), H-11->L+2 (41%), H-9->L+2 (11%)	H-9->L+1 (8%)
209.9	0.000	H-3->L+8 (99%)	
209.7	0.001	H-12->L+1 (41%), H-12->L+2 (53%)	
208.9	0.000	H-7->L+3 (91%)	H-19->L+1 (2%), H-8->L+3 (3%)
208.5	0.004	H-19->L+1 (35%), H-19->L+2 (20%), H-17->L+3 (22%), H-8->L+3 (15%)	H-7->L+3 (2%)
207.1	0.000	H-2->L+9 (100%)	
206.4	0.003	H-8->L+4 (81%), H-7->L+4 (10%)	H-16->L+1 (2%), H-9->L+4 (3%)
206.3	0.000	H-9->L+3 (93%)	H-8->L+3 (5%)

Table S2.b: TD-DFT[C-PCM(CH_2Cl_2)/CAM-B3LYP(D3)/6-311G**] singlet electronic excitations for the investigated Am-Bpy^{2+}-Trz^{+} molecular thread. The derived UV/Vis absorption spectrum is shown in the associated manuscript in Fig 1 (middle lower panel).

Wavelength (nm)	Osc, Strength	Major contribs	Minor contribs
309.1	0.0044	H-5->LUMO (22%), H-2->LUMO (39%), HOMO->LUMO (33%)	
297.4	0.008	H-5->LUMO (30%), HOMO->LUMO (59%)	H-2->LUMO (6%)
290.9	0	H-1->LUMO (97%)	HOMO->LUMO (2%)
277.4	0.008	H-6->LUMO (58%), H-5->LUMO (15%), H-2->LUMO (18%)	H-6->L+1 (2%), H-6->L+2 (2%), HOMO->LUMO (2%)
273.7	0.0027	H-6->LUMO (34%), H-5->LUMO (22%), H-2->LUMO (36%)	HOMO->LUMO (3%)
260.2	0.0002	H-3->LUMO (98%)	
255.3	0.0219	H-3->L+1 (40%), H-3->L+2 (40%)	H-4->L+1 (5%), H-4->L+2 (4%), H-3->L+6 (3%)
248.9	0.0075	H-4->LUMO (97%)	
248.1	0.143	H-14->LUMO (78%)	H-15->L+4 (6%), H-14->L+1 (4%), H-14->L+2 (3%)
246.5	0.0593	H-16->LUMO (80%)	H-16->L+1 (3%), H-16->L+2 (3%), H-15->L+3 (6%)
245.2	0.0868	H-4->L+1 (40%), H-4->L+2 (40%)	H-4->LUMO (3%), H-4->L+6 (3%), H-3->L+1 (3%), H-3->L+2 (3%)
242.1	1.0169	H-15->LUMO (85%)	H-7->LUMO (4%)
235.1	0.0005	H-6->L+5 (39%), H-5->L+4 (11%), H-5->L+7 (30%), H-2->L+7 (10%)	H-2->L+4 (5%)
234.6	0.0024	H-1->L+10 (21%), H-1->L+11 (27%), HOMO->L+10 (27%), HOMO->L+11 (11%)	H-2->L+10 (5%), H-2->L+11 (6%)
231.4	0.0061	H-4->L+6 (17%), H-4->L+9 (16%), H-3->L+6 (36%), H-3->L+9 (12%)	H-4->L+8 (3%), H-3->L+1 (6%), H-3->L+2 (6%)
225.8	0.0559	H-2->L+4 (11%), H-2->L+5 (39%), HOMO->L+5 (23%)	H-5->L+4 (6%), HOMO->L+4 (6%)
221.8	0.0068	H-5->L+4 (24%), H-2->L+4 (17%)	H-6->L+5 (7%), H-5->L+7 (4%), H-2->L+1 (4%), H-2->L+2 (4%), H-2->L+5 (9%), HOMO->L+1 (5%), HOMO->L+2 (4%), HOMO->L+4 (4%), HOMO->L+5 (8%)
218.9	0.002	HOMO->L+1 (32%), HOMO->L+2 (27%)	H-6->L+5 (2%), H-5->L+4 (5%), H-2->L+1 (9%), H-2->L+2 (9%), H-2->L+4 (4%), H-2->L+5 (3%), HOMO->L+5 (2%)
215.6	0.0411	H-5->L+4 (21%), HOMO->L+4 (52%)	H-5->L+5 (3%), H-2->L+5 (3%), H-2->L+7 (5%), HOMO->L+7 (6%)
212.8	0.0634	H-7->L+1 (28%), H-7->L+2 (25%), H-4->L+6 (14%), H-3->L+6 (14%)	H-3->L+9 (8%)
212.7	0.0453	H-5->L+5 (18%)	H-6->L+4 (9%), H-6->L+7 (5%), H-5->LUMO (2%), H-5->L+1 (7%), H-5->L+2 (7%), H-2->L+1 (9%), H-2->L+2 (8%), H-1->L+10 (3%), HOMO->L+1 (6%), HOMO->L+2 (5%), HOMO->L+5 (4%), HOMO->L+10 (3%), HOMO->L+11 (3%)
212.2	0.0004	H-1->L+1 (52%), H-1->L+2 (43%)	
211.8	0.0147	H-6->L+4 (11%), H-5->L+5 (13%), H-2->L+1 (10%)	H-6->L+7 (4%), H-5->L+1 (6%), H-5->L+2 (6%), H-5->L+4 (5%), H-2->L+2 (9%), H-1->L+10 (4%), HOMO->L+1 (6%), HOMO->L+2 (5%), HOMO->L+5 (3%), HOMO->L+10 (3%), HOMO->L+11 (3%)
210.8	0.1238	H-7->L+1 (19%), H-7->L+2 (17%), H-4->L+6 (21%), H-3->L+6 (18%), H-3->L+9 (12%)	H-4->L+9 (4%), H-3->L+8 (2%)
210.2	0.0068	H-6->L+4 (15%), H-1->L+10 (25%), H-1->L+11 (12%), HOMO->L+10 (12%), HOMO->L+11 (16%)	H-6->L+7 (2%), H-5->L+5 (6%), H-2->L+10 (3%), H-2->L+11 (2%)
208.0	0.0006	HOMO->L+3 (87%)	H-2->L+3 (8%)
207.6	0.0004	H-1->L+4 (88%)	H-1->L+3 (3%), HOMO->L+4 (6%)
206.6	0.2246	H-6->L+4 (47%), H-5->L+5 (25%)	H-6->L+7 (3%), H-2->L+4 (3%), HOMO->L+4 (5%)
205.9	0.0117	H-2->L+7 (31%), HOMO->L+4 (17%), HOMO->L+7 (26%)	H-6->L+4 (4%), H-6->L+5 (4%), H-5->L+7 (3%), H-2->L+4 (2%), H-1->L+4 (4%)
203.9	0	H-1->L+3 (95%)	H-1->L+4 (3%)
203.3	0	HOMO->L+1 (46%), HOMO->L+2 (52%)	

202.3	0.0041	H-5->L+1 (24%), H-5->L+2 (22%), H-2->L+1 (16%), H-2->L+2 (13%)	H-5->LUMO (3%), H-2->L+4 (5%), HOMO->L+2 (2%)
201.3	0	H-1->L+1 (44%), H-1->L+2 (54%)	
201.1	0.0072	H-31->LUMO (10%), H-7->LUMO (64%)	H-15->LUMO (2%), H-14->L+4 (3%), H-7->L+2 (2%)
200.1	0.0114	H-5->L+4 (15%), H-2->L+3 (29%), H-2->L+4 (27%)	H-6->L+1 (5%), H-6->L+2 (4%), H-2->L+7 (3%), HOMO->L+3 (3%), HOMO->L+4 (2%), HOMO->L+7 (5%)
199.8	0.0057	H-2->L+3 (54%), H-2->L+4 (13%)	H-6->L+1 (7%), H-6->L+2 (7%), H-5->L+3 (4%), H-5->L+4 (3%), HOMO->L+3 (5%)
198.9	0.0481	H-6->L+1 (31%), H-6->L+2 (29%)	H-6->LUMO (4%), H-6->L+4 (5%), H-5->L+1 (3%), H-5->L+2 (2%), H-5->L+4 (4%), H-2->L+1 (3%), H-2->L+4 (9%)
195.5	0.0028	H-31->LUMO (17%), H-16->L+3 (14%), H-14->L+4 (12%), H-7->LUMO (22%)	H-43->LUMO (3%), H-28->LUMO (2%), H-15->L+1 (6%), H-15->L+2 (6%)
194.5	0.001	H-3->L+1 (49%), H-3->L+2 (50%)	
194.4	0.0001	H-2->L+1 (45%), H-2->L+2 (53%)	
193.6	0.1445	H-2->L+10 (20%), H-1->L+11 (19%), HOMO->L+10 (34%)	H-2->L+11 (7%), HOMO->L+11 (6%)
192.7	0.0002	H-3->L+3 (99%)	
192.0	0.8648	H-4->L+6 (28%), H-3->L+9 (37%)	H-20->L+1 (5%), H-20->L+2 (5%), H-19->L+1 (4%), H-19->L+2 (4%), H-9->L+1 (2%), H-9->L+2 (2%), H-3->L+8 (7%)
190.3	0.9283	H-1->L+5 (12%), H-1->L+10 (20%), HOMO->L+11 (45%)	H-6->L+7 (3%), H-1->L+11 (8%), HOMO->L+10 (7%)
188.8	0.4476	H-6->L+7 (13%), H-2->L+5 (10%), H-2->L+10 (19%), H-1->L+11 (10%), HOMO->L+5 (16%)	H-14->L+4 (3%), H-2->L+11 (7%), H-1->L+10 (9%), HOMO->L+11 (3%)
188.4	0.0076	H-5->L+3 (81%)	H-15->L+3 (5%), H-2->L+3 (5%)
188.2	0.0404	H-4->L+1 (48%), H-4->L+2 (46%)	
187.6	0.899	H-4->L+9 (45%), H-3->L+6 (19%)	H-20->L+1 (2%), H-20->L+2 (2%), H-4->L+2 (3%), H-4->L+8 (9%), H-3->L+9 (3%)
186.7	0.048	H-15->L+4 (20%), H-14->LUMO (12%), H-14->L+1 (20%), H-14->L+2 (20%)	H-31->L+4 (2%), H-15->L+3 (4%), H-14->L+4 (2%), H-5->L+3 (2%)
186.5	0.0004	H-4->L+3 (98%)	
185.9	0.2854	H-31->LUMO (12%), H-15->L+1 (12%), H-15->L+2 (12%), H-14->L+4 (21%)	H-52->LUMO (3%), H-49->LUMO (3%), H-43->LUMO (2%), H-6->L+7 (3%)
185.8	0	H-3->L+4 (99%)	
185.1	0.2168	H-6->L+3 (15%), H-6->L+5 (22%), H-5->L+7 (27%), H-1->L+5 (14%)	H-6->L+7 (4%), H-2->L+7 (3%), HOMO->L+7 (4%)
184.9	0.8146	H-16->L+3 (20%), H-6->L+7 (15%)	H-31->LUMO (4%), H-15->L+1 (6%), H-15->L+2 (6%), H-15->L+4 (3%), H-14->L+4 (8%), H-5->L+5 (3%), H-1->L+11 (3%)
184.8	0.0768	H-1->L+5 (61%)	H-6->L+3 (5%), H-6->L+5 (3%), H-5->L+7 (4%), H-1->L+10 (6%), HOMO->L+5 (4%), HOMO->L+11 (6%)
184.6	0.1065	H-4->L+6 (10%), H-4->L+9 (10%)	H-20->L+1 (7%), H-20->L+2 (6%), H-19->L+1 (6%), H-19->L+2 (6%), H-9->L+1 (5%), H-9->L+2 (5%), H-8->LUMO (7%), H-7->L+8 (4%), H-6->L+7 (2%), H-4->L+8 (2%), H-3->L+6 (4%), H-3->L+8 (4%), H-3->L+9 (4%)
184.2	0.0105	H-8->LUMO (91%)	
183.9	0.1378	H-15->L+3 (14%), H-6->L+3 (42%)	H-16->LUMO (5%), H-16->L+1 (5%), H-16->L+2 (5%), H-6->L+5 (7%), H-5->L+3 (4%), H-5->L+7 (6%)
183.5	0.0367	H-15->L+3 (21%), H-6->L+3 (32%)	H-16->LUMO (8%), H-16->L+1 (9%), H-16->L+2 (9%), H-6->L+5 (3%), H-5->L+3 (2%)
182.7	0.2372	H-2->L+5 (24%), H-2->L+10 (11%), HOMO->L+5 (24%)	H-16->L+3 (3%), H-14->L+4 (2%), H-6->L+7 (2%), H-2->L+11 (4%), H-1->L+5 (5%), H-1->L+11 (7%), HOMO->L+10 (6%)
182.4	0.0004	H-9->LUMO (97%)	
182.3	0.01	H-22->LUMO (11%), H-10->LUMO (67%)	
181.3	0.0296	H-45->L+1 (11%), H-45->L+2 (11%), H-39->L+1 (21%), H-39->L+2 (20%)	H-33->L+1 (2%), H-33->L+2 (2%), H-32->L+1 (5%), H-32->L+2 (5%), H-27->L+1 (3%), H-27->L+2 (3%)
181.0	0.4525	H-16->L+3 (16%), H-6->L+7 (17%), H-2->L+11 (19%)	H-14->L+4 (8%), H-5->L+5 (8%), H-2->L+10 (9%), H-1->L+5 (2%), HOMO->L+5 (3%)
180.8	0.2573	H-6->L+7 (13%), H-2->L+10 (13%), H-2->L+11 (37%)	H-16->L+3 (9%), H-14->L+4 (4%), H-5->L+5 (6%), H-1->L+10 (3%), H-1->L+11 (3%)

WILEY-VCH

179.9	0.0001	H-4->L+4 (99%)	
179.2	0.0034	H-51->LUMO (13%), H-49->LUMO (10%), H-22->LUMO (12%), H-21->LUMO (26%)	H-48->LUMO (3%), H-45->LUMO (2%), H-13->LUMO (3%), H-10->LUMO (6%)
178.0	0	H-5->L+1 (48%), H-5->L+2 (51%)	
177.4	0.0085	H-3->L+8 (73%), H-3->L+9 (15%)	H-4->L+8 (5%)
177.0	0.006	H-51->LUMO (15%), H-21->LUMO (23%), H-13->LUMO (15%)	H-52->LUMO (2%), H-43->LUMO (3%), H-22->LUMO (4%), H-16->L+1 (3%), H-16->L+2 (3%), H-15->L+3 (2%), H-15->L+4 (5%), H-14->L+1 (2%), H-14->L+2 (2%), H-10->LUMO (2%)
176.2	0.0032	H-1->L+7 (80%), HOMO->L+7 (14%)	H-2->L+7 (3%)
175.2	0.0132	H-27->LUMO (17%), H-13->LUMO (17%)	H-43->LUMO (3%), H-22->LUMO (3%), H-16->L+1 (8%), H-16->L+2 (8%), H-15->L+3 (6%), H-15->L+4 (4%), H-14->L+1 (3%), H-14->L+2 (3%)
175.1	0.0131		H-13->LUMO (5%), H-2->L+7 (2%), H-2->L+10 (2%), H-2->L+11 (4%), H-2->L+19 (6%), H-2->L+20 (3%), H-2->L+21 (6%), H-2->L+25 (5%), H-2->L+26 (2%), H-2->L+33 (6%), HOMO->L+19 (7%), HOMO->L+20 (4%), HOMO->L+21 (7%), HOMO->L+25 (5%), HOMO->L+33 (4%)
175.0	0.0015	H-13->LUMO (12%), H-8->L+1 (23%), H-8->L+2 (22%), H-8->L+6 (17%)	H-27->LUMO (3%)
175.0	0.0051	H-27->LUMO (14%), H-13->LUMO (37%)	H-52->LUMO (2%), H-51->LUMO (3%), H-43->LUMO (3%), H-21->LUMO (2%), H-8->L+1 (6%), H-8->L+2 (6%), H-8->L+6 (5%)
174.5	0.0039	H-2->L+7 (29%), H-1->L+7 (14%), HOMO->L+7 (32%)	H-5->L+7 (9%)
173.9	0.0014	H-6->L+1 (48%), H-6->L+2 (51%)	
173.8	0.0954	H-27->LUMO (14%), H-15->L+4 (20%)	H-51->LUMO (4%), H-45->LUMO (4%), H-39->LUMO (2%), H-21->LUMO (6%), H-14->L+1 (8%), H-14->L+2 (8%), H-13->LUMO (5%)
173.7	0.0205	H-4->L+8 (69%), H-4->L+9 (13%)	H-11->LUMO (4%), H-3->L+8 (5%)
173.5	0.0044	H-11->LUMO (90%)	H-12->LUMO (2%), H-4->L+8 (4%)
172.8	0.3151	H-16->L+1 (14%), H-16->L+2 (14%), H-15->L+3 (16%), H-15->L+4 (20%)	H-52->LUMO (3%), H-27->LUMO (6%), H-14->L+1 (4%), H-14->L+2 (3%)
172.7	0.001	H-12->LUMO (97%)	H-11->LUMO (2%)
172.2	0.0166	H-22->LUMO (12%), H-21->LUMO (13%), H-18->LUMO (17%), H-10->LUMO (12%)	H-52->LUMO (2%), H-51->LUMO (3%), H-28->LUMO (5%), H-24->LUMO (3%), H-15->L+1 (3%), H-15->L+2 (3%), H-14->L+4 (3%)
171.2	0.0211	H-52->LUMO (21%), H-49->LUMO (13%)	H-53->LUMO (2%), H-50->LUMO (3%), H-28->LUMO (8%), H-27->LUMO (8%), H-22->LUMO (9%), H-18->LUMO (6%)
170.8	0.1082	H-16->L+3 (10%), H-14->L+4 (10%)	H-31->LUMO (4%), H-21->LUMO (2%), H-20->LUMO (2%), H-20->L+1 (2%), H-19->LUMO (4%), H-15->L+1 (7%), H-15->L+2 (5%), H-9->L+1 (6%), H-9->L+2 (5%), H-9->L+6 (6%), H-7->L+2 (2%), H-7->L+8 (5%)
170.0	0.0008	H-9->L+1 (10%), H-9->L+2 (10%), H-9->L+6 (14%)	H-20->L+1 (4%), H-20->L+2 (4%), H-18->LUMO (8%), H-16->L+3 (6%), H-15->L+1 (2%), H-15->L+2 (4%), H-14->L+4 (5%), H-7->L+8 (5%)
169.8	0.0045	H-22->LUMO (15%), H-18->LUMO (59%)	H-40->LUMO (3%), H-19->LUMO (2%)
168.7	0.0002	H-17->LUMO (99%)	
167.9	0.0103	H-19->LUMO (74%)	H-28->LUMO (2%), H-20->LUMO (8%)
166.7	0.0088	H-8->L+6 (52%), H-8->L+9 (13%)	H-12->L+6 (3%), H-8->L+1 (8%), H-8->L+2 (8%), H-8->L+8 (2%)
166.1	0.0011	H-14->L+3 (89%)	H-16->L+3 (2%), H-15->L+3 (3%), H-13->L+3 (2%)
165.8	0.0154	H-62->LUMO (26%), H-28->LUMO (12%)	H-61->LUMO (4%), H-52->L+1 (2%), H-52->L+2 (2%), H-33->LUMO (4%), H-31->LUMO (9%), H-30->LUMO (2%), H-27->LUMO (3%)
164.4	0.0149	H-33->LUMO (41%), H-32->LUMO (11%)	H-62->LUMO (5%), H-49->LUMO (2%), H-39->LUMO (3%), H-33->L+1 (2%), H-33->L+2 (2%), H-22->LUMO (3%), H-9->L+6 (3%)
164.1	0.0574	H-9->L+6 (42%), H-7->L+8 (11%)	H-33->LUMO (2%), H-19->L+6 (3%), H-9->L+1 (5%), H-9->L+2 (4%), H-9->L+9 (7%)
163.4	0.0585	H-62->LUMO (11%), H-28->LUMO (26%)	H-39->LUMO (3%), H-28->L+1 (2%), H-28->L+2 (2%), H-22->LUMO (2%), H-20->LUMO (3%), H-19->LUMO (3%), H-14->L+4 (2%)
162.6	0.003	H-8->L+9 (54%)	H-12->L+9 (3%), H-8->L+1 (6%), H-8->L+2 (5%), H-8->L+6 (7%), H-8->L+8 (7%)

162.3	0.0104	H-1->L+19 (15%), H-1->L+20 (18%), H-1->L+21 (15%)	H-1->L+24 (8%), H-1->L+26 (6%), HOMO->L+19 (4%), HOMO->L+20 (5%), HOMO->L+24 (2%), HOMO->L+25 (3%)
162.2	0.0026	H-21->L+5 (70%)	H-24->L+5 (5%), H-16->L+4 (4%), H-10->L+5 (4%)
162.2	0.0003	H-16->L+4 (93%)	H-21->L+5 (3%)
162.0	0.1895	H-11->L+1 (17%), H-11->L+2 (17%), H-7->L+8 (21%)	H-12->L+1 (7%), H-12->L+2 (6%), H-7->L+9 (2%)

Table S3.a: TD-DFT[C-PCM(CH_2Cl_2)/B3LYP(D3)/6-311G**] singlet electronic excitations for the investigated Am-Bpy-Trz^+ molecular thread. The derived UV/Vis absorption spectrum is shown in the associated manuscript in Fig 1 (right upper panel).

Wavelength (nm)	Osc, Strength	Major contribs	Minor contribs
1773.2	0.010	HOMO->LUMO (100%)	
545.2	0.000	HOMO->L+4 (70%), HOMO->L+6 (18%)	HOMO->L+7 (7%), HOMO->L+8 (3%)
508.4	0.001	HOMO->L+1 (100%)	
498.8	0.032	HOMO->L+2 (99%)	
470.3	0.011	HOMO->L+5 (94%)	
461.6	0.003	HOMO->L+4 (25%), HOMO->L+6 (41%), HOMO->L+7 (12%), HOMO->L+8 (12%)	HOMO->L+9 (3%), HOMO->L+10 (7%)
443.2	0.003	HOMO->L+3 (99%)	
412.3	0.017	HOMO->L+10 (82%)	HOMO->L+4 (3%), HOMO->L+6 (9%), HOMO->L+8 (3%)
399.8	0.010	HOMO->L+7 (20%), HOMO->L+8 (69%)	HOMO->L+10 (7%)
386.8	0.013	HOMO->L+9 (91%)	HOMO->L+8 (5%)
374.9	1.386	HOMO->L+6 (30%), HOMO->L+7 (57%)	HOMO->L+8 (6%), HOMO->L+9 (2%), HOMO->L+10 (3%)
359.2	0.000	H-1->LUMO (100%)	
344.1	0.003	H-2->LUMO (92%)	H-4->LUMO (7%)
335.3	0.003	HOMO->L+11 (93%)	HOMO->L+12 (3%), HOMO->L+14 (2%)
331.7	0.006	H-6->LUMO (99%)	
331.1	0.000	H-3->LUMO (99%)	
327.4	0.000	H-4->LUMO (92%)	H-2->LUMO (7%)
315.4	0.031	H-8->LUMO (99%)	
307.6	0.000	H-5->LUMO (99%)	
305.3	0.004	HOMO->L+12 (25%), HOMO->L+14 (46%)	HOMO->L+11 (5%), HOMO->L+13 (7%), HOMO->L+18 (8%), HOMO->L+19 (2%)
299.2	0.002	HOMO->L+12 (67%), HOMO->L+14 (11%)	HOMO->L+13 (9%), HOMO->L+18 (7%)
292.4	0.001	HOMO->L+13 (51%), HOMO->L+15 (19%), HOMO->L+18 (17%)	HOMO->L+16 (5%), HOMO->L+21 (6%)
292.3	0.000	H-7->LUMO (100%)	
286.9	0.002	HOMO->L+14 (12%), HOMO->L+15 (19%), HOMO->L+16 (22%), HOMO->L+18 (32%)	HOMO->L+12 (4%), HOMO->L+21 (4%)
283.4	0.000	H-10->LUMO (11%), H-9->LUMO (89%)	
277.8	0.001	HOMO->L+13 (30%), HOMO->L+14 (15%), HOMO->L+15 (33%), HOMO->L+21 (14%)	
273.6	0.000	HOMO->L+16 (30%), HOMO->L+18 (13%), HOMO->L+19 (21%), HOMO->L+21 (14%)	HOMO->L+14 (3%), HOMO->L+15 (7%), HOMO->L+20 (5%), HOMO->L+26 (2%)
272.9	0.000	H-10->LUMO (89%), H-9->LUMO (11%)	
270.6	0.000	HOMO->L+16 (28%), HOMO->L+17 (10%), HOMO->L+21 (21%)	HOMO->L+15 (8%), HOMO->L+18 (8%), HOMO->L+19 (4%), HOMO->L+20 (5%), HOMO->L+22 (6%), HOMO->L+24 (3%)
268.2	0.002	HOMO->L+19 (35%), HOMO->L+23 (15%)	HOMO->L+14 (6%), HOMO->L+15 (7%), HOMO->L+16 (6%), HOMO->L+18 (4%), HOMO->L+20 (6%), HOMO->L+21 (8%), HOMO->L+24 (2%), HOMO->L+27 (2%)

265.2	0.000	HOMO->L+17 (82%)	HOMO->L+15 (3%), HOMO->L+16 (3%), HOMO->L+21 (4%), HOMO->L+24 (2%)
256.8	0.001	HOMO->L+20 (44%), HOMO->L+22 (12%), HOMO->L+23 (25%)	HOMO->L+18 (3%), HOMO->L+21 (5%), HOMO->L+26 (4%)
255.7	0.001	HOMO->L+19 (24%), HOMO->L+20 (30%), HOMO->L+22 (26%)	HOMO->L+23 (6%), HOMO->L+25 (4%), HOMO->L+26 (3%)
252.3	0.001	HOMO->L+24 (30%), HOMO->L+25 (30%)	HOMO->L+20 (3%), HOMO->L+21 (9%), HOMO->L+22 (2%), HOMO->L+26 (9%), HOMO->L+30 (3%), HOMO->L+32 (4%)
249.8	0.002	HOMO->L+22 (39%), HOMO->L+23 (36%)	HOMO->L+18 (3%), HOMO->L+19 (7%), HOMO->L+27 (5%), HOMO->L+28 (5%)
248.6	0.013	H-1->L+4 (58%), H-1->L+5 (25%)	H-1->L+6 (6%), H-1->L+7 (5%)
247.2	0.003	H-1->L+4 (16%), H-1->L+5 (61%)	H-7->L+4 (2%), H-7->L+5 (3%), H-4->L+5 (2%), H-2->L+4 (2%), H-1->L+6 (7%)
245.8	0.003	HOMO->L+26 (23%), HOMO->L+27 (15%), HOMO->L+28 (15%)	HOMO->L+20 (2%), HOMO->L+21 (6%), HOMO->L+24 (5%), HOMO->L+25 (6%), HOMO->L+30 (8%), HOMO->L+33 (8%), HOMO->L+36 (2%)
242.3	0.004	H-4->L+8 (10%), H-3->L+9 (19%), H-1->L+8 (24%)	H-4->L+9 (5%), H-3->L+4 (3%), H-3->L+5 (6%), H-3->L+6 (3%), H-3->L+7 (6%), H-3->L+8 (4%), H-2->L+8 (3%), H-2->L+9 (2%), H-1->L+7 (3%), H-1->L+9 (5%)
241.2	0.015	H-8->L+1 (22%), H-8->L+3 (16%), H-6->L+1 (41%), H-6->L+3 (14%)	H-8->L+2 (3%), H-6->L+2 (4%)
240.9	0.000	H-1->L+1 (100%)	
240.7	0.000	HOMO->L+24 (37%), HOMO->L+25 (31%)	HOMO->L+21 (4%), HOMO->L+23 (6%), HOMO->L+27 (4%), HOMO->L+29 (4%), HOMO->L+30 (6%)
238.4	0.000	HOMO->L+24 (11%), HOMO->L+27 (17%), HOMO->L+28 (33%)	H-2->L+7 (2%), HOMO->L+22 (5%), HOMO->L+23 (4%), HOMO->L+26 (5%), HOMO->L+32 (3%), HOMO->L+33 (7%)
237.3	0.012	H-7->L+5 (14%), H-2->L+4 (26%), H-2->L+5 (11%)	H-7->L+4 (7%), H-7->L+8 (2%), H-5->L+4 (9%), H-5->L+5 (8%), H-5->L+6 (4%), H-1->L+4 (3%), H-1->L+5 (7%)
235.6	0.000	H-1->L+2 (99%)	
234.7	0.001	H-1->L+8 (14%), H-1->L+9 (12%)	H-1->L+6 (3%), H-1->L+7 (7%), HOMO->L+25 (8%), HOMO->L+26 (6%), HOMO->L+27 (5%), HOMO->L+28 (6%), HOMO->L+29 (9%), HOMO->L+30 (4%), HOMO->L+32 (8%)
234.7	0.003	H-1->L+8 (19%), H-1->L+9 (16%)	H-4->L+8 (2%), H-1->L+6 (4%), H-1->L+7 (9%), HOMO->L+25 (6%), HOMO->L+26 (5%), HOMO->L+27 (4%), HOMO->L+28 (4%), HOMO->L+29 (6%), HOMO->L+30 (3%), HOMO->L+32 (6%)
233.9	0.001	HOMO->L+28 (26%), HOMO->L+33 (35%)	H-2->L+2 (2%), H-2->L+6 (2%), H-2->L+7 (4%), HOMO->L+23 (3%), HOMO->L+26 (3%), HOMO->L+29 (3%), HOMO->L+30 (3%), HOMO->L+35 (3%), HOMO->L+39 (2%)
232.2	0.001	H-2->L+1 (29%), HOMO->L+27 (26%), HOMO->L+32 (19%)	HOMO->L+25 (2%), HOMO->L+26 (5%), HOMO->L+29 (8%), HOMO->L+31 (3%)
232.0	0.000	H-2->L+1 (66%), HOMO->L+27 (13%)	H-4->L+1 (2%), HOMO->L+26 (4%), HOMO->L+29 (3%), HOMO->L+32 (6%)
230.9	0.008	H-2->L+2 (21%), HOMO->L+26 (15%), HOMO->L+30 (25%)	H-4->L+2 (2%), H-2->L+4 (6%), H-2->L+7 (3%), HOMO->L+31 (5%), HOMO->L+32 (4%), HOMO->L+38 (2%), HOMO->L+40 (2%)
230.7	0.010	H-4->L+4 (13%), H-2->L+4 (40%), H-2->L+6 (15%)	H-5->L+4 (4%), H-2->L+2 (2%), H-2->L+7 (2%), HOMO->L+26 (3%), HOMO->L+30 (5%)
229.7	0.005	H-2->L+2 (15%), H-1->L+8 (13%), H-1->L+9 (39%)	H-5->L+9 (2%), H-4->L+8 (4%), H-4->L+9 (3%), H-3->L+9 (2%), HOMO->L+30 (4%), HOMO->L+31 (4%)
229.7	0.009	H-2->L+2 (39%), H-1->L+9 (16%)	H-4->L+2 (4%), H-1->L+8 (5%), HOMO->L+30 (9%), HOMO->L+31 (9%), HOMO->L+33 (3%)
229.2	0.001	HOMO->L+31 (71%), HOMO->L+32 (12%)	H-2->L+2 (4%), HOMO->L+26 (3%), HOMO->L+30 (3%), HOMO->L+33 (2%)
228.2	0.000	H-3->L+1 (100%)	
227.7	0.041	H-11->LUMO (93%)	H-2->L+2 (2%)
226.8	0.001	HOMO->L+29 (40%), HOMO->L+30 (11%), HOMO->L+32 (21%)	HOMO->L+25 (2%), HOMO->L+28 (4%), HOMO->L+34 (8%), HOMO->L+37 (3%), HOMO->L+39 (3%)
226.0	0.000	H-4->L+1 (97%)	H-2->L+1 (3%)

225.2	0.000	H-1->L+3 (99%)	
224.6	0.051	H-2->L+5 (47%)	H-4->L+4 (3%), H-4->L+5 (4%), H-2->L+4 (3%), H-2->L+6 (4%), HOMO->L+29 (6%), HOMO->L+30 (3%), HOMO->L+33 (7%), HOMO->L+40 (3%), HOMO->L+42 (2%)
223.8	0.001	HOMO->L+29 (15%), HOMO->L+34 (17%), HOMO->L+36 (25%), HOMO->L+39 (14%)	HOMO->L+35 (5%), HOMO->L+37 (7%), HOMO->L+40 (3%)
223.6	0.026	H-2->L+5 (20%), HOMO->L+40 (15%)	H-4->L+4 (4%), H-2->L+7 (4%), HOMO->L+29 (3%), HOMO->L+30 (6%), HOMO->L+34 (6%), HOMO->L+35 (3%), HOMO->L+36 (3%), HOMO->L+38 (4%), HOMO->L+42 (8%), HOMO->L+44 (3%)
223.2	0.000	H-3->L+2 (99%)	
223.1	0.007	H-1->L+4 (14%), H-1->L+6 (60%)	H-1->L+8 (7%), H-1->L+9 (4%)
221.7	0.016	H-13->LUMO (93%)	H-12->LUMO (5%)
221.7	0.001	H-4->L+2 (83%)	H-2->L+2 (9%)
221.2	0.003	HOMO->L+33 (17%), HOMO->L+37 (12%)	H-4->L+2 (4%), H-4->L+4 (2%), H-2->L+6 (6%), H-2->L+7 (9%), H-1->L+7 (2%), HOMO->L+34 (3%), HOMO->L+35 (8%), HOMO->L+40 (6%), HOMO->L+42 (6%), HOMO->L+44 (3%), HOMO->L+46 (2%), HOMO->L+49 (2%)
221.1	0.007	H-6->L+1 (15%), H-6->L+2 (70%)	H-15->LUMO (2%), H-8->L+1 (9%), H-8->L+3 (2%)
220.3	0.087	H-4->L+4 (33%), H-4->L+5 (36%)	H-3->L+4 (6%), H-2->L+4 (6%)
219.9	0.001	H-1->L+7 (64%), H-1->L+8 (10%)	H-3->L+4 (3%), H-1->L+6 (9%), H-1->L+9 (2%)
219.8	0.002	H-3->L+4 (75%), H-3->L+5 (11%)	H-4->L+4 (4%), H-3->L+6 (2%), H-3->L+8 (3%)
219.4	0.018	H-12->LUMO (42%), H-8->L+1 (11%), H-6->L+2 (10%), H-6->L+3 (10%)	H-15->LUMO (9%), H-13->LUMO (4%), H-8->L+2 (3%), H-6->L+1 (6%)
218.8	0.011	H-4->L+4 (13%), H-2->L+6 (36%)	H-4->L+5 (6%), H-4->L+6 (7%), H-2->L+4 (3%), H-2->L+7 (5%), H-2->L+8 (5%), HOMO->L+34 (4%), HOMO->L+36 (7%)
218.3	0.004	HOMO->L+34 (26%), HOMO->L+36 (32%)	H-4->L+4 (3%), H-2->L+6 (3%), H-2->L+7 (5%), HOMO->L+32 (4%), HOMO->L+38 (3%), HOMO->L+39 (4%), HOMO->L+42 (3%)
218.1	0.099	H-15->LUMO (77%), H-12->LUMO (13%)	
218.0	0.000	H-3->L+5 (76%)	H-3->L+4 (6%), H-3->L+6 (2%), H-3->L+8 (3%), H-3->L+9 (5%)
217.5	0.001	H-2->L+3 (92%)	H-4->L+3 (3%)
216.9	0.076	H-12->LUMO (29%), H-8->L+1 (17%), H-6->L+1 (13%), H-6->L+2 (11%), H-6->L+3 (17%)	H-15->LUMO (6%), H-8->L+3 (3%)
216.5	0.000	HOMO->L+34 (26%), HOMO->L+35 (27%), HOMO->L+39 (20%)	HOMO->L+33 (5%), HOMO->L+36 (9%), HOMO->L+37 (6%)
216.3	0.000	H-5->L+1 (99%)	
216.0	0.011	H-5->L+5 (10%), H-4->L+5 (23%)	H-7->L+5 (2%), H-5->L+4 (3%), H-5->L+6 (6%), H-4->L+4 (7%), H-4->L+7 (2%), H-3->L+8 (2%), H-2->L+4 (6%), H-2->L+5 (5%), H-2->L+7 (6%), H-2->L+8 (3%), HOMO->L+35 (5%), HOMO->L+38 (3%)
215.8	0.001	HOMO->L+35 (26%)	H-5->L+4 (6%), H-5->L+5 (4%), H-4->L+4 (5%), H-4->L+5 (7%), H-2->L+6 (5%), H-2->L+7 (8%), HOMO->L+37 (3%), HOMO->L+38 (8%), HOMO->L+39 (4%), HOMO->L+40 (4%), HOMO->L+42 (6%)
214.8	0.005	HOMO->L+35 (11%), HOMO->L+39 (24%)	H-10->L+4 (2%), H-5->L+4 (5%), H-4->L+6 (3%), H-2->L+6 (5%), H-2->L+7 (7%), HOMO->L+37 (9%), HOMO->L+38 (5%), HOMO->L+40 (3%), HOMO->L+42 (4%), HOMO->L+46 (6%)
214.4	0.029	H-5->L+4 (18%), H-3->L+8 (18%)	H-7->L+4 (3%), H-5->L+5 (4%), H-4->L+5 (3%), H-4->L+6 (6%), H-4->L+7 (4%), H-4->L+9 (8%), H-3->L+7 (3%), H-3->L+9 (7%), H-2->L+7 (9%)
214.0	0.000	H-3->L+3 (99%)	
213.0	0.005	H-8->L+2 (87%)	H-12->LUMO (4%), H-8->L+3 (6%)
212.6	0.001	H-5->L+2 (72%), H-4->L+3 (16%)	
212.4	0.000	HOMO->L+37 (34%), HOMO->L+38 (50%)	HOMO->L+39 (3%), HOMO->L+40 (4%), HOMO->L+42 (3%), HOMO->L+46 (2%)

212.2	0.021	H-5->L+4 (16%), H-5->L+5 (13%), H-4->L+3 (32%)	H-7->L+4 (2%), H-5->L+2 (8%), H-4->L+8 (2%), H-4->L+9 (4%), H-3->L+8 (3%), H-2->L+8 (5%), H-2->L+9 (2%)
212.0	0.015	H-5->L+2 (10%), H-5->L+4 (11%), H-4->L+3 (47%)	H-5->L+5 (9%), H-4->L+9 (3%), H-3->L+8 (2%), H-2->L+8 (4%)
211.0	0.000	H-14->LUMO (100%)	
211.0	0.016	H-5->L+5 (14%), H-4->L+6 (26%)	H-7->L+5 (3%), H-5->L+2 (3%), H-5->L+6 (7%), H-2->L+6 (4%), HOMO->L+37 (5%), HOMO->L+39 (4%), HOMO->L+44 (9%), HOMO->L+47 (3%), HOMO->L+49 (2%)
210.2	0.002	HOMO->L+43 (24%), HOMO->L+46 (25%), HOMO->L+47 (22%)	HOMO->L+37 (2%), HOMO->L+38 (5%), HOMO->L+41 (4%), HOMO->L+42 (5%), HOMO->L+50 (3%)
209.5	0.013	H-5->L+7 (16%), H-4->L+7 (24%), H-4->L+8 (10%), H-2->L+7 (14%)	H-5->L+2 (2%), H-4->L+9 (3%), H-3->L+8 (2%), H-2->L+10 (2%), HOMO->L+41 (6%), HOMO->L+44 (4%)
209.2	0.001	H-4->L+6 (11%), H-4->L+7 (11%), HOMO->L+41 (28%), HOMO->L+44 (15%)	H-10->L+4 (4%), H-5->L+7 (4%), HOMO->L+36 (3%), HOMO->L+40 (2%), HOMO->L+42 (4%), HOMO->L+48 (3%)
208.5	0.001	HOMO->L+40 (38%), HOMO->L+42 (13%), HOMO->L+44 (15%)	HOMO->L+36 (3%), HOMO->L+43 (7%), HOMO->L+45 (5%), HOMO->L+46 (5%), HOMO->L+47 (3%)
208.5	0.000	H-7->L+1 (100%)	
208.4	0.003	HOMO->L+41 (32%)	H-3->L+6 (8%), H-3->L+8 (2%), H-2->L+8 (3%), HOMO->L+39 (5%), HOMO->L+40 (8%), HOMO->L+42 (4%), HOMO->L+43 (3%), HOMO->L+44 (4%), HOMO->L+46 (6%), HOMO->L+47 (2%), HOMO->L+48 (4%), HOMO->L+49 (5%)
208.3	0.001	H-3->L+6 (74%)	H-3->L+4 (3%), H-3->L+8 (7%), HOMO->L+41 (4%)

Table S3.b: TD-DFT[C-PCM(CH_2Cl_2)/CAM-B3LYP(D3)/6-311G**] singlet electronic excitations for the investigated Am-Bpy-Trz$^+$ molecular thread. The derived UV/Vis absorption spectrum is shown in the associated manuscript in Fig 1 (right lower panel).

Wavelength (nm)	Osc, Strength	Major contribs	Minor contribs
485.1	0.0068	HOMO->LUMO (99%)	
463.0	0.0009	HOMO->L+4 (41%), HOMO->L+7 (30%), HOMO->L+8 (22%)	HOMO->L+9 (3%)
373.5	0.0309	HOMO->L+11 (76%)	HOMO->L+4 (8%), HOMO->L+7 (2%), HOMO->L+8 (6%), HOMO->L+9 (3%)
361.0	1.5766	HOMO->L+3 (26%), HOMO->L+5 (35%), HOMO->L+6 (29%)	HOMO->L+2 (6%), HOMO->L+11 (2%)
317.3	0.0075	HOMO->L+5 (29%), HOMO->L+6 (54%)	HOMO->L+3 (2%), HOMO->L+4 (3%), HOMO->L+7 (3%), HOMO->L+9 (3%)
299.9	0.0009	HOMO->L+4 (43%), HOMO->L+7 (13%), HOMO->L+8 (17%), HOMO->L+11 (17%)	HOMO->L+6 (3%), HOMO->L+9 (4%)
285.8	0.0021	HOMO->L+2 (73%), HOMO->L+5 (14%)	HOMO->L+3 (6%), HOMO->L+6 (5%)
282.8	0	HOMO->L+1 (100%)	
267.8	0.0074	HOMO->L+10 (10%), HOMO->L+14 (42%), HOMO->L+18 (13%)	HOMO->L+12 (4%), HOMO->L+13 (3%), HOMO->L+17 (5%), HOMO->L+19 (9%), HOMO->L+23 (4%)
260.0	0.0001	HOMO->L+2 (19%), HOMO->L+3 (64%), HOMO->L+5 (14%)	
251.4	0.018	H-6->LUMO (64%)	H-8->LUMO (6%), H-6->L+1 (4%), HOMO->L+7 (4%), HOMO->L+8 (2%), HOMO->L+10 (3%), HOMO->L+18 (4%)
251.3	0.0006	H-6->LUMO (15%), HOMO->L+7 (16%), HOMO->L+8 (10%), HOMO->L+10 (13%), HOMO->L+18 (15%)	HOMO->L+6 (2%), HOMO->L+9 (3%), HOMO->L+12 (3%), HOMO->L+16 (5%), HOMO->L+21 (2%)
249.7	0.0012	HOMO->L+7 (30%), HOMO->L+8 (24%), HOMO->L+10 (12%), HOMO->L+18 (14%)	HOMO->L+12 (2%), HOMO->L+16 (3%)
246.2	0.0013	HOMO->L+15 (13%), HOMO->L+21 (45%)	HOMO->L+13 (8%), HOMO->L+18 (3%), HOMO->L+24 (7%), HOMO->L+25 (4%), HOMO->L+26 (3%), HOMO->L+29 (3%)
244.3	0.0001	HOMO->L+8 (17%), HOMO->L+9 (80%)	

241.6	0.0682	H-8->LUMO (81%)	H-8->L+1 (5%), H-6->LUMO (3%), H-6->L+1 (3%)
234.6	0.0024	H-3->L+7 (15%), H-3->L+9 (26%)	H-5->L+8 (3%), H-4->L+7 (4%), H-4->L+8 (7%), H-4->L+9 (3%), H-3->L+4 (3%), H-3->L+6 (3%), H-2->L+7 (2%), H-2->L+8 (5%), H-2->L+9 (4%), H-1->L+7 (5%), H-1->L+8 (9%), H-1->L+9 (3%)
233.8	0.0016	HOMO->L+10 (37%), HOMO->L+18 (13%)	HOMO->L+14 (5%), HOMO->L+16 (5%), HOMO->L+19 (6%), HOMO->L+20 (8%), HOMO->L+23 (2%), HOMO->L+24 (7%), HOMO->L+25 (6%)
232.9	0.0071	H-7->L+4 (11%)	H-7->L+5 (7%), H-7->L+6 (8%), H-7->L+8 (3%), H-7->L+9 (3%), H-5->L+4 (8%), H-5->L+5 (2%), H-5->L+6 (4%), H-5->L+7 (2%), H-4->L+5 (2%), H-4->L+6 (4%), H-2->L+4 (8%), H-2->L+5 (4%), H-2->L+6 (7%), H-2->L+7 (2%), H-1->L+4 (3%), H-1->L+5 (3%), H-1->L+6 (4%)
230.2	0.003	H-8->L+1 (15%), H-6->LUMO (17%), H-6->L+1 (32%)	H-8->LUMO (6%), H-8->L+2 (7%), H-8->L+3 (8%), H-6->L+2 (4%), H-6->L+3 (7%)
224.3	0.0003	HOMO->L+13 (11%), HOMO->L+14 (10%), HOMO->L+19 (19%), HOMO->L+23 (13%)	HOMO->L+10 (3%), HOMO->L+20 (7%), HOMO->L+24 (5%), HOMO->L+27 (2%), HOMO->L+29 (5%), HOMO->L+30 (5%), HOMO->L+31 (2%)
223.3	0.0004	HOMO->L+15 (20%)	HOMO->L+10 (8%), HOMO->L+12 (2%), HOMO->L+13 (8%), HOMO->L+14 (7%), HOMO->L+16 (4%), HOMO->L+17 (2%), HOMO->L+19 (4%), HOMO->L+20 (4%), HOMO->L+24 (5%), HOMO->L+26 (8%), HOMO->L+28 (8%), HOMO->L+29 (6%), HOMO->L+33 (3%)
217.4	0.077	H-12->LUMO (12%), H-11->LUMO (27%), H-4->LUMO (21%), H-2->LUMO (33%)	
212.8	0.0062		HOMO->L+10 (5%), HOMO->L+12 (4%), HOMO->L+13 (4%), HOMO->L+16 (8%), HOMO->L+17 (3%), HOMO->L+19 (5%), HOMO->L+20 (3%), HOMO->L+21 (3%), HOMO->L+22 (8%), HOMO->L+23 (5%), HOMO->L+24 (3%), HOMO->L+25 (7%), HOMO->L+26 (5%), HOMO->L+27 (3%), HOMO->L+28 (2%), HOMO->L+32 (3%), HOMO->L+33 (4%), HOMO->L+34 (3%), HOMO->L+39 (6%), HOMO->L+40 (4%), HOMO->L+44 (2%)
212.1	0.0264	H-12->LUMO (12%), H-11->LUMO (12%), H-8->L+1 (15%), H-6->L+1 (16%), H-2->LUMO (18%)	H-6->L+2 (4%), H-6->L+3 (6%), H-4->LUMO (8%)
211.7	0.0562	H-1->L+4 (23%)	H-7->L+4 (2%), H-7->L+6 (2%), H-4->L+4 (3%), H-4->L+9 (3%), H-3->L+7 (4%), H-3->L+8 (6%), H-3->L+9 (2%), H-2->L+4 (2%), H-2->L+7 (2%), H-2->L+9 (4%), H-1->L+5 (8%), H-1->L+6 (9%), H-1->L+7 (5%), H-1->L+9 (2%)
210.8	0.0989	H-3->L+8 (14%), H-1->L+9 (12%)	H-7->L+4 (2%), H-5->L+9 (3%), H-4->L+4 (4%), H-4->L+6 (3%), H-4->L+7 (3%), H-3->L+7 (8%), H-3->L+9 (5%), H-2->L+4 (8%), H-2->L+5 (5%), H-2->L+6 (4%), H-2->L+7 (2%), H-1->L+7 (5%)
210.5	0.0968	H-12->LUMO (14%), H-11->LUMO (17%), H-8->L+1 (17%), H-6->L+1 (16%)	H-8->L+3 (2%), H-6->L+2 (5%), H-6->L+3 (6%), H-4->LUMO (3%), H-2->LUMO (9%)
209.4	0.0017	HOMO->L+12 (58%), HOMO->L+13 (10%)	HOMO->L+10 (3%), HOMO->L+15 (3%), HOMO->L+18 (2%), HOMO->L+23 (4%), HOMO->L+27 (2%)
207.4	0.0002	HOMO->L+16 (10%)	HOMO->L+12 (4%), HOMO->L+15 (7%), HOMO->L+18 (9%), HOMO->L+20 (5%), HOMO->L+22 (9%), HOMO->L+23 (6%), HOMO->L+26 (3%), HOMO->L+27 (9%), HOMO->L+28 (6%), HOMO->L+29 (5%)
206.5	0.0002	H-1->LUMO (96%)	
206.2	0.0051	HOMO->L+16 (14%), HOMO->L+23 (10%), HOMO->L+25 (13%)	HOMO->L+12 (6%), HOMO->L+13 (9%), HOMO->L+15 (3%), HOMO->L+20 (3%), HOMO->L+22 (5%), HOMO->L+24 (4%), HOMO->L+30 (3%), HOMO->L+34 (6%), HOMO->L+37 (3%)
204.2	0.0089	H-4->L+5 (10%), H-2->L+5 (12%)	H-5->L+6 (2%), H-4->L+3 (6%), H-4->L+6 (6%), H-2->L+3 (7%), H-2->L+6 (7%), HOMO->L+12 (4%), HOMO->L+13 (5%), HOMO->L+16 (6%), HOMO->L+33 (3%), HOMO->L+39 (2%)
203.6	0.0483	H-5->L+4 (11%), H-1->L+4 (11%)	H-7->L+4 (3%), H-5->L+5 (6%), H-5->L+6 (6%), H-5->L+7 (3%), H-4->L+4 (3%), H-4->L+9 (2%), H-3->L+8 (5%), H-3->L+9 (3%), H-2->L+4 (7%), H-2->L+7 (2%), H-1->L+5 (2%), H-1->L+6 (2%), H-1->L+7 (5%)
202.4	0.0044	H-4->L+4 (11%), H-2->L+4 (12%)	H-4->L+7 (8%), H-4->L+8 (5%), H-2->L+7 (9%), H-2->L+8 (7%), HOMO->L+12 (3%), HOMO->L+13

			(4%), HOMO->L+16 (3%), HOMO->L+33 (3%), HOMO->L+39 (3%)
202.1	0	H-3->LUMO (95%)	H-2->LUMO (3%)
201.4	0.003	H-4->LUMO (34%), H-2->LUMO (20%)	H-4->L+4 (2%), H-4->L+6 (2%), H-3->LUMO (3%), H-2->L+6 (3%), HOMO->L+13 (2%)
201.3	0.0032	H-4->LUMO (27%), H-2->LUMO (13%)	H-4->L+4 (2%), H-4->L+6 (4%), H-2->L+3 (2%), H-2->L+6 (5%), HOMO->L+12 (2%), HOMO->L+13 (4%), HOMO->L+33 (2%)
198.7	0.0005	HOMO->L+13 (24%), HOMO->L+15 (14%), HOMO->L+42 (11%)	HOMO->L+14 (4%), HOMO->L+16 (4%), HOMO->L+21 (2%), HOMO->L+25 (3%), HOMO->L+33 (2%), HOMO->L+35 (8%), HOMO->L+40 (4%), HOMO->L+43 (3%)
196.0	0.0155	H-1->L+4 (13%), H-1->L+5 (17%), H-1->L+6 (26%)	H-7->L+4 (2%), H-5->L+4 (5%), H-5->L+5 (4%), H-5->L+6 (7%), H-4->L+6 (2%), H-1->L+7 (3%), H-1->L+8 (4%)
194.6	0.0102	HOMO->L+17 (11%)	HOMO->L+12 (3%), HOMO->L+14 (9%), HOMO->L+16 (7%), HOMO->L+18 (3%), HOMO->L+20 (9%), HOMO->L+25 (3%), HOMO->L+27 (3%), HOMO->L+30 (2%), HOMO->L+39 (2%), HOMO->L+40 (9%), HOMO->L+42 (6%), HOMO->L+43 (3%), HOMO->L+44 (9%)
193.8	0.0079	HOMO->L+15 (17%), HOMO->L+20 (13%)	HOMO->L+16 (8%), HOMO->L+18 (3%), HOMO->L+21 (6%), HOMO->L+22 (6%), HOMO->L+27 (9%), HOMO->L+40 (2%), HOMO->L+41 (3%), HOMO->L+46 (6%)
193.4	0.155	H-1->L+7 (22%), H-1->L+8 (13%), H-1->L+9 (20%)	H-7->L+4 (2%), H-5->L+7 (4%), H-5->L+8 (3%), H-5->L+9 (5%), H-3->L+8 (5%), H-3->L+9 (8%)
191.9	0.0581	HOMO->L+19 (35%)	HOMO->L+15 (6%), HOMO->L+20 (5%), HOMO->L+22 (2%), HOMO->L+27 (2%), HOMO->L+29 (4%), HOMO->L+30 (4%), HOMO->L+32 (2%), HOMO->L+40 (3%), HOMO->L+44 (4%), HOMO->L+49 (4%)
191.8	0.6728	H-8->L+1 (25%), H-6->L+2 (15%), H-6->L+3 (20%)	H-26->LUMO (6%), H-23->LUMO (9%), H-15->LUMO (5%), H-6->L+5 (3%), H-5->LUMO (5%)
191.5	0.0381	H-5->LUMO (90%)	H-4->LUMO (3%)
190.9	0.5284	H-3->L+9 (12%), H-1->L+8 (20%), H-1->L+9 (35%)	H-3->L+4 (2%), H-3->L+6 (2%), H-3->L+7 (9%), H-2->L+8 (2%), H-1->L+7 (5%)
189.4	0.0001	HOMO->L+17 (58%)	HOMO->L+14 (2%), HOMO->L+15 (7%), HOMO->L+16 (7%), HOMO->L+18 (3%), HOMO->L+21 (2%), HOMO->L+40 (3%), HOMO->L+42 (4%)
189.0	0.0372	HOMO->L+20 (28%)	HOMO->L+16 (6%), HOMO->L+17 (2%), HOMO->L+18 (4%), HOMO->L+19 (5%), HOMO->L+21 (5%), HOMO->L+22 (3%), HOMO->L+27 (6%), HOMO->L+35 (2%), HOMO->L+37 (2%), HOMO->L+46 (3%), HOMO->L+49 (4%), HOMO->L+51 (3%)
188.4	0.6975		H-7->L+4 (4%), H-7->L+5 (4%), H-7->L+6 (7%), H-5->L+7 (6%), H-5->L+9 (4%), H-4->L+7 (4%), H-4->L+8 (3%), H-4->L+9 (6%), H-3->L+7 (3%), H-3->L+8 (9%), H-3->L+9 (7%), H-2->L+4 (7%), H-2->L+7 (3%)
188.1	0.0445	H-10->LUMO (23%), H-9->LUMO (59%)	H-7->LUMO (6%)
187.4	0.8306	H-8->L+2 (23%), H-8->L+3 (28%), H-6->L+1 (20%)	H-26->LUMO (3%), H-23->LUMO (4%), H-15->LUMO (2%), H-8->L+5 (4%), H-6->L+2 (2%)
187.3	0.2386	H-9->L+5 (13%), H-9->L+6 (12%), H-4->L+11 (11%), H-2->L+11 (14%)	H-10->LUMO (3%), H-9->LUMO (4%), H-9->L+3 (9%), H-4->L+7 (3%)
186.7	0.0151	HOMO->L+23 (12%), HOMO->L+45 (17%)	HOMO->L+17 (8%), HOMO->L+24 (2%), HOMO->L+28 (4%), HOMO->L+30 (4%), HOMO->L+32 (2%), HOMO->L+33 (3%), HOMO->L+35 (3%), HOMO->L+40 (5%), HOMO->L+42 (4%), HOMO->L+43 (2%), HOMO->L+49 (3%), HOMO->L+52 (2%), HOMO->L+54 (3%), HOMO->L+55 (2%)
186.5	0.0005	H-7->LUMO (93%)	H-10->LUMO (3%), H-9->LUMO (4%)
184.7	1.1147	H-7->L+5 (17%), H-7->L+6 (17%), H-5->L+4 (14%)	H-7->L+3 (2%), H-5->L+7 (2%), H-4->L+7 (3%), H-3->L+8 (4%), H-3->L+9 (4%), H-2->L+4 (7%), H-2->L+9 (2%)
184.3	0.0983	H-26->LUMO (11%), H-23->LUMO (18%), H-15->LUMO (12%), H-8->L+1 (11%)	H-13->LUMO (3%), H-11->L+2 (2%), H-8->L+2 (5%), H-8->L+3 (7%), H-6->L+1 (4%), H-6->L+2 (5%), H-6->L+3 (3%)
184.2	0.6461	H-7->L+4 (14%), H-5->L+9 (10%), H-4->L+9 (12%)	H-7->L+9 (2%), H-5->L+7 (2%), H-5->L+8 (6%), H-4->L+8 (7%), H-3->L+7 (5%), H-3->L+8 (2%), H-2->L+6 (3%), H-2->L+9 (5%), H-1->L+7 (2%), H-1->L+8 (3%), H-1->L+9 (3%)

WILEY

183.5	0.1588	H-7->L+4 (26%)	H-7->L+8 (6%), H-5->L+5 (4%), H-5->L+6 (4%), H-5->L+8 (5%), H-5->L+9 (4%), H-4->L+7 (3%), H-4->L+8 (4%), H-4->L+9 (4%), H-3->L+9 (6%), H-2->L+5 (4%), H-2->L+6 (6%), H-2->L+7 (5%)
182.0	0.0066	HOMO->L+23 (12%), HOMO->L+24 (10%), HOMO->L+37 (10%)	HOMO->L+19 (3%), HOMO->L+22 (7%), HOMO->L+25 (6%), HOMO->L+38 (5%), HOMO->L+39 (2%), HOMO->L+47 (2%), HOMO->L+50 (2%), HOMO->L+52 (2%), HOMO->L+53 (4%), HOMO->L+58 (3%)
181.3	0.0305	H-49->LUMO (14%), H-48->LUMO (43%)	H-43->LUMO (2%), H-41->LUMO (2%), H-39->LUMO (8%), H-36->LUMO (5%), H-33->LUMO (5%), H-29->LUMO (2%)
180.5	0.0021	HOMO->L+22 (48%), HOMO->L+27 (13%)	HOMO->L+18 (3%), HOMO->L+24 (3%), HOMO->L+29 (2%), HOMO->L+41 (4%), HOMO->L+46 (2%), HOMO->L+47 (6%), HOMO->L+49 (2%)
178.1	0.0016	HOMO->L+21 (17%), HOMO->L+24 (28%), HOMO->L+25 (23%)	HOMO->L+32 (7%), HOMO->L+43 (2%)
178.0	0.004	H-10->LUMO (51%), H-9->LUMO (22%)	H-10->L+3 (4%), H-10->L+5 (5%), H-10->L+6 (4%)
177.4	0.0305	H-10->LUMO (18%), H-10->L+5 (10%)	H-10->L+3 (7%), H-10->L+6 (9%), H-9->LUMO (7%), H-9->L+4 (2%), H-7->L+6 (2%), H-5->L+5 (2%), H-5->L+6 (3%), H-4->L+5 (2%), H-2->L+11 (2%)
176.7	0.0081	HOMO->L+29 (11%), HOMO->L+33 (11%)	HOMO->L+23 (2%), HOMO->L+24 (5%), HOMO->L+26 (4%), HOMO->L+30 (3%), HOMO->L+31 (4%), HOMO->L+32 (5%), HOMO->L+44 (5%), HOMO->L+48 (4%), HOMO->L+49 (3%), HOMO->L+51 (4%)
176.4	0.0632	H-1->L+16 (18%), H-1->L+20 (11%)	H-10->L+5 (2%), H-10->L+6 (2%), H-5->L+16 (3%), H-5->L+20 (2%), H-1->L+15 (5%), H-1->L+19 (6%), H-1->L+30 (3%), H-1->L+31 (5%)
176.3	0.0558	HOMO->L+30 (16%), HOMO->L+31 (28%)	H-9->L+4 (2%), H-4->L+6 (3%), HOMO->L+32 (2%), HOMO->L+55 (5%), HOMO->L+61 (4%)
176.1	0.0099	HOMO->L+30 (15%)	HOMO->L+23 (5%), HOMO->L+26 (3%), HOMO->L+31 (2%), HOMO->L+51 (4%), HOMO->L+52 (3%), HOMO->L+53 (4%), HOMO->L+54 (9%), HOMO->L+55 (9%), HOMO->L+56 (4%), HOMO->L+58 (6%), HOMO->L+61 (5%), HOMO->L+63 (5%)
175.7	0.4475	H-9->L+4 (21%), H-9->L+7 (12%)	H-10->L+6 (2%), H-10->L+11 (8%), H-9->L+8 (9%), HOMO->L+31 (4%), HOMO->L+55 (2%)
174.6	0.1688		H-10->L+3 (4%), H-10->L+5 (6%), H-10->L+6 (6%), H-9->L+3 (6%), H-9->L+5 (8%), H-9->L+6 (9%), H-4->L+4 (3%), H-4->L+5 (2%), H-4->L+8 (4%), H-4->L+9 (2%), H-4->L+11 (5%), H-2->L+4 (2%), H-2->L+8 (3%), H-2->L+11 (8%), HOMO->L+30 (3%), HOMO->L+31 (4%)
174.1	0.0939	H-4->L+6 (20%)	H-5->L+5 (2%), H-5->L+6 (7%), H-4->L+5 (9%), H-4->L+7 (4%), H-2->L+5 (7%), H-2->L+6 (6%), H-2->L+11 (2%), HOMO->L+31 (8%)
173.6	0.0061	HOMO->L+26 (14%), HOMO->L+27 (10%), HOMO->L+45 (14%)	HOMO->L+24 (3%), HOMO->L+28 (3%), HOMO->L+32 (8%), HOMO->L+46 (7%), HOMO->L+51 (2%), HOMO->L+62 (5%), HOMO->L+63 (4%)
173.5	0.004	H-13->LUMO (37%), H-13->L+1 (21%), H-6->L+2 (13%)	H-6->L+3 (5%), H-6->L+5 (4%)
173.4	0.0043	H-13->LUMO (13%), H-6->L+2 (35%), H-6->L+3 (15%), H-6->L+5 (11%)	H-15->LUMO (3%), H-13->L+1 (8%), H-8->L+2 (3%), H-6->L+6 (2%)
173.1	0.0105	HOMO->L+31 (14%), HOMO->L+52 (10%)	HOMO->L+23 (6%), HOMO->L+25 (8%), HOMO->L+26 (5%), HOMO->L+27 (6%), HOMO->L+29 (5%), HOMO->L+45 (3%), HOMO->L+48 (5%), HOMO->L+51 (3%), HOMO->L+55 (3%), HOMO->L+63 (4%)
172.1	0.008	HOMO->L+27 (13%), HOMO->L+31 (11%)	HOMO->L+24 (5%), HOMO->L+25 (2%), HOMO->L+26 (4%), HOMO->L+30 (6%), HOMO->L+32 (6%), HOMO->L+36 (2%), HOMO->L+37 (2%), HOMO->L+44 (2%), HOMO->L+47 (9%), HOMO->L+49 (3%), HOMO->L+51 (2%), HOMO->L+63 (4%)
171.9	0.0593	H-4->L+4 (10%)	H-10->L+3 (2%), H-10->L+5 (3%), H-10->L+6 (2%), H-9->L+4 (2%), H-9->L+5 (3%), H-5->L+4 (4%), H-5->L+5 (3%), H-4->L+3 (2%), H-4->L+5 (6%), H-2->L+6 (7%), H-2->L+7 (4%), H-2->L+8 (3%), H-1->L+3 (2%), H-1->L+5 (3%), H-1->L+6 (4%)
170.7	0.0115	HOMO->L+26 (36%), HOMO->L+28 (23%)	HOMO->L+29 (7%), HOMO->L+32 (5%), HOMO->L+33 (4%), HOMO->L+52 (3%)

170.0	0.0028	HOMO->L+32 (16%)	HOMO->L+25 (7%), HOMO->L+26 (4%), HOMO->L+28 (4%), HOMO->L+34 (6%), HOMO->L+35 (7%), HOMO->L+39 (2%), HOMO->L+46 (2%), HOMO->L+52 (2%), HOMO->L+53 (4%), HOMO->L+55 (8%), HOMO->L+61 (9%)
169.9	0.0153	H-8->L+2 (48%), H-8->L+3 (19%), H-8->L+5 (14%)	H-8->L+6 (3%), H-6->L+2 (4%), H-6->L+3 (2%)
169.4	0.0729	H-15->LUMO (25%), H-15->L+1 (22%)	H-26->LUMO (8%), H-19->LUMO (2%), H-13->LUMO (2%), H-12->L+2 (3%), H-11->L+2 (6%)
169.0	0.0135		H-2->L+11 (3%), HOMO->L+27 (5%), HOMO->L+28 (7%), HOMO->L+29 (2%), HOMO->L+30 (6%), HOMO->L+31 (4%), HOMO->L+32 (5%), HOMO->L+34 (4%), HOMO->L+37 (3%), HOMO->L+41 (2%), HOMO->L+44 (3%), HOMO->L+55 (2%), HOMO->L+56 (3%), HOMO->L+61 (3%), HOMO->L+62 (3%)
168.9	0.0479		H-5->L+6 (2%), H-4->L+4 (5%), H-4->L+6 (3%), H-2->L+6 (2%), H-2->L+7 (5%), H-2->L+8 (5%), H-2->L+11 (9%), H-1->L+6 (2%), HOMO->L+28 (3%), HOMO->L+29 (2%), HOMO->L+30 (2%), HOMO->L+31 (3%), HOMO->L+32 (4%), HOMO->L+40 (2%)
168.4	0.0012	HOMO->L+28 (25%), HOMO->L+29 (29%)	HOMO->L+27 (3%), HOMO->L+30 (5%), HOMO->L+32 (5%), HOMO->L+35 (5%), HOMO->L+61 (3%)
167.6	0.1239	H-4->L+11 (19%)	H-5->L+7 (4%), H-5->L+8 (3%), H-5->L+11 (5%), H-4->L+4 (4%), H-4->L+7 (8%), H-4->L+8 (9%), H-4->L+9 (2%), H-2->L+4 (7%), H-2->L+11 (4%), H-1->L+4 (4%)
166.6	0.0063	HOMO->L+59 (13%)	H-10->L+4 (5%), H-10->L+7 (2%), HOMO->L+32 (5%), HOMO->L+33 (5%), HOMO->L+36 (4%), HOMO->L+40 (5%), HOMO->L+56 (2%), HOMO->L+60 (6%), HOMO->L+61 (4%), HOMO->L+62 (2%), HOMO->L+63 (2%), HOMO->L+67 (3%)
166.4	0.0105	HOMO->L+55 (18%)	HOMO->L+32 (4%), HOMO->L+34 (7%), HOMO->L+35 (2%), HOMO->L+36 (7%), HOMO->L+45 (6%), HOMO->L+46 (4%), HOMO->L+47 (2%), HOMO->L+49 (3%), HOMO->L+58 (8%), HOMO->L+59 (6%), HOMO->L+60 (4%), HOMO->L+61 (3%)
166.3	0.003	H-3->L+4 (66%)	H-5->L+4 (2%), H-4->L+4 (2%), H-3->L+8 (9%), H-3->L+9 (7%), H-1->L+4 (2%)
165.9	0.0053	H-13->LUMO (18%), H-13->L+1 (42%), H-13->L+2 (10%), H-13->L+3 (12%)	H-19->L+1 (2%)
165.6	0.1322	H-15->L+1 (11%), H-4->L+2 (14%), H-2->L+2 (18%)	H-11->L+2 (7%), H-4->L+3 (3%), H-2->L+4 (2%), H-2->L+5 (3%)
165.6	0.005	H-3->L+5 (33%), H-3->L+6 (34%)	H-3->L+3 (4%), H-3->L+7 (7%), H-3->L+9 (3%)
165.5	0.0087	H-5->L+4 (14%), H-4->L+4 (13%), H-2->L+4 (10%), H-1->L+4 (10%)	H-5->L+8 (3%), H-5->L+9 (2%), H-4->L+8 (2%), H-4->L+9 (3%), H-3->L+4 (7%), H-3->L+6 (3%), H-2->L+2 (2%), H-2->L+9 (3%)
164.7	0.0038	H-5->L+6 (14%), H-4->L+5 (10%)	H-5->L+5 (9%), H-5->L+7 (2%), H-4->L+6 (9%), H-4->L+7 (3%), H-3->L+5 (4%), H-3->L+6 (4%), H-2->L+5 (7%), H-2->L+6 (7%), H-2->L+7 (2%), H-1->L+5 (7%), H-1->L+6 (6%)
164.3	0.0034	H-10->L+4 (18%)	H-10->L+7 (7%), H-10->L+8 (5%), H-9->L+11 (3%), H-4->L+6 (2%), HOMO->L+33 (6%), HOMO->L+38 (5%), HOMO->L+39 (6%), HOMO->L+42 (2%), HOMO->L+46 (3%), HOMO->L+59 (3%)
163.2	0.0011	H-4->L+1 (18%), H-2->L+1 (57%)	H-15->L+1 (2%), H-1->L+1 (8%)
163.1	0.0071	H-15->LUMO (11%), H-15->L+1 (24%)	H-26->LUMO (2%), H-15->L+2 (5%), H-15->L+3 (6%), H-4->L+2 (2%), H-2->L+1 (4%), H-2->L+2 (4%), HOMO->L+35 (2%), HOMO->L+37 (2%), HOMO->L+38 (2%)
163.0	0.001	HOMO->L+35 (16%), HOMO->L+37 (14%)	H-15->L+1 (3%), H-10->L+4 (4%), H-2->L+1 (5%), HOMO->L+32 (2%), HOMO->L+33 (5%), HOMO->L+36 (4%), HOMO->L+39 (2%), HOMO->L+41 (3%), HOMO->L+44 (3%), HOMO->L+49 (4%)
162.8	0.0005	HOMO->L+34 (13%), HOMO->L+38 (22%)	HOMO->L+33 (6%), HOMO->L+35 (3%), HOMO->L+37 (4%), HOMO->L+41 (6%), HOMO->L+49 (2%), HOMO->L+62 (4%), HOMO->L+67 (4%)
162.2	0.0112	H-7->L+6 (12%), H-1->L+6 (11%)	H-7->L+3 (7%), H-7->L+5 (9%), H-5->L+3 (3%), H-5->L+5 (4%), H-5->L+6 (3%), H-1->L+2 (4%), H-1->L+3 (7%), H-1->L+5 (7%), HOMO->L+34 (4%), HOMO->L+35 (3%)

Table S4. Electron density $\rho(\boldsymbol{r})$ ($e \cdot a_0^{-3}$), Laplacian of electron density $\nabla^2\rho(\boldsymbol{r})$ ($e \cdot a_0^{-5}$), electron kinetic energy density $G(\boldsymbol{r})$ (hartree$\cdot a_0^{-3}$), electron potential energy density $V(\boldsymbol{r})$ (hartree$\cdot a_0^{-3}$), and electron energy density $H(\mathbf{r})$ (hartree$\cdot a_0^{-3}$) for bond critical points on selected bonds of the Am-Bpy^{2+}[**DB24C8**]-Trz^+ molecular shuttle calculated at C-PCM(CH_2Cl_2)/B3LYP(D3)/6-311G** level of theory.

BCP [a]	Distance (in Å)	$\rho(\boldsymbol{r})$	$\nabla^2\rho(\boldsymbol{r})$	$G(\boldsymbol{r})$	$V(\boldsymbol{r})$	$-G(\boldsymbol{r})/V(\boldsymbol{r})$	$H(\mathbf{r})$
$C(sp^2)$ -- H-$C(sp^3)$	2.80	0.0061	0.0187	0.0038	-0.0029	1.3114	0.0009
$C(sp^2)$ -- H-$C(sp^3)$	2.83	0.0060	0.0181	0.0036	-0.0028	1.3169	0.0009
$C(sp^2)$ -- H-$C(sp^3)$	2.86	0.0058	0.0216	0.0042	-0.0030	1.3875	0.0012
$C(sp^2)$ -- H-$C(sp^3)$	3.06	0.0040	0.0124	0.0025	-0.0019	1.3193	0.0006
$C(sp^2)$ -- O_{et}	2.84	0.0136	0.0527	0.0113	-0.0094	1.2018	0.0019
$C(sp^2)$ -- O_{et}	2.96	0.0107	0.0376	0.0082	-0.0069	1.1807	0.0012
$C(sp^2)$ -- O_{et}	2.98	0.0093	0.0333	0.0071	-0.0059	1.2029	0.0012
$C(sp^2)$ -- O_{et}	3.03	0.0084	0.0311	0.0066	-0.0053	1.2301	0.0012
$C(sp^2)$ -- O_{et}	3.06	0.0078	0.0311	0.0064	-0.0051	1.2645	0.0013
$C(sp^2)$ -- O_{et}	3.07	0.0083	0.0272	0.0058	-0.0049	1.1960	0.0010
$C(sp^2)$-H -- H-$C(sp^3)$	2.62	0.0029	0.0102	0.0020	-0.0015	1.3444	0.0005
$C(sp^2)$-H -- H-$C(sp^3)$	2.74	0.0023	0.0075	0.0015	-0.0011	1.3759	0.0004
$C(sp^2)$-H -- O_{et}	1.84	0.0336	0.1272	0.0300	-0.0282	1.0638	0.0018
$C(sp^2)$-H -- O_{et}	2.02	0.0229	0.0895	0.0195	-0.0167	1.1712	0.0029
$C(sp^2)$-H -- O_{et}	2.05	0.0222	0.0791	0.0175	-0.0152	1.1494	0.0023
$C(sp^2)$-H -- O_{et}	2.19	0.0168	0.0567	0.0124	-0.0106	1.1680	0.0018
$C(sp^2)$-H -- O_{et}	2.34	0.0149	0.0572	0.0123	-0.0103	1.1959	0.0020
$C(sp^2)$-H -- O_{et}	2.40	0.0129	0.0501	0.0107	-0.0088	1.2113	0.0019
$C(sp^2)$-H -- O_{et}	2.83	0.0061	0.0228	0.0047	-0.0037	1.2691	0.0010

Table S5. Electron density $\rho(\boldsymbol{r})$ ($e \cdot a_0^{-3}$), Laplacian of electron density $\nabla^2\rho(\boldsymbol{r})$ ($e \cdot a_0^{-5}$), electron kinetic energy density $G(\boldsymbol{r})$ (hartree$\cdot a_0^{-3}$), electron potential energy density $V(\boldsymbol{r})$ (hartree$\cdot a_0^{-3}$), and electron energy density $H(\mathbf{r})$ (hartree$\cdot a_0^{-3}$) for bond critical points on selected bonds of the Am-Bpy-Trz^+[**DB24C8**] molecular shuttle calculated at C-PCM(CH_2Cl_2)/B3LYP(D3)/6-311G** level of theory.

BCP [a]	Distance (in Å)	$\rho(\boldsymbol{r})$	$\nabla^2\rho(\boldsymbol{r})$	$G(\boldsymbol{r})$	$V(\boldsymbol{r})$	$-G(\boldsymbol{r})/V(\boldsymbol{r})$	$H(\mathbf{r})$
$C(sp^2)$ -- H-$C(sp^3)$	2.91	0.0055	0.0180	0.0036	-0.0027	1.3215	0.0009
$C(sp^2)$ -- H-$C(sp^3)$	3.56	0.0016	0.0049	0.0009	-0.0006	1.4660	0.0003
$C(sp^2)$ -- O_{et}	2.78	0.0141	0.0503	0.0113	-0.0101	1.1208	0.0012
$C(sp^2)$ -- O_{et}	3.03	0.0094	0.0348	0.0074	-0.0060	1.2192	0.0013
$C(sp^2)$ -- O_{et}	3.07	0.0088	0.0324	0.0069	-0.0056	1.2176	0.0012
$C(sp^2)$-H -- H-$C(sp^3)$	2.38	0.0047	0.0146	0.0030	-0.0024	1.2477	0.0006
$C(sp^2)$-H -- O_{et}	1.94	0.0275	0.0991	0.0226	-0.0203	1.1087	0.0022
$C(sp^3)$ -- O_{et}	2.99	0.0111	0.0428	0.0091	-0.0075	1.2122	0.0016
$C(sp^3)$-H -- H-$C(sp^3)$	2.09	0.0082	0.0212	0.0046	-0.0038	1.1947	0.0007
$C(sp^3)$-H -- H-$C(sp^3)$	2.26	0.0061	0.0158	0.0034	-0.0029	1.1711	0.0005
$C(sp^3)$-H -- H-$C(sp^3)$	2.87	0.0021	0.0069	0.0013	-0.0009	1.4271	0.0004
$C(sp^3)$-H -- H-$C(sp^3)$	3.02	0.0013	0.0046	0.0008	-0.0005	1.5708	0.0003
$C(sp^3)$-H -- O_{et}	2.08	0.0209	0.0722	0.0160	-0.0140	1.1465	0.0020
$C(sp^3)$-H -- O_{et}	2.10	0.0196	0.0701	0.0153	-0.0131	1.1689	0.0022
$C(sp^3)$-H -- O_{et}	2.10	0.0198	0.0727	0.0159	-0.0135	1.1726	0.0023
$C(sp^3)$-H -- O_{et}	2.22	0.0161	0.0559	0.0122	-0.0105	1.1685	0.0018
$C(sp^3)$-H -- O_{et}	2.48	0.0101	0.0290	0.0066	-0.0059	1.1172	0.0007
$C(sp^3)$-H -- O_{et}	2.73	0.0059	0.0204	0.0044	-0.0036	1.2093	0.0008
$N(sp^2)$ -- O_{et}	2.78	0.0134	0.0539	0.0118	-0.0101	1.1685	0.0017
$N_c(sp^2)$ -- H-$C(sp^3)$	2.74	0.0062	0.0193	0.0042	-0.0036	1.1771	0.0006
$N_c(sp^2)$ -- O_{et}	3.29	0.0048	0.0178	0.0037	-0.0030	1.2513	0.0007

WILEY-VCH

$N_c(sp^2)$ -- O_{et}	3.33	0.0048	0.0185	0.0039	-0.0031	1.2439	0.0008

Pasted below we report the XYZ coordinates of the relaxed structures investigated - AmH+[**DB24C8**]-Bpy^{2+}-Trz^{+}, Am-Bpy^{2+}[**DB24C8**]-Trz^{+} and Am-Bpy-Trz^{+}[**DB24C8**] - at C-PCM(CH_2Cl_2)/B3LYP(D3)/6-311G** level of theory.

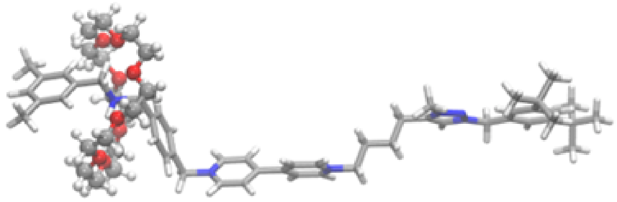

175
generated by VMD

C	-1.067000	3.240000	1.296000
C	-0.612000	4.000000	0.208000
C	-1.558000	4.553000	-0.669000
C	-2.906000	4.344000	-0.441000
N	-3.323000	3.607000	0.624000
C	-2.425000	3.056000	1.483000
C	0.839000	4.218000	-0.002000
C	1.676000	4.534000	1.079000
C	3.031000	4.712000	0.867000
N	3.558000	4.584000	-0.379000
C	2.768000	4.289000	-1.443000
C	1.406000	4.104000	-1.281000
C	5.080000	4.613000	-0.554000
C	5.546000	3.191000	-0.374000
C	5.565000	2.324000	-1.475000
C	5.781000	0.959000	-1.288000
C	5.953000	0.465000	0.008000
C	5.994000	1.330000	1.105000
C	5.790000	2.695000	0.915000
C	6.154000	-1.011000	0.216000
N	7.640000	-1.299000	-0.035000
C	8.017000	-2.748000	0.229000
C	9.482000	-3.000000	-0.063000
C	10.436000	-2.849000	0.949000
C	11.798000	-3.034000	0.683000
C	12.196000	-3.370000	-0.618000
C	11.257000	-3.531000	-1.644000
C	9.899000	-3.347000	-1.350000
C	12.821000	-2.875000	1.794000
C	11.695000	-3.911000	-3.049000
C	-4.802000	3.357000	0.826000
C	-5.249000	2.125000	0.017000
C	-6.761000	1.890000	0.221000
C	-7.236000	0.652000	-0.595000
C	-8.698000	0.416000	-0.409000
C	-9.769000	0.820000	-1.180000
N	-10.900000	0.374000	-0.552000
N	-10.620000	-0.291000	0.604000
N	-9.253000	-0.250000	0.654000
C	-8.568000	-0.947000	1.767000
H	5.279000	5.006000	-1.552000
H	5.473000	5.299000	0.197000
H	5.398000	2.712000	-2.475000
H	5.789000	0.266000	-2.120000
H	5.956000	-1.277000	1.255000
H	5.543000	-1.574000	-0.491000
H	7.786000	-2.934000	1.275000
H	7.369000	-3.358000	-0.405000
H	10.110000	-2.521000	1.927000
H	12.983000	-3.833000	2.303000

WILEY-VCH

H	13.784000	-2.536000	1.397000
H	12.477000	-2.151000	2.540000
H	13.252000	-3.497000	-0.838000
H	11.716000	-5.003000	-3.160000
H	11.006000	-3.509000	-3.798000
H	12.699000	-3.532000	-3.261000
H	9.177000	-3.461000	-2.151000
H	6.131000	0.907000	2.091000
H	5.791000	3.366000	1.767000
H	3.713000	4.947000	1.670000
H	1.282000	4.653000	2.078000
H	0.807000	3.846000	-2.142000
H	3.249000	4.185000	-2.403000
H	-1.257000	5.163000	-1.509000
H	-3.669000	4.760000	-1.082000
H	-4.958000	3.210000	1.897000
H	-5.327000	4.259000	0.506000
H	-5.036000	2.289000	-1.045000
H	-4.685000	1.246000	0.352000
H	-6.969000	1.730000	1.285000
H	-7.327000	2.769000	-0.105000
H	-7.042000	0.820000	-1.658000
H	-6.673000	-0.234000	-0.283000
H	-9.785000	1.372000	-2.102000
C	-12.299000	0.657000	-1.002000
H	-8.144000	-1.880000	1.394000
H	-7.786000	-0.297000	2.159000
H	-9.315000	-1.145000	2.533000
H	-2.819000	2.481000	2.306000
H	-0.375000	2.781000	1.988000
H	7.922000	-0.987000	-1.007000
H	8.183000	-0.642000	0.587000
C	-13.379000	-0.325000	-0.564000
H	-12.528000	1.663000	-0.640000
H	-12.246000	0.677000	-2.094000
C	-14.691000	0.119000	-0.752000
C	-15.784000	-0.708000	-0.452000
C	-15.520000	-1.988000	0.046000
C	-14.211000	-2.456000	0.240000
C	-13.141000	-1.612000	-0.078000
H	-14.865000	1.119000	-1.136000
C	-17.215000	-0.183000	-0.674000
H	-16.344999	-2.640000	0.292000
C	-13.990000	-3.874000	0.800000
H	-12.128000	-1.947000	0.066000
C	-12.490000	-4.216000	0.956000
C	-14.661000	-3.978000	2.196000
C	-14.630000	-4.908000	-0.164000
C	-18.288000	-1.227000	-0.289000
C	-17.434000	1.084000	0.194000
C	-17.398001	0.173000	-2.174000
H	-14.515000	-4.986000	2.601000
H	-14.214000	-3.252000	2.884000
H	-15.736000	-3.783000	2.132000
H	-12.397000	-5.229000	1.361000
H	-11.975000	-4.186000	-0.011000
H	-11.995000	-3.524000	1.647000
H	-14.485000	-5.920000	0.232000
H	-15.705000	-4.731000	-0.275000
H	-14.160000	-4.844000	-1.151000
H	-18.448999	1.466000	0.037000
H	-17.306999	0.841000	1.254000
H	-16.723000	1.873000	-0.073000
H	-19.281000	-0.800000	-0.469000
H	-18.191999	-2.136000	-0.893000
H	-18.218000	-1.494000	0.772000
H	-18.413000	0.551000	-2.341000
H	-16.687000	0.944000	-2.487000
H	-17.246000	-0.717000	-2.794000
O	8.974000	-0.870000	2.715000
O	6.501000	-1.412000	-3.440000
O	4.010000	-1.470000	-2.285000
O	2.601000	-0.068000	-0.185000
O	6.152000	-1.242000	3.423000
O	3.782000	-0.204000	2.283000
O	9.028000	0.900000	0.685000
O	8.612000	0.003000	-2.183000

C	7.263000	-2.047000	3.947000
H	7.500000	-2.882000	3.280000
H	7.011000	-2.445000	4.937000
C	8.459000	-1.124000	4.066000
H	8.126000	-0.187000	4.530000
H	9.234000	-1.582000	4.694000
C	9.989000	0.192000	2.732000
H	10.888000	-0.128000	3.275000
H	9.575000	1.089000	3.209000
C	9.142000	1.722000	-0.530000
H	8.134000	2.086000	-0.720000
H	9.814000	2.568000	-0.336000
C	7.901000	-1.579000	-3.837000
H	7.988000	-1.901000	-4.882000
H	8.394000	-2.305000	-3.186000
C	5.825000	-2.701000	-3.257000
H	6.085000	-3.135000	-2.283000
H	6.104000	-3.412000	-4.046000
C	4.338000	-2.427000	-3.342000
H	4.115000	-1.992000	-4.325000
H	3.776000	-3.364000	-3.233000
C	2.610000	-1.037000	-2.375000
H	1.939000	-1.802000	-1.964000
H	2.338000	-0.851000	-3.423000
C	2.463000	0.257000	-1.598000
H	3.244000	0.956000	-1.918000
H	1.478000	0.705000	-1.806000
C	2.658000	1.133000	0.635000
H	1.794000	1.792000	0.453000
H	3.575000	1.692000	0.444000
C	2.634000	0.674000	2.082000
H	2.674000	1.554000	2.742000
H	1.698000	0.131000	2.278000
C	4.836000	-1.856000	3.660000
H	4.821000	-2.356000	4.637000
H	4.600000	-2.580000	2.873000
C	3.816000	-0.734000	3.643000
H	2.833000	-1.134000	3.933000
H	4.114000	0.039000	4.363000
C	10.324000	0.519000	1.289000
H	11.026000	1.359000	1.254000
H	10.747000	-0.350000	0.780000
C	9.639000	0.972000	-1.763000
H	10.584000	0.448000	-1.581000
H	9.787000	1.712000	-2.558000
C	8.600000	-0.251000	-3.632000
H	8.069000	0.559000	-4.142000
H	9.626000	-0.321000	-4.012000

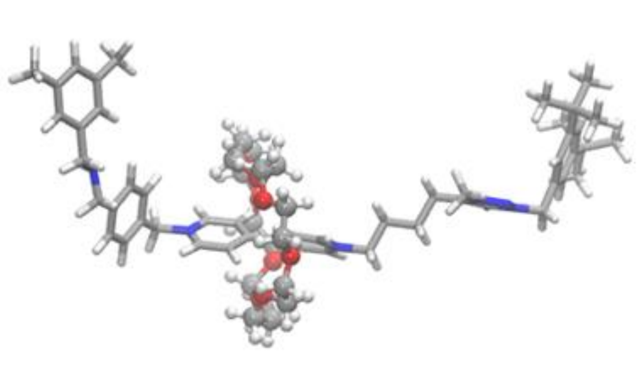

174

generated by VMD

C	1.060000	-1.260000	-1.376000
C	1.321000	-2.264000	-0.437000
C	0.272000	-3.100000	-0.021000
C	-0.993000	-2.905000	-0.541000
N	-1.226000	-1.938000	-1.475000
C	-0.216000	-1.133000	-1.897000
C	2.705000	-2.462000	0.050000
C	3.777000	-2.338000	-0.853000
C	5.072000	-2.469000	-0.401000

N	5.322000	-2.719000	0.914000
C	4.299000	-2.859000	1.796000
C	2.979000	-2.751000	1.392000
C	6.763000	-2.893000	1.388000
C	7.686000	-1.886000	0.745000
C	7.625000	-0.533000	1.111000
C	8.474000	0.395000	0.515000
C	9.403000	-0.008000	-0.455000
C	9.472000	-1.360000	-0.811000
C	8.617000	-2.293000	-0.220000
C	10.299000	1.019000	-1.134000
N	10.548000	2.184000	-0.253000
C	11.217000	3.316000	-0.945000
C	11.337000	4.529000	-0.031000
C	12.425000	5.396000	-0.149000
C	12.521000	6.543000	0.657000
C	11.511000	6.798000	1.589000
C	10.413000	5.933000	1.729000
C	10.336000	4.802000	0.912000
C	13.709000	7.480000	0.513000
C	9.335000	6.229000	2.759000
C	-2.616000	-1.746000	-2.023000
C	-3.411000	-0.745000	-1.164000
C	-4.840000	-0.576000	-1.727000
C	-5.657000	0.428000	-0.861000
C	-7.050000	0.600000	-1.377000
C	-8.215000	-0.048000	-1.010000
N	-9.205000	0.437000	-1.812000
N	-8.752000	1.381000	-2.687000
N	-7.415000	1.452000	-2.388000
C	-6.575000	2.411000	-3.143000
H	6.734000	-2.784000	2.473000
H	7.052000	-3.917000	1.136000
H	6.915000	-0.209000	1.864000
H	8.450000	1.440000	0.795000
H	11.221000	0.525000	-1.484000
H	9.783000	1.399000	-2.026000
H	12.215000	3.057000	-1.337000
H	10.590000	3.573000	-1.810000
H	13.208000	5.182000	-0.870000
H	13.733000	7.927000	-0.488000
H	13.660000	8.289000	1.248000
H	14.652000	6.940000	0.658000
H	11.579000	7.679000	2.221000
H	8.905000	7.225000	2.599000
H	8.528000	5.493000	2.702000
H	9.750000	6.206000	3.774000
H	9.508000	4.110000	1.004000
H	10.197000	-1.687000	-1.548000
H	8.683000	-3.339000	-0.500000
H	5.933000	-2.377000	-1.047000
H	3.573000	-2.209000	-1.910000
H	2.211000	-2.874000	2.145000
H	4.562000	-3.029000	2.829000
H	0.453000	-3.921000	0.670000
H	-1.835000	-3.519000	-0.256000
H	-2.509000	-1.388000	-3.049000
H	-3.097000	-2.726000	-2.034000
H	-3.461000	-1.111000	-0.132000
H	-2.894000	0.222000	-1.161000
H	-4.790000	-0.214000	-2.761000
H	-5.356000	-1.542000	-1.732000
H	-5.715000	0.056000	0.166000
H	-5.146000	1.396000	-0.838000
H	-8.381000	-0.791000	-0.252000
C	-10.674000	0.136000	-1.784000
H	-6.150000	3.136000	-2.448000
H	-5.786000	1.865000	-3.662000
H	-7.225000	2.909000	-3.860000
H	-0.422000	-0.431000	-2.690000
H	1.819000	-0.565000	-1.704000
H	11.094000	1.901000	0.570000
C	-11.350000	0.892000	-0.656000
H	-11.046000	0.434000	-2.765000
H	-10.772000	-0.944000	-1.665000
C	-11.401000	2.285000	-0.694000
C	-12.034000	3.006000	0.331000

WILEY-VCH

C	-12.609000	2.280000	1.382000
C	-12.572000	0.875000	1.443000
C	-11.928000	0.189000	0.407000
H	-10.941000	2.808000	-1.526000
C	-12.077000	4.546000	0.263000
H	-13.103000	2.818000	2.179000
C	-13.232000	0.149000	2.633000
H	-11.877000	-0.892000	0.414000
C	-13.089000	-1.388000	2.533000
C	-12.561000	0.615000	3.954000
C	-14.745000	0.495000	2.666000
C	-12.815000	5.168000	1.472000
C	-10.625000	5.097000	0.242000
C	-12.816000	4.979000	-1.033000
H	-13.033000	0.109000	4.804000
H	-11.494000	0.368000	3.946000
H	-12.669000	1.695000	4.093000
H	-13.574000	-1.847000	3.401000
H	-13.572000	-1.775000	1.628000
H	-12.035000	-1.690000	2.532000
H	-15.220000	-0.010000	3.516000
H	-14.903000	1.573000	2.775000
H	-15.232000	0.162000	1.743000
H	-10.647000	6.191000	0.184000
H	-10.095000	4.805000	1.156000
H	-10.070000	4.717000	-0.622000
H	-12.812000	6.258000	1.366000
H	-13.857000	4.833000	1.518000
H	-12.317000	4.915000	2.415000
H	-12.843000	6.073000	-1.094000
H	-12.308000	4.596000	-1.924000
H	-13.845000	4.602000	-1.028000
O	2.679000	-0.940000	3.564000
O	1.572000	-0.256000	-4.013000
O	2.513000	-3.033000	-3.421000
O	2.057000	-5.187000	-1.112000
O	1.416000	-3.490000	3.932000
O	0.948000	-5.369000	1.686000
O	2.393000	0.391000	0.835000
O	2.699000	1.418000	-1.980000
C	2.081000	-2.752000	5.014000
H	3.154000	-2.980000	5.043000
H	1.635000	-3.019000	5.981000
C	1.894000	-1.267000	4.751000
H	0.830000	-1.048000	4.593000
H	2.245000	-0.703000	5.626000
C	2.723000	0.498000	3.288000
H	3.326000	1.011000	4.050000
H	1.715000	0.934000	3.290000
C	1.766000	1.613000	0.309000
H	0.811000	1.301000	-0.121000
H	1.573000	2.338000	1.111000
C	2.153000	1.060000	-4.287000
H	1.709000	1.507000	-5.186000
H	3.238000	0.978000	-4.427000
C	1.975000	-1.261000	-4.999000
H	3.062000	-1.240000	-5.148000
H	1.481000	-1.073000	-5.962000
C	1.586000	-2.646000	-4.494000
H	0.551000	-2.638000	-4.136000
H	1.668000	-3.351000	-5.330000
C	2.779000	-4.476000	-3.349000
H	3.750000	-4.577000	-2.858000
H	2.845000	-4.882000	-4.366000
C	1.765000	-5.301000	-2.550000
H	0.736000	-4.986000	-2.765000
H	1.877000	-6.348000	-2.865000
C	1.655000	-6.401000	-0.391000
H	2.204000	-7.268000	-0.785000
H	0.579000	-6.592000	-0.491000
C	1.976000	-6.234000	1.090000
H	1.948000	-7.221000	1.569000
H	2.974000	-5.801000	1.206000
C	1.596000	-4.941000	4.069000
H	1.333000	-5.259000	5.087000
H	2.638000	-5.227000	3.874000
C	0.639000	-5.618000	3.095000

WILEY-VCH

H	0.623000	-6.697000	3.293000
H	-0.360000	-5.204000	3.248000
C	3.356000	0.679000	1.910000
H	3.759000	1.694000	1.821000
H	4.177000	-0.034000	1.806000
C	2.640000	2.255000	-0.767000
H	3.668000	2.355000	-0.405000
H	2.258000	3.254000	-1.008000
C	1.879000	1.940000	-3.079000
H	0.810000	1.919000	-2.835000
H	2.167000	2.974000	-3.311000

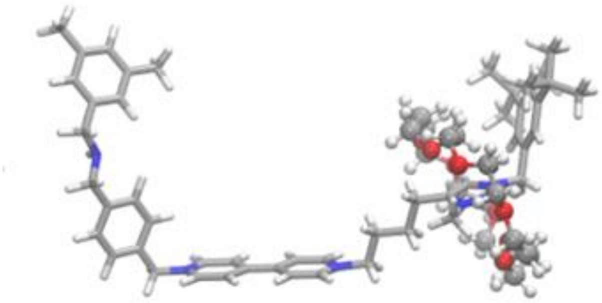

174
generated by VMD

C	-3.883000	-4.473000	0.178000
C	-4.617000	-4.136000	-1.048000
C	-3.729000	-3.808000	-2.171000
C	-2.381000	-3.815000	-2.056000
N	-1.731000	-4.148000	-0.863000
C	-2.532000	-4.466000	0.240000
C	-5.999000	-4.139000	-1.141000
C	-6.886000	-4.472000	-0.019000
C	-8.233000	-4.471000	-0.133000
N	-8.883000	-4.156000	-1.334000
C	-8.083000	-3.835000	-2.436000
C	-6.733000	-3.818000	-2.371000
C	-10.353000	-4.189000	-1.414000
C	-11.029000	-3.086000	-0.603000
C	-10.490000	-1.793000	-0.553000
C	-11.141000	-0.782000	0.153000
C	-12.345000	-1.039000	0.823000
C	-12.884000	-2.330000	0.771000
C	-12.229000	-3.345000	0.069000
C	-13.028000	0.058000	1.627000
N	-12.792000	1.392000	1.023000
C	-13.315000	2.511000	1.845000
C	-12.936000	3.860000	1.248000
C	-13.785000	4.960000	1.387000
C	-13.423000	6.218000	0.878000
C	-12.196000	6.351000	0.220000
C	-11.331000	5.255000	0.063000
C	-11.711000	4.015000	0.581000
C	-14.357000	7.406000	1.041000
C	-10.008000	5.426000	-0.666000
C	-0.270000	-4.011000	-0.726000
C	0.157000	-2.601000	-0.260000
C	1.690000	-2.494000	-0.127000
C	2.126000	-1.091000	0.366000
C	3.609000	-0.938000	0.424000
C	4.467000	-0.417000	-0.528000
N	5.713000	-0.485000	0.008000
N	5.728000	-1.034000	1.261000
N	4.399000	-1.308000	1.472000
C	4.000000	-1.908000	2.765000
H	-10.618000	-4.088000	-2.472000
H	-10.717000	-5.166000	-1.074000
H	-9.557000	-1.584000	-1.063000
H	-10.739000	0.223000	0.187000
H	-14.098000	-0.187000	1.742000
H	-12.598000	0.087000	2.637000
H	-14.408000	2.472000	1.990000
H	-12.857000	2.414000	2.839000

WILEY-VCH

H	-14.739000	4.842000	1.892000
H	-14.512000	7.637000	2.102000
H	-13.946000	8.295000	0.554000
H	-15.338000	7.194000	0.600000
H	-11.909000	7.319000	-0.181000
H	-9.399000	6.205000	-0.192000
H	-9.438000	4.492000	-0.662000
H	-10.174000	5.722000	-1.709000
H	-11.074000	3.147000	0.465000
H	-13.817000	-2.544000	1.282000
H	-12.656000	-4.343000	0.042000
H	-8.883000	-4.710000	0.698000
H	-6.465000	-4.730000	0.943000
H	-6.192000	-3.560000	-3.272000
H	-8.619000	-3.604000	-3.348000
H	-4.149000	-3.556000	-3.135000
H	-1.731000	-3.577000	-2.889000
H	0.078000	-4.761000	-0.007000
H	0.185000	-4.236000	-1.696000
H	-0.206000	-1.859000	-0.982000
H	-0.316000	-2.387000	0.707000
H	2.051000	-3.259000	0.573000
H	2.159000	-2.693000	-1.099000
H	1.764000	-0.309000	-0.306000
H	1.709000	-0.888000	1.357000
H	4.253000	-0.028000	-1.513000
C	7.010000	-0.216000	-0.675000
H	4.063000	-1.131000	3.528000
H	2.982000	-2.278000	2.650000
H	4.705000	-2.716000	2.949000
H	-1.996000	-4.721000	1.146000
H	-4.424000	-4.748000	1.073000
H	-13.191000	1.430000	0.077000
C	7.210000	1.268000	-0.899000
H	7.775000	-0.634000	-0.021000
H	6.947000	-0.780000	-1.606000
C	7.752000	2.070000	0.104000
C	7.930000	3.451000	-0.093000
C	7.556000	3.990000	-1.330000
C	7.005000	3.204000	-2.360000
C	6.834000	1.837000	-2.122000
H	8.039000	1.614000	1.045000
C	8.526000	4.309000	1.042000
H	7.692000	5.049000	-1.504000
C	6.605000	3.860000	-3.697000
H	6.383000	1.203000	-2.872000
C	6.060000	2.825000	-4.712000
C	5.501000	4.919000	-3.432000
C	7.844000	4.552000	-4.327000
C	8.632000	5.803000	0.658000
C	7.621000	4.194000	2.299000
C	9.951000	3.791000	1.380000
H	5.215000	5.403000	-4.373000
H	4.616000	4.438000	-3.003000
H	5.850000	5.688000	-2.736000
H	5.750000	3.350000	-5.622000
H	6.840000	2.104000	-4.986000
H	5.202000	2.273000	-4.314000
H	7.562000	5.021000	-5.277000
H	8.245000	5.327000	-3.666000
H	8.632000	3.815000	-4.520000
H	8.039000	4.797000	3.112000
H	6.612000	4.559000	2.074000
H	7.548000	3.156000	2.641000
H	9.055000	6.358000	1.502000
H	9.289000	5.946000	-0.207000
H	7.647000	6.225000	0.430000
H	10.377000	4.388000	2.195000
H	9.926000	2.743000	1.696000
H	10.603000	3.877000	0.504000
O	2.369000	1.735000	-0.939000
O	6.495000	-1.660000	4.395000
O	6.320000	-4.019000	2.618000
O	6.372000	-3.137000	-0.523000
O	3.776000	0.509000	-3.314000
O	5.715000	-1.789000	-2.977000
O	2.995000	1.283000	1.975000

WILEY-VCH

O	4.429000	0.554000	4.698000
C	2.432000	1.124000	-3.283000
H	1.682000	0.386000	-2.975000
H	2.182000	1.493000	-4.284000
C	2.437000	2.280000	-2.297000
H	3.347000	2.875000	-2.438000
H	1.568000	2.923000	-2.493000
C	2.406000	2.800000	0.070000
H	1.681000	3.587000	-0.186000
H	3.404000	3.255000	0.109000
C	4.141000	1.936000	2.633000
H	5.040000	1.402000	2.313000
H	4.225000	2.984000	2.324000
C	6.229000	-0.827000	5.566000
H	7.127000	-0.757000	6.196000
H	5.403000	-1.221000	6.173000
C	6.928000	-3.011000	4.747000
H	6.102000	-3.606000	5.158000
H	7.740000	-2.975000	5.487000
C	7.453000	-3.665000	3.477000
H	8.128000	-2.957000	2.984000
H	8.019000	-4.571000	3.739000
C	6.749000	-4.710000	1.393000
H	5.847000	-5.173000	0.986000
H	7.469000	-5.498000	1.656000
C	7.375000	-3.782000	0.336000
H	7.913000	-2.971000	0.831000
H	8.086000	-4.354000	-0.274000
C	6.148000	-3.859000	-1.774000
H	5.754000	-4.869000	-1.594000
H	7.081000	-3.943000	-2.347000
C	5.125000	-3.081000	-2.590000
H	4.873000	-3.665000	-3.485000
H	4.222000	-2.925000	-1.993000
C	3.781000	-0.694000	-4.167000
H	3.464000	-0.426000	-5.184000
H	3.080000	-1.438000	-3.770000
C	5.199000	-1.253000	-4.238000
H	5.222000	-2.029000	-5.016000
H	5.888000	-0.452000	-4.514000
C	2.014000	2.223000	1.431000
H	1.833000	3.056000	2.124000
H	1.089000	1.653000	1.317000
C	3.994000	1.855000	4.157000
H	2.938000	1.956000	4.420000
H	4.563000	2.661000	4.637000
C	5.855000	0.559000	5.059000
H	6.492000	0.795000	4.198000
H	6.027000	1.305000	5.846000

In the figure reported below we report the IGMH plot derived at C-PCM(CH_2Cl_2)/B3LYP(D3)/6-311G** level of theory and explicitly reported into the manuscript as Figure 5 (lower panel).

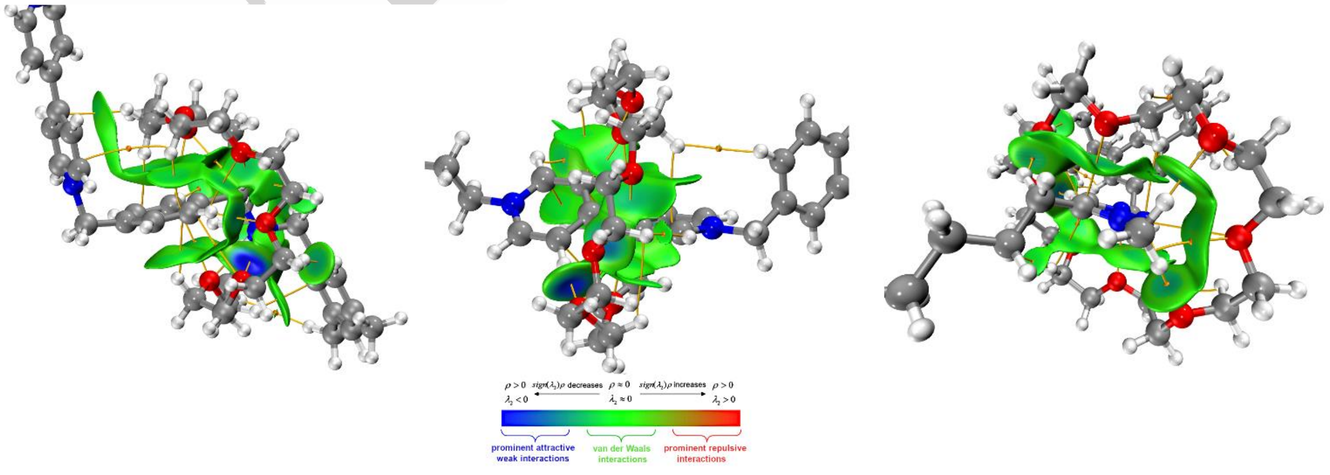

WILEY-VCH

Always remaining in this context, for the sake of completeness, at the following links over Google Drive Platform, we share three different videos showing the local supramolecular contacts - appreciated by means of a QTAIM+IGMH analysis - between the **DB24C8** macrocycle and the AmH^{+}, Bpy^{2+} and Trz^{+} stations, respectively.

AmH^{+}[**DB24C8**]-Bpy^{2+}-Trz^{+}:
https://drive.google.com/file/d/1aoyi6f353MXzHyiAolRWhSC1rKlphOFT/view?usp=sharing

Am-Bpy^{2+}[**DB24C8**]-Trz^{+}:
https://drive.google.com/file/d/1SR1w32LcIkxyjklwqz39QkfDEdpuEyKi/view?usp=sharing

Am-Bpy-Trz^{+}[**DB24C8**]:
https://drive.google.com/file/d/1LCiLh9ASkSrXSgQkrtlbzdjcjgPcwnvB/view?usp=sharing

WILEY-VCH
